# Wnt/β-catenin signaling components and mechanisms in bone formation, homeostasis, and disease

**DOI:** 10.1038/s41413-024-00342-8

**Published:** 2024-07-10

**Authors:** Lifang Hu, Wei Chen, Airong Qian, Yi-Ping Li

**Affiliations:** 1 Laboratory for Bone Metabolism, Xi’an Key Laboratory of Special Medicine and Health Engineering, Key Laboratory for Space Biosciences and Biotechnology, Research Center for Special Medicine and Health Systems Engineering, School of Life Sciences, Northwestern Polytechnical University, Xi’an, Shaanxi 710072 China; 2 Division in Cellular and Molecular Medicine, Department of Pathology and Laboratory Medicine, Tulane University School of Medicine, Tulane University, New Orleans, LA 70112 USA

**Keywords:** Bone, Homeostasis

## Abstract

Wnts are secreted, lipid-modified proteins that bind to different receptors on the cell surface to activate canonical or non-canonical Wnt signaling pathways, which control various biological processes throughout embryonic development and adult life. Aberrant Wnt signaling pathway underlies a wide range of human disease pathogeneses. In this review, we provide an update of Wnt/β-catenin signaling components and mechanisms in bone formation, homeostasis, and diseases. The Wnt proteins, receptors, activators, inhibitors, and the crosstalk of Wnt signaling pathways with other signaling pathways are summarized and discussed. We mainly review Wnt signaling functions in bone formation, homeostasis, and related diseases, and summarize mouse models carrying genetic modifications of Wnt signaling components. Moreover, the therapeutic strategies for treating bone diseases by targeting Wnt signaling, including the extracellular molecules, cytosol components, and nuclear components of Wnt signaling are reviewed. In summary, this paper reviews our current understanding of the mechanisms by which Wnt signaling regulates bone formation, homeostasis, and the efforts targeting Wnt signaling for treating bone diseases. Finally, the paper evaluates the important questions in Wnt signaling to be further explored based on the progress of new biological analytical technologies.

## Introduction

Wnt signaling, an evolutionarily conserved signaling pathway from nematodes to mammals, plays key roles in regulating multiple biological processes, including embryonic development, organogenesis, tissue homeostasis in adults, and numerous diseases.^1,2^ The term “Wnt” is the combination of the terms “wingless” and “int”.^3^ *Int-1*, which was later renamed *Wnt1*, is the first Wnt gene discovered in mouse breast tumors induced by mouse mammary tumor virus in 1982.^4^ The *wingless* gene, which controls segment pattern during the development of *Drosophila* larva,^5^ was identified as the homolog of *Wnt1* (*int-1*) in 1987.^6^ Later, researchers demonstrated the Wnt signal transduction in *Drosophila* by delineating the components of the signaling, including zeste-white 3 (the homolog of mammalian glycogen synthase kinase 3 (GSK3)), disheveled (Dvl), and armadillo (the homolog of vertebrate β-catenin).^7–9^ Further, injection of *Xenopus* eggs with mouse Wnt1 RNA caused duplication of the embryonic axis, suggesting a critical role of Wnt signaling in vertebrate development.^10^ These foundational studies reveal the importance of Wnt signaling in development. Since these pioneering discoveries, there is an explosion of research on the Wnt signaling pathway, ranging from signal transduction and complex regulation to its role in normal development and diseases.

There are 19 known Wnt proteins that either function through the canonical Wnt signaling pathway or the non-canonical Wnt signaling pathway. The best-studied Wnt signaling pathway is the canonical Wnt signaling pathway, which is also referred to as the Wnt/β-catenin signaling pathway for its dependency on β-catenin transcriptional function. Otherwise, the non-canonical Wnt signaling pathway is a β-catenin independent pathway. There are two well-known non-canonical Wnt signaling pathways: the Wnt/planar cell polarity (PCP) pathway and the Wnt/Ca^2+^ pathway. Both canonical and non-canonical Wnt signaling pathways play crucial roles in normal tissue development and homeostasis.^11–13^ Moreover, the Wnt signaling pathway plays key roles in the pathogenesis of human diseases. In the early 1990s, the Kinzler and Nishisho groups independently found the adenomatous polyposis coli (APC) gene in familial adenomatous polyposis, a hereditary cancer syndrome, which was the first connection of the Wnt signaling pathway to human disease.^14,15^ Later, the APC protein was found to interact with β-catenin^16,17^ and APC deficiency resulted in constitutively active β-catenin/TCF (T-cell factor) signaling in colon carcinoma cells.^18^ These findings demonstrated the close link between Wnt signaling and human disease. After decades of research, evidence show that Wnt signaling pathways is involved in many human diseases, including numerous tissue diseases, metabolic diseases, and cancers.^2,19–21^

As an evolutionarily conserved complex signaling pathway, Wnt signaling is involved in multiple key events during embryo development and adult tissue homeostasis. Wnt signaling shows versatility not only in the signaling components but also in its physiological and pathological functions. This review summarizes the current knowledge of Wnt signaling pathways and reviews the advances of Wnt signaling pathways in bone formation, homeostasis, and disease. Furthermore, the therapeutic treatment of bone disease by targeting Wnt signaling is also discussed. Overall, this review will provide researchers with a comprehensive understanding of Wnts, the role of Wnt signaling pathways in bone homeostasis and disease, and therapeutics that target Wnt signaling and will serve as a reference for future studies.

## Wnt proteins, wnt receptors, and wnt signaling pathway

### Wnt proteins (Wnts)

The Wnt proteins (Wnts) act as intercellular signals and regulate a wide range of cellular behavior including cell fate specification, cell proliferation, survival, migration, polarity, and differentiation.^19^ Wnt proteins are conserved in all metazoan animals along with multiple associated genes. There are 19 in mammals, 16 in *Xenopus*, 11 in chicks, 12 in zebrafish, 7 in *Drosophila*, and 5 in *C. elegans* (http://www.stanford.edu/rnusse/wntwindow.html). Wnts encoded by Wnt genes are ~40 kD-secreted glycoproteins that are structurally related, containing 23 or 24 cysteine residues.^22^ The demonstration of Wnts as a lipid-modified protein was confirmed through the first successful purification of Wnt3a.^23^ The lipid modification involves the attachment of a palmitoleic acid (a mono-unsaturated fatty acid) to a highly conserved serine residue.^24^ The palmitoylation of Wnt is required for its binding to the Frizzled (Fzd) receptor, initiating signal transduction, and the glycosylation of Wnt that is necessary for its eventual secretion.^25^

Wnts were historically categorized as either canonical or noncanonical Wnts.^26^ However, the distinction is questionable because some Wnts can stimulate both canonical and noncanonical Wnt signaling pathways and typically noncanonical Wnts can activate canonical Wnt signaling.^27–30^

### Wnt receptors and co-receptors

Wnt signal transduction involves the binding of Wnts to cell-surface Wnt receptors and co-receptors, which primarily contain members of the Fzd family^31^ and the low-density lipoprotein receptor-related protein (LRP) family.^32^ Further, the receptor tyrosine kinase-like orphan receptor-1 (ROR1), ROR2,^33^ an atypical receptor tyrosine kinase (Ryk),^34,35^ protein tyrosine kinase 7 (PTK7),^36^ muscle-specific kinase (MuSK),^37^ and other molecules were also demonstrated as Wnt co-receptors.

#### Frizzled (Fzd) proteins

**Fig. 1:**
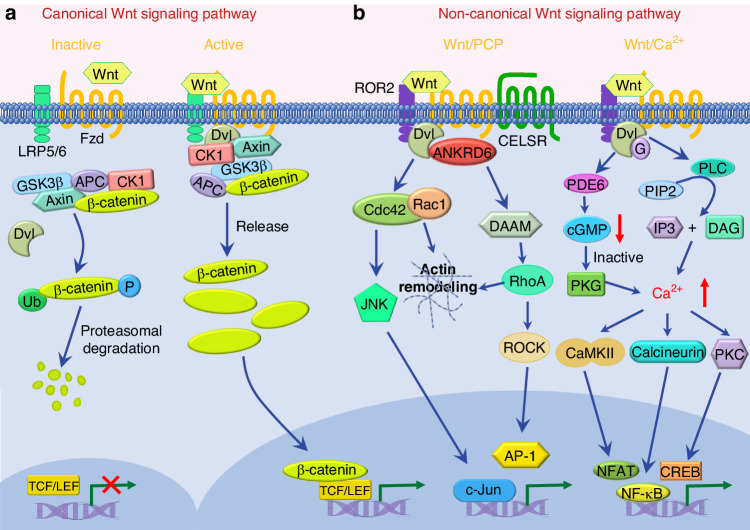
Schematic representation of canonical and non-canonical Wnt signaling pathway. **a** The canonical Wnt signaling pathway in inactive and active status. Without Wnt binding, β-catenin is sequestered by a destruction complex composed of GSK3β, Axin, APC, and CK1, which leads to the phosphorylation of β-catenin at serine/threonine residues. Phosphorylated β-catenin is then undergoing the proteosomal degradation mediated by polyubiquitination. The Wnt signaling is in inactive status. When Wnt binds to its receptor complex, including the seven-transmembrane receptor Fzd and the co-receptor LRP5 or LRP6, Wnt/β-catenin is initiated. This binding mobilizes GSK3β and CK1 to the cell membrane, where they phosphorylate serines on Lrp5/6, promoting the formation of a signalosome, and the recruitment of Dvl and Axin. Then, β-catenin is released from the destruction complex, accumulates in the cytoplasm and translocates into the nucleus to activate target gene expression by binding to TCF/LEF. Thus, The Wnt signaling is in active status. **b** Non-canonical Wnt signaling pathway includes Wnt/PCP and Wnt/Ca^2+^ signaling pathway. In the Wnt/PCP signaling pathway, non canonical Wnts bind to Fzd and the coreceptor (e.g., ROR2) to initiate the signaling. The Dvl is recruited to Fzds, which further activates the small GTPases Rac1 and RhoA. The activated GTPases induces changes in the actin cytoskeleton, and activates JNK and ROCK to regulate downstream signals. In the Wnt/Ca^2+^ signaling pathway, Wnts bind to Fzd to mediate the activation of a G protein, which in turn activate the PLC. The activated PLC leads to the generation of IP3 and DAG, which increase intracellular Ca^2+^ concentration. Alternatively, Wnt/Fzd activates cGMP-specific PDE6, which results in decrease of cGMP and the inactivation of PKG, thus increases intracellular Ca^2+^ concentration. The Ca^2+^ activates CaMKII, calcineurin, or PKC, which further activates various transcription factors

Fzd proteins are widespread high-affinity Wnt receptors. From the first Wnt receptor identified,^38^ the Fzd family now contains 10 members in humans, Fzd1 to Fzd10.^39^ They are seven-transmembrane receptors containing a large extracellular N-terminal cysteine-rich domain (CRD), which is conserved among the receptor family and mediates high-affinity binding to Wnts.^31,40–42^ Fzds transmit signaling through both canonical and non-canonical Wnt signaling pathways by cooperating with other co-receptors (Fig. 1). In the Wnt/β-catenin signaling pathway, Wnt–Fzd form a ternary complex with low-density lipoprotein receptor-related protein 5/6 (LRP5/6), whereas, in Wnt/PCP signaling pathway, Wnt–Fzd interact with ROR^32,33,43^ (see below: Wnt signaling pathways, Fig. 1).

#### Low-density lipoprotein receptor-related proteins (LRPs)

The LRP family is an evolutionarily conserved single-pass transmembrane receptor family. Beyond the Fzds, the members of the LRP family are required for Wnt signaling. Arrow in *Drosophila* and LRP5 and LRP6 in vertebrates are identified to function as co-receptors in the Wnt signaling pathway.^32,44,45^ The primary structures of LRP5 and LRP6 are more than 70% identical to each other and are widely co-expressed during embryogenesis and in adult tissues.^46,47^ The intracellular domain of LRP5 interacts with Axin and stabilizes β-catenin, and thus induces lymphoid enhancer factor 1 (LEF-1) activation.^48^ LRP6 is the best-studied LRP. The extracellular domain (ECD) of LRP6 mediates the interaction with Wnt and Fzd, resulting in ternary complex formation. The ECD contains multiple independent Wnt-binding sites to allow different Wnts to bind simultaneously in conjunction with Fzd.^49^

#### Receptor tyrosine kinase-like orphan receptor -1 and -2 (ROR1 and ROR2)

The receptors of the Ror family are membrane-spanning tyrosine kinases that bind Wnts either alone or as Fzds co-receptors to activate non-canonical Wnt signaling.^33,50^ ROR1 and ROR2 are also co-receptors for Wnt5a and mostly transduce Wnt/PCP signaling.^51–53^ Wnt5a can induce ROR1/ROR2 hetero-oligomerization.^54^ Wnt5a-ROR1/ROR2 signaling is involved in tissue development and cancer.^55–57^ Moreover, via ROR2, Wnt5a inhibits Wnt3a-induced Wnt/β-catenin signaling.^58^

#### Receptor related to tyrosine kinases (Ryk)

Ryk is an atypical member of the receptor tyrosine kinase (RTK) family,^59^ showing no detectable intrinsic protein tyrosine kinase activity.^60^ It is a single-pass transmembrane protein that contains an extracellular Wnt inhibitory factor (WIF) domain, an intracellular atypical kinase domain, and a PSD95/DlgA/ZO-1 (PDZ) binding motif.^60^ It was previously shown that Derailed, the Ryk homolog in *Drosophila*, is another receptor for Wnt, which binds to Wnt5a in the absence of Fzd or Dvl.^34^ However, Lu et al. demonstrated that mammalian Ryk, unlike Derailed, functions as a co-receptor along with Fzd for Wnts.^35^ Furthermore, Ryk also binds to Dvl, providing a link between Wnt and Dvl, thereby activating the canonical Wnt signaling pathway.^35^

#### Protein tyrosine kinase 7 (PTK7)

PTK7, originally identified as colon carcinoma kinase 4,^61^ is another single-pass transmembrane Wnt receptor.^36^ It contains seven extracellular immunoglobulin domains, a transmembrane domain, and an intracellular catalytically inactive tyrosine kinase domain, which serves as an interaction site for several intracellular signaling molecules (e.g., Dvl, β-catenin).^62,63^ PTK7 can interact with Wnt3a, Wnt8, Fzd7, LRP6, and ROR2,^64–66^ suggesting its involvement in both canonical and non-canonical Wnt signaling pathways.^67^ The up-regulation of the PCP signaling pathway by PTK7 is well established in current literature. PTK7 cooperates with Fzd to recruit Dvl to the plasma membrane to activate the Wnt/PCP signaling.^62^ However, there are conflicting findings on the function of PTK7 in canonical Wnt (Wnt/β-catenin) signaling. Peradziryi et al. reported that PTK7 inhibits Wnt/β-catenin signaling in *Xenopus* and *Drosophila* model systems.^64^ The inhibition effect of PTK7 on Wnt/β-catenin signaling was also observed in zebrafish during late gastrula and segmentation stages.^68^ In contrast, Puppo et al. found that PTK7 interacts with β-catenin in a yeast two-hybrid assay, and mammalian cells and PTK7-deficient cells show weakened Wnt/β-catenin activity.^63^ Bin-Nun and colleagues also reported that PTK7 protein depletion inhibits embryonic Wnt/β-catenin signaling by strongly decreasing LRP6 protein levels.^65^ These findings demonstrate the activating role of PTK7 in regulating Wnt/β-catenin signaling. Thus, further experiments will be needed to uncover the underlying molecular mechanism of PTK7 regulating Wnt/β-catenin signaling.

#### Muscle-specific kinase (MuSK)

MuSK, also known as *unplugged*, is required for neuromuscular junction (NMJ) formation by responding to a critical nerve-derived signal agrin.^69,70^ MuSK functions as a Wnt receptor and has an extracellular region that contains the CRD, which is homologous to the CRD of Fzd.^37^ Evidence demonstrates that Wnt11, Wnt4, and Wnt9a all play important roles in regulating NMJ formation through binding to MuSK via the CRD.^71–73^ Wnt11 interacts with MuSK to activate PCP signaling in regulating synapse formation during neuromuscular development.^71^ Wnt4 contributes to the formation of vertebrate NMJ by binding to MuSK and initiating an increase in associated MuSK phosphorylation level.^72^ Moreover, Wnt9a and Wnt11 induce acetylcholine receptor clustering in muscle cells by binding to MuSK and inducing MuSK dimerization along with tyrosine phosphorylation in an LRP4-dependent manner.^73^

### Wnt signaling pathway

Wnts can induce different signaling pathways by binding to different receptors. Generally, Wnt signaling is divided into two branches depending on the different requirements for β-catenin, a cytoplasmic adaptor protein with membrane and nuclear functions. The β-catenin-dependent pathway is also known as the canonical Wnt signaling pathway, otherwise, the non-canonical Wnt signaling pathway is independent of β-catenin transcriptional function.^74^ The canonical Wnt signaling pathway is also called the Wnt/β-catenin signaling pathway (Fig. 1a). For non-canonical Wnt signaling pathway, there are two major types: the Wnt/PCP pathway and Wnt/calcium (Ca^2+^) pathway, in which Wnts trigger signal transduction in different ways (Fig. 1b).

### Canonical Wnt signaling pathway (Wnt/β-catenin signaling pathway)

The canonical Wnt signaling pathway is well-known and extensively referred to as the Wnt/β-catenin signaling pathway. This pathway plays important and versatile roles from embryologic development to adult tissue homeostasis, and its aberrations cause numerous diseases.^1,2,19^ In the canonical Wnt signaling pathway, β-catenin functions as a key transcriptional co-activator and transmits extracellular signals to activate the target genes (Fig. 1a). Without Wnt, β-catenin is sequestered by a destruction complex composed of glycogen synthase kinase 3β (GSK3β), Axin, APC, and casein kinase 1 (CK1). This complex is then constitutively phosphorylated, after which it is degraded by ubiquitin-mediated proteolysis (Fig. 1a). However, the Wnt/β-catenin signaling pathway initiates with the binding of a Wnt to its receptor complex, including the seven-transmembrane receptor Fzd and the co-receptor LRP5 or LRP6. This binding of Wnt to its receptor complex further mobilize GSK3β and CK1 to the cell membrane, where they phosphorylate serines on Lrp5/6, promoting the formation of a signalosome, and the recruitment of Dvl, and Axin.^75,76^ This results in the release of β-catenin from the destruction complex and induces the accumulation of cytosolic β-catenin, which enters into the nucleus and binds to TCF/LEF to activate the target gene expression (Fig. 1a).

### Non-canonical Wnt signaling pathway

#### Wnt/planar cell polarity (PCP) signaling pathway

The Wnt/PCP signaling pathway is the most extensively studied of the non-canonical Wnt signaling pathways. PCP specifically refers to the organization of the epithelium orthogonally to the apicobasal polarity axis. The Wnt/PCP pathway mainly functions in regulating cell polarity in morphogenetic processes, such as coordinating cell polarity and morphology during the morphological polarization of hair follicles, pulmonary angiogenesis, morphogenetic movements, and the closure of caudal neural plate.^66,77–79^ The pathway also shows a key role in determining eventual cell fate.^80^ Wnt/PCP pathway can be activated by various Wnts, especially Wnt5a, Wnt7, and Wnt11. Fzds act as receptors in the Wnt/PCP pathway, with Fzd3, Fzd6, and Fzd7 favoring this signaling pathway. Instead of LRP5/6, the members of the receptor tyrosine kinase family (ROR2, RYK, and PTK7) and other membrane proteins (VANGL2, Glypican, Syndecan 4) are adopted as co-receptors in Wnt/PCP pathway.^52,66,81–83^ The binding of Wnts recruits Dvl to Fzds and activates the small GTPases Rac1 and RhoA, which in turn induce changes in the actin cytoskeleton, and activate JUN-N-terminal kinase (JNK) and RHO kinase (ROCK) to regulate downstream signals.^84^ (Fig. 1b).

#### Wnt/calcium (Ca^2+^) signaling pathway

Wnt/Ca^2+^ signaling pathway is another non-canonical (β-catenin-independent) Wnt signaling pathway that was initially identified in X. *laevis* and zebrafish.^85^ This pathway functions as a key mediator in development^86,87^ and is involved in physiological (e.g., hematopoiesis, neuronal excitability, neuron regeneration) and pathological (e.g., inflammation, neurodegeneration, and cancer) processes.^88–92^ In Wnt/Ca^2+^ signaling pathway, the binding of Wnts to Fzd mediates the activation of a G protein. In turn, the G protein activates phospholipase C (PLC), leading to the generation of inositol 1,4,5-triphosphate (IP3) and diacylglycerol (DAG) which increases intracellular Ca^2+^ concentration. Moreover, Wnt/Fzd additionally activates cGMP (cyclic guanosine monophosphate) - specific phosphodiesterase 6 (PDE6), resulting in a decrease of cellular cGMP and the inactivated protein kinase G (PKG), which in turn increases intracellular Ca^2+^ concentration. The Ca^2+^ activates calcium calmodulin-dependent protein kinase II (CaMKII), calcineurin, or protein kinase C (PKC), which further activates various transcription factors (NF-κB (nuclear factor κB), NFAT (nuclear factor associated with T cells) and CREB (cAMP-responsive element-binding protein)) that regulate downstream gene expression^90,93^ (Fig. 1b). Notably, the Wnt/Ca^2+^ signaling and Wnt/β-catenin are coupled in cells, which challenges the canonical and non-canonical categorization of Wnt signaling.^94,95^

For both the canonical and non-canonical Wnt signaling transduction, the Dvl proteins are involved in and regarded as the hub of Wnt signaling.^96^ In the canonical Wnt signaling pathway, Dvl is recruited by the receptor Fzd and prevents the phosphorylation and degradation of cytosolic β-catenin. In the non-canonical Wnt/PCP signaling pathway, Dvl functions via the DAAM-RhoA axis and the Rac1 axis. In the non-canonical Wnt/Ca^2+^ signaling pathway, Dvl signals through the PLC or PDE6 to induce the downstream Ca^2+^ signaling.

### Activators/agonists of Wnt signaling

**Fig. 2:**
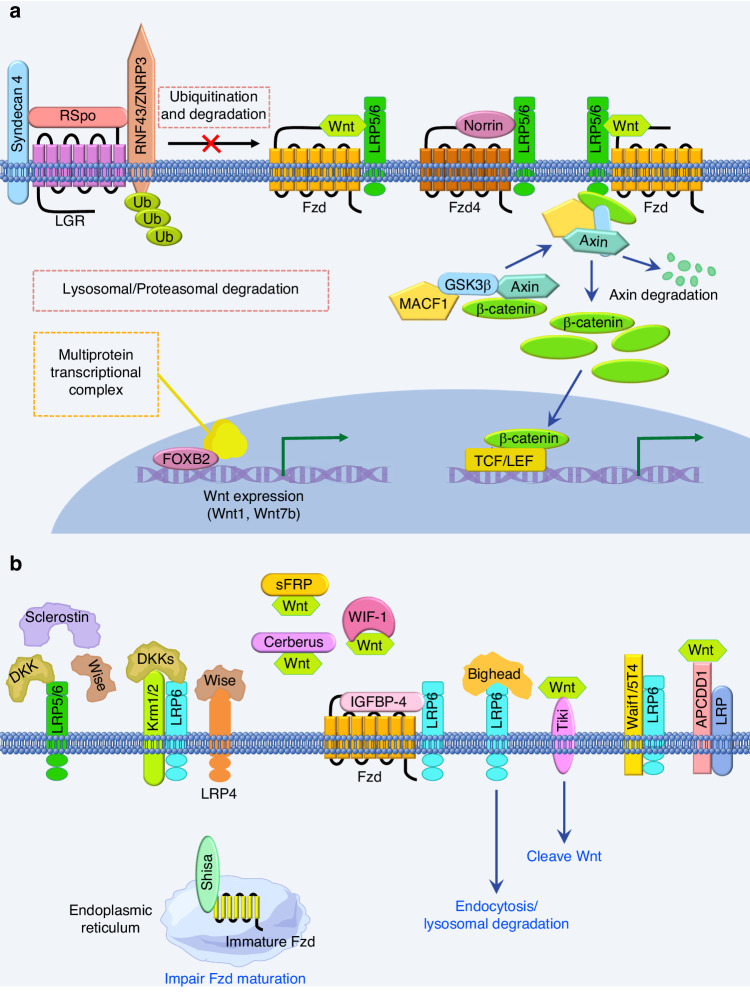
Activators/agonists and inhibitors/antagonists of Wnt signaling. **a** Activators/agonists of Wnt signaling. RSpo maintains the Wnt signal by binding to LGR and RNF43/ZNRF3 to prevent the polyubiquitination and endocytosis of Fzd induced by RNF43/ZNRF3. Norrin, acting as a mimic of Wnts, specifically binds to Fzd4 with high affinity and activates the Wnt/β-catenin signaling pathway in a LRP5/6-dependent manner. MACF1 promotes Wnt/β-catenin signaling by translocating the Axin complex (Axin, β-catenin, and GSK3β) from cytoplasm to cell membrane, where GSK3β is inactivated by phosphorylation and β-catenin is released and enters the nucleus to activate target genes. FOXB2 interacts with multiprotein transcriptional complex to induce multiple Wnt ligands, including Wnt1 and Wnt7b to increase TCF/LEF-dependent transcription. **b** Inhibitors/antagonists of Wnt signaling. Sclerostin, DKK and Wise bind to LRP5/6 to interfere with the binding between LRP5/6 and Fzd to inhibit Wnt signaling. Krm1 and Krm2 (Krm1/2) cooperate with Dkks to form a complex with LRP6 and inhibit the Wnt signaling. Wise also binds to LRP4 to inhibit Wnt/β-catenin signaling. sFRPs, WIF-1 and Cerberus inhibit Wnt signaling by interacting with Wnts. IGFBP-4 binds to both Fzd and LRP6 to antagonize Wnt signaling. Bighead, Tiki, Waif1/5T4, and APCDD1 prevent ligand–receptor interaction to antagonize Wnt signaling. Shisa impairs Fzd maturation to inhibit Wnt signaling

Besides Wnts, there are additional described proteins known to activate Wnt signaling, including R-spondin (RSpo) proteins^97^ and Norrin proteins,^98–100^ two different families of growth factors. In addition, microtubule actin crosslinking factor 1 (MACF1), a versatile spectraplakin^101^ and FOXB2, an uncharacterized forkhead box family transcription factor, are demonstrated as potent activators to promote Wnt signaling^102,103^ (Fig. 2a, Table 1).

**Table 1:** Activators/agonists and inhibitors/antagonists of Wnt signaling

Category	Name/Family Name	Type	Main Action Mechanism	Key Reference
Activators/agonists	RSpos	Secretory	1. Synergize with Wnts 2. Interfere DKK1 action 3. Interact with Fzd8 and LRP6 4. Bind to LGR4/5/6	^105–111^
Activators/agonists	Norrin	Secretory	Bind to Fzd4 to activate Wnt/β-catenin signaling pathway in a LRP5/6-dependent manner	^98,118–120^
Activators/agonists	MACF1	Non-secretory	Translocating the Axin complex (Axin, β-catenin, and GSK3β) from cytoplasm to cell membrane, facilitating β-catenin translocation into nucleus by phosphorylating GSK3β	^102,125^
Activators/agonists	FOXB2	Non-secretory	Induce multiple Wnt ligands to increase TCF/LEF-dependent transcription	^103^
Inhibitors/antagonists	DKK (Dkk1-Dkk4)	Secretory	1. Bind to the LRP5/6 to disrupt the Fzd-LRP5/6 complex formation 2. Bind to Krm1 and Krm2 to form complex with LRP6 to inhibit Wnt-Fzd-Lrp6 function	^129,143–145^
Inhibitors/antagonists	sFRPs (sFRP1- sFRP5)	Secretory	Bind to Wnts and prevent the interaction of Wnts and their receptors	^127,152^
Inhibitors/antagonists	WIF-1	Secretory	Bind to and sequester Wnts	^131,160^
Inhibitors/antagonists	Sclerostin	Secretory	Bind to Wnt co-receptors LRP5 and LRP6 and disrupt the formation of Wnt-receptor complex	^133,164,165^
Inhibitors/antagonists	Wise	Secretory	Bind to LRP5, LRP6, or LRP4	^132,168,169^
Inhibitors/antagonists	IGFBP-4	Secretory	Bind to both Fzd and LRP6	^134^
Inhibitors/antagonists	Cerberus	Secretory	Through proteolysis	^135^
Inhibitors/antagonists	Bighead	Secretory	Induce LRP6 endocytosis and lysosomal degradation	^136^
Inhibitors/antagonists	Shisa	Transmembrane	Physically interact with immature forms of Fzd within the ER to impair Fzd maturation	^137,178^
Inhibitors/antagonists	Tiki1	Transmembrane	Cleave eight amino-terminal residues of a Wnt and impair the receptor binding capacity of Wnt	^138^
Inhibitors/antagonists	Waif1/5T4	Transmembrane	1. Modify LRP6 subcellular localization 2. Structurally, Tyr325 plus the LRR1 surface centered on a second exposed aromatic residue, Phe97, are essential for inhibition of Wnt/β-catenin signaling	^139,180^
Inhibitors/antagonists	APCDD1	Transmembrane	Physically interact with Wnt3a and LRP5, and impair the formation of Wnt receptor complex	^140^

*APCDD1* adenomatosis polyposis coli down-regulated 1, *DKK* Dickkopf, *ER* endoplasmic reticulum, *Fzd* frizzled, *GSK3β* glycogen synthase kinase 3β, *IGFBP* insulin-like growth factor binding protein, *Krm* Kremen, *LGR* leucine-rich repeat-containing G-protein-coupled receptor, *LRP* low-density lipoprotein receptor-related protein, *LRR* leucine-rich repeats, *MACF1* microtubule actin crosslinking factor 1, *RSpo* R-spondin, *sFRP* secreted Frizzled related protein, *TCF/LEF* T-cell factor/lymphoid enhancer binding factor, *Waif1* Wnt-activated inhibitory factor 1, *WIF* Wnt inhibitory factor

### R-Spondin (RSpo)

The R-spondin (RSpo) is a family of cysteine-rich secretory proteins. There are four members, RSpo1, RSpo2, RSpo3, and RSpo4, who show an overall similarity of 40%–60% sequence homology and structural homologies.^104^ All four RSpos are composed of an N-terminal signal peptide, two furin-like CRDs, one thrombospondin type I domain, and a C-terminal basic acid-rich domain. All RSpos are demonstrated as activators of the Wnt signaling pathway, showing that RSpo2 and RSpo3 are more potent than RSpo1, while RSpo4 is relatively inactive.^105,106^ The RSpos synergize with Wnts and require the presence of Wnts and LRP6 to activate Wnt/β-catenin signal transduction.^105–108^ The activation effect of RSpos on Wnt/β-catenin signaling is implemented by interfering with Dickkopf1 (Dkk1)-mediated LRP6 and Kremen association.^105,106^ In addition, RSpo3 interacts with Fzd8 and LRP6 to enhance Wnt signaling.^107^ Recent evidence demonstrates that the leucine-rich repeat-containing G-protein-coupled receptor 4/5/6 (LGR4/5/6) functions as a receptor of RSpos to potentiate Wnt/β-catenin signaling.^109,110^ The RSpo/LGR5 complex functions by neutralizing ring finger 43 (Rnf43) and zinc and ring finger 3 (Znrf3), two transmembrane E3 ligases that function as negative feedback regulators of Wnt signaling by removing Wnt receptors Fzd and LRP6 on the cell surface.^111^ The interaction between RSpos and LGR4/5/6 is mediated by the furin-like CRD of RSpos.^110,112^ Besides the Wnt/β-catenin signaling pathway, RSpos also modulate the non-canonical Wnt signaling pathway. RSpo3 promotes the Wnt/PCP signaling pathway^113^ while RSpo1 inhibits the non-canonical Wnt7a/Fzd7/Rac1 signaling pathway.^114^ RSpo2 suppresses Wnt5a/Fzd7-driven non-canonical Wnt signaling pathway^115^ while RSpo3 activates the non-canonical Wnt/Ca^2+^/NFAT signaling pathway.^116^ These differences may be due to the different components of non-canonical signaling pathways.

### Norrin

Norrin is a small, highly conserved secreted signaling molecule that exhibits a cystine-knot motif and functions as an atypical Wnt ligand by forming complex with Fzd4 and LRP5/6.^100,117^ Norrin specifically binds to Fzd4 with high affinity and activates the Wnt/β-catenin signaling pathway in a LRP5/6-dependent manner.^98,118^ Although showing similarity with Wnt in activating the Wnt/β-catenin signaling pathway, the structure of Norrin is completely different from Wnt. The Norrin structure contains a cystine-knot motif and forms a homodimer via intermolecular disulfide bonds.^119^ Moreover, Norrin is not lipid-modified like Wnt. The crystal structure analysis of the Fzd4_CRD_-Norrin complex reveals the specific interaction between Norrin and Fzd4 via the CRD of Fzd4.^119^ More recently, Bang et al. evaluated the conformational change of Fzd4 upon Norrin binding and demonstrated that the linker domain (the region between CRD and transmembrane domain) of Fzd4 is responsible for its tight binding to Norrin rather than CRD.^120^ Therefore, Norrin functions as an activator of Wnt signaling through binding to Fzd4.

### Microtubule actin crosslinking factor 1 (MACF1)

MACF1 was first discovered as a member of the actin crosslinker superfamily and named actin crosslinking factor 7 (ACF7).^121^ Subsequent studies reveal the association of ACF7 with both actin and microtubules (MTs), thus renaming it MACF/MACF1.^122^ As a versatile spectraplakin, MACF1 is widely involved in multiple cellular processes (e.g., cell migration, proliferation, and differentiation), embryo development, tissue homeostasis, and disease.^101,123^ Given the similar phenotype of *MACF1*^*−/−*^ embryo and *Wnt3*^*−/−*^ and LRP5/6 double-knockout embryos,^47,124^ which lacks the primitive streak, node, and mesoderm, a relationship between MACF1 and Wnt signaling was indicated. Chen et al. firstly reported that the reduction of MACF1 resulted in the suppression of Wnt-induced TCF/β-catenin-dependent transcriptional activation, suggesting a positive role of MACF1 in regulating Wnt/β-catenin signaling.^102^ They demonstrated that MACF1 positively regulated the Wnt/β-catenin signaling by translocating the Axin complex (Axin, β-catenin, and GSK3β) from cytoplasm to cell membrane, where GSK3β was inactivated by phosphorylation and β-catenin was released and entered into the nucleus to activate target genes.^102^ Similarly, we found that MACF1 played a positive role in increasing β-catenin level in osteoblasts, facilitating β-catenin translocation into the nucleus and increasing its transcription activity by phosphorylating GSK3β.^125,126^ Therefore, MACF1 can be considered a novel activator for Wnt/β-catenin signaling.

### FOXB2

FOXB2, an uncharacterized forkhead-box family transcription factor, was recently identified as a potent activator of Wnt signaling in normal and cancer cells by Moparthi et al.^103^ They found that FOXB2 induced multiple Wnt ligands, including Wnt7b, to increase TCF/LEF-dependent transcription without activating Wnt co-receptor LRP6 or β-catenin. Several transcription regulators, including YY1, JUN, and DDX5, act as cofactors in FOXB2-dependent Wnt signaling. Moreover, Moparthi et al. found that FOXB2-controlled Wnt signaling was induced in the neuroendocrine differentiation of prostate cancer cells, implicating FOXB2 expression in advanced prostate cancer. Their findings suggest that FOXB2 is a tissue-specific Wnt activator.^103^

### Inhibitors/antagonists of Wnt signaling

There exists both secreted and transmembrane Wnt signaling inhibitors/antagonists^127^ (Fig. 2b, Table 1). The secreted inhibitors mainly contain six families, including the Dkk family of proteins,^128,129^ secreted Frizzled related proteins (sFRPs),^130^ Wnt inhibitory factor 1 (WIF-1),^131^ Wise, Sclerostin (SOST),^132,133^ insulin-like growth factor binding protein 4 (IGFBP-4),^134^ and Cerberus.^135^ Additionally, Bighead, a secreted protein, was identified as a novel Wnt inhibitor.^136^ The transmembrane inhibitors mainly contain Shisa proteins,^137^ Tiki1,^138^ Wnt-activated inhibitory factor 1 (Waif1/5T4),^139^ and adenomatosis polyposis coli down-regulated 1 (APCDD1).^140^ These factors antagonize Wnt signaling by preventing ligand-receptor interactions or Wnt receptor maturation (Fig. 2b, Table 1).

### Secreted inhibitors/antagonists of Wnt signaling

#### Dickkopf (Dkk) family

The Dkk family of cysteine-rich secretory proteins are well-characterized inhibitors for Wnt signaling. Since the discovery of Dkk1,^141^ 4 main members (Dkk1, Dkk2, Dkk3, and Dkk4) were identified in the Dkk family.^142^ They specifically inhibit the Wnt/β-catenin signaling pathway. Dkk1, Dkk2, and Dkk4 bind to LRP5/6 to disrupt the Fzd-LRP5/6 complex formation, thus inhibiting Wnt signaling,^129,143,144^ while Dkk3 does not bind to LRP5 or LRP6 and does not affect Wnt signaling.^129^ Besides LRP5/6, Dkks also bind to Kremen (Krm) proteins (Krm1 and Krm2), which are single-pass transmembrane receptors. Krms greatly enhance the inhibitory ability of Dkks on Wnt signaling.^145^ Krm1 and Krm2 cooperate with Dkk1 to form a complex with LRP6 and inhibit the Wnt-Fzd-LRP6 function.^145^ However, the study of *Krm1*^−/−^/*Krm2*^−/−^ double-mutant mice demonstrates that Krms is not completely required for Dkk1 function,^146^ suggesting that the inhibitory effect of Dkk1 on Wnt-LRP6 interaction may be sufficient to suppress Wnt signaling. Notably, Dkk2 acts not only as an inhibitor but also as an activator for Wnt signaling depending on the cellular context. In *Xenopus* embryos, Dkk2 synergizes with Fzd receptor^147^ or interacts with LRP6^148^ to activate rather than inhibit the Wnt/β-catenin signaling pathway. However, Dkk2 inhibits the Wnt/β-catenin signaling pathway in HEK293T cells.^147^ This dual role of Dkk2 may be modulated by the Krm2, which converts Dkk2 from an agonist to an antagonist of LRP6.^149^

#### Secreted Frizzled-related proteins (sFRPs)

The sFRPs, the largest family of Wnt inhibitors, resemble the ligand-binding CRD found in the Fzds of Wnt receptors.^150^ The first sFRP member, Fzdb (Frizzled motif associated with bone development), was discovered as a chondrogenic factor^151^ and was subsequently shown as a Wnt antagonist.^152^ Subsequently, other sFRPs were identified.^153^ In humans, there are five members in the sFRP family, including sFRP1, sFRP2, sFRP3, sFRP4, and sFRP5.^150^ Sharing sequence similarity with CRDs of Fzds, sFRPs directly bind to Wnts, preventing the interaction of Wnts and their receptors, and thus inhibiting Wnt signaling.^127,152^ sFRPs are demonstrated to inhibit both canonical Wnt signaling and non-canonical Wnt/PCP signaling.^154,155^ However, Holly et al. demonstrated that sFRP proteins functioned as facilitators of Wnt signaling within the dorsal retina.^156^ Further investigation of sFRP1 by Xavier et al. showed that sFRP1 either inhibited or enhanced Wnt3a/β-catenin signaling, depending on its concentration and the specific cellular context.^157^

#### Wnt inhibitory factor 1 (WIF-1)

Similar to sFRPs, WIF-1 is a secreted inhibitor for Wnt signaling by directly binding to and sequestering Wnts.^131^ WIF-1 is composed of an N-terminal secretion signal sequence, a unique and highly conserved WIF domain, five epidermal growth factor (EGF)-like repeats, and a hydrophilic C-terminal domain. The WIF domain is responsible for the binding of WIF-1 to Wnts. Moreover, the crystal structure analysis of human WIF-1 in combination with biophysical and cellular assays reveals that Wnts bind to both the WIF domain and the EGF-like domains of WIF-1.^131^ By binding to both types of Wnts and sequestering them, WIF-1 suppresses both canonical and non-canonical Wnt signaling.^158–160^

#### Sclerostin (SOST)

Sclerostin is the product of the *Sost/SOST* gene that is localized to human chromosome region 17q12-q21.^161^ It is an osteocyte-expressed glycoprotein.^162^ Sclerostin was first considered as an antagonist of bone morphogenetic protein (BMP) signaling due to its competition with type I and type II BMP receptors for binding to BMPs and decreased BMP signaling.^163^ However, subsequent studies demonstrate that it is also an antagonist/inhibitor of Wnt signaling by binding to Wnt co-receptors LRP5 and LRP6, thereby disrupting the formation of the Wnt-receptor complex.^133,164,165^ In addition, LRP4, another LRP family member, facilitates the inhibitory action of sclerostin on Wnt signaling.^166^ Evidence shows that the suppressive action of sclerostin on Wnt signaling transduction occurs in both osteoblasts and osteocytes in both paracrine and autocrine manner.^167^

#### Wise

Wise, also refered as sclerostin domain containing 1, Ectodin, and uterine sensitization-associated gene-1, is a secreted factor that was identified by a functional screen for novel factors with the potential to alter the anteroposterior character of neutralized *Xenopus* animal caps.^132^ In *Xenopus*, Wise either inhibits or activates Wnt signaling in different assays, suggesting it as a context-dependent regulator of Wnt signaling.^132^ By sharing 38% amino acid identity with sclerostin, Wise also inhibits Wnt signaling by binding to LRP5 or LRP6.^168^ Moreover, Wise binds to LRP4 to inhibit Wnt/β-catenin signaling.^169^

#### IGFBP-4

IGFBP-4 is a member of the family of IGFBPs that regulate numerous cellular processes by modulating the actions of insulin-like growth factors.^170^ IGFBP-4 was identified as an inhibitor of canonical Wnt signaling required for cardiogenesis.^134^ It inhibits Wnt signaling by binding to both Fzd and LRP6.^134^ Interestingly, both IGFBP-4 and Dkk1 are inhibitors of canonical Wnt signaling and are crucial for heart development, but they play opposing roles in cardiac ischemia by differentially targeting LRP5/6 and β-catenin.^171^ IGFBP-4 protects the ischemic heart by inhibiting β-catenin while Dkk1 enhances the injury response by inducing LRP5/6 endocytosis and degradation.^171^ Moreover, IGFBP-4 activates canonical Wnt signaling in human renal cell carcinoma.^172^ These different findings on the role of IGFBP-4 in modulating Wnt signaling may be dependent on the cellular context.

#### Cerberus

Cerberus was discovered in *Xenopus* as a head-inducing secreted factor that is expressed in the anterior endoderm of Spemann’s organizer^173^ and was identified as a multifunctional inhibitor of Nodal, BMP, and Wnt signaling.^135,174^ Subsequently, Cerberus-like proteins were identified in other vertebrates (e.g., mouse, chick, zebrafish) and grouped in the Cerberus/Dan family, showing key roles in the regulation and generation of asymmetries in the early embryo.^175^ Furthermore, Cerberus also contains a cystine-knot domain. However, proteolytically processed isoforms of *Xenopus* Cerberus that still contain the cystine-knot domain cannot bind to Wnt8, suggesting that the inhibitory ability of Cerberus on Wnt signaling might be regulated by proteolysis.^135^

#### Bighead

Like Cerberus, Bighead was also screened in the Spemann organizer as a secreted protein and identified as a novel inhibitor of Wnt signaling by causing LRP6 endocytosis and lysosomal degradation.^136^ Bighead overexpression within embryos leads to the development of larger fetal heads, while its deficiency reduces head development by regulating Wnt signaling.^136,176^ As a novel Wnt inhibitor, the role of Bighead in modulating Wnt signaling needs further investigation.

### Transmembrane inhibitors/antagonists of Wnt signaling

#### Shisa proteins

Shisa proteins compose a big family that consists of nine subfamilies in vertebrates at present.^177^ Shisa proteins are characterized by an N-terminal cysteine-rich domain and a proline-rich C-terminal region and are a novel family of modulators of both Wnt and FGF signaling. *Xenopus* Shisa (*Xenopus* Shisa 1), the founding member of the Shisa family, was first identified as a novel antagonist of Wnt signaling for head formation by Yamamoto et al.^137^ Thereafter, *Xenopus* Shisa 2 was demonstrated to inhibit Wnt signaling.^178^ Moreover, mShisa, a mouse homolog of *Xenopus* Shisa 1, also antagonizes Wnt signaling.^179^ Shisa proteins inhibit Wnt signaling by physically interacting with immature forms of Fzd within the endoplasmic reticulum to impair Fzd maturation.^137,178^

#### Tiki1

Tiki1 is another transmembrane Wnt antagonist that is identified by functional cDNA screening as a Spemann-Mangold Organizer-specific gene required for anterior development.^138^ It antagonizes Wnt function by acting as a protease to cleave eight amino-terminal residues of a Wnt, leading to oxidized Wnt oligomers that exhibit impaired receptor-binding capability.^138^

#### Wnt-activated inhibitory factor 1 (Waif1/5T4)

Waif1/5T4 is a single-pass transmembrane protein with eight leucine-rich repeats (LRRs) in the extracellular region and *Waif1a* was recently identified as a transcriptional target of Wnt/β-catenin signaling in zebrafish embryos.^139^ Moreover, Waif1 acts as an antagonist of Wnt8-mediated β-catenin signaling by controlling LRP6 availability, while activating non-canonical Wnt/PCP Wnt signaling through enhancing a non-canonical function of DKK1.^139^ Zhao et al. identified the crystal structures of the extracellular domain of Waif1/5T4, which reveal a highly glycosylated rigid core containing eight LRRs.^180^ Besides, they suggested that Tyr325 plus the LRR1 surface centered on a second exposed aromatic residue, Phe97, are essential for the inhibition of Wnt/β-catenin signaling.^180^

#### Adenomatosis polyposis coli down-regulated 1 (APCDD1)

APCDD1 is a novel inhibitor of Wnt signaling identified by Shimomura et al. when studying hereditary hypotrichosis simplex.^140^ It is a membrane-bound glycoprotein and is abundant in human hair follicles. APCDD1 is shown to inhibit Wnt signaling by physically interacting with Wnt3a and LRP5, which impairs the formation of the Wnt receptor complex.^140^ Being broadly expressed in various tissues and cell types, APCDD1 plays important roles in other Wnt-regulated biological processes, and further coordinates vascular pruning and barrier maturation by precisely modulating Wnt/Norrin signaling activity.^181^ Moreover, APCDD1 promotes adipogenic differentiation by inhibiting Wnt signaling^182^ but maintains the expression and activation of β-catenin during the osteogenic differentiation of human dental follicle cells.^183^ These contrary findings suggest that APCDD1 may regulate Wnt signaling depending on the cellular context.

### Interaction of the Wnt signaling pathway with other signaling pathways

**Fig. 3:**
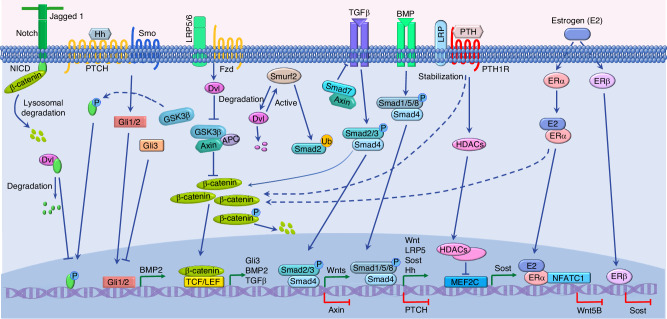
The interaction of Wnt signaling pathway with other signaling pathways. Wnt signaling interacts with Notch signaling pathway. β-catenin, the key component of Wnt signaling, activates Notch signaling by targeting Jagged 1 to activate Notch signaling. GSK3β also activates Notch signaling by phosphorating NICD. However, Dvl inhibits Notch signaling by inducing NICD degradation. In contrast, Notch negatively regulates β-catenin stability by inducing its lysosomal degradation. Wnt signaling inhibits Hedgehog signaling by regulating the expression of Gli3, the main repressor of Hedgehog signaling. TGFβ/BMP signaling and Wnt signaling determine the expression of ligand and components (e.g., Wnts, LRP5, Sost, Axin, BMP2, and TGFβ) of each other and the interaction between Smad7 and Axin links these two signaling pathways. Moreover, Dvl is targeted for degradation by Smurf2, a regulator of TGF-β/BMP signaling pathway. Conversely, Dvl activates Smurf2. PTH signal stabilizes the β-catenin to activate Wnt/β-catenin signaling. In addition, PTH inhibits sclerostin expression by promoting nuclear accumulation of HDACs to repress MEF2C-dependent *Sost* enhancer. Estrogen signaling interacts with Wnt signaling. The estrogen 17β-Estradiol (E2) activates estrogen signaling by binding to ERα to suppress the expression of WNT5B, but to increase the expression and activation levels of β-catenin. Besides, ERβ mediates the E2 suppression on the expression of Sost, an antagonist of Wnt signaling

As a versatile signaling pathway, Wnt signaling pathway interacts with multiple other signaling pathways such as Notch, Hedgehog, transforming growth factor β (TGF-β)/BMP, parathyroid hormone (PTH), and estrogen signaling pathways (Fig. 3).

#### Wnt signaling and Notch signaling

The Notch signaling pathway is a highly conserved pathway that is important in controlling cell function and tissue homeostasis. Both Notch signaling and Wnt signaling are found in all multicellular animals and they represent two major pathways in controlling cell behavior during development. Therefore, a strong interaction between Wnt signaling and Notch signaling is supposed. The interaction between these two signaling pathways was initially reported in the context of development.^184^ Furthermore, increasing evidence demonstrates the strong interaction between Wnt and Notch signaling pathways.^185,186^ β-catenin activates Notch signaling by targeting Jagged 1, the Notch ligand, indicating that the Notch pathway is downstream of the Wnt/β-catenin pathway.^187,188^ In contrast, Notch negatively regulates β-catenin stability.^189^ Moreover, components of Wnt signaling including GSK3β and Dvl play key roles in Notch signaling by modulating the Notch intracellular domain.^185^

#### Wnt signaling and Hedgehog signaling

The Hedgehog signaling pathway shows critical roles in both physiological and pathological processes.^190^ There are three vertebrate Hedgehog homologs, including Sonic Hedgehog (Shh), Desert Hedgehog (Dhh), and Indian Hedgehog (Ihh). Shh mainly functions in neuronal development,^191^ Dhh exerts its main role in the gonads,^192^ while Ihh is important for skeletal development.^193^ Similar to Wnt signaling, Hedgehog signaling also plays a key role throughout embryonic development. Therefore, the interaction between Hedgehog and Wnt signaling pathways shows profound physiological effects, such as regulating embryogenesis, tumorigenicity, and blood-brain barrier development.^194,195^ sFRP1, an inhibitor of Wnt signaling, is an important cross-point between Wnt signaling and Hedgehog signaling.^194^ Hedgehog signaling negatively regulates Wnt signaling by activating sFRP1 expression, while Wnt signaling inhibits Hedgehog signaling through regulating the expression of glioma-associated oncogene homolog 3 (Gli3), the main repressor of Hedgehog signaling.^194^

#### Wnt signaling and TGF-β/BMP signaling

The TGF-β superfamily is composed of more than 40 members, including TGF-βs (TGF-β1, TGF-β2, and TGF-β3), BMPs (14 BMPs), and activins and growth differentiation factors.^196^ These members are involved in two main pathways, the TGF-β signaling pathway, and the BMP signaling pathway. Like Wnt signaling, TGF-β signaling also regulates cell fate and proliferation during development and tissue maintenance. Therefore, the interaction between the TGF-β/BMP and Wnt pathways is the focus of many studies. Throughout an animal’s lifespan, the two pathways are interconnected, and they interact at many stages of the signal transduction pathway, including at the extracellular, cytoplasmic, and nuclear levels.^197^ Extracellularly, TGF-β/BMP and Wnt regulate the production of their respective ligands in a reciprocal manner. In the cytoplasm, there are interactions between the components of these signaling pathways, such as Dvl-1 and Smad1 interaction.^198^ Moreover, Dvl is targeted for degradation by Smurf2 (SMAD ubiquitination regulatory factor 2), which is a regulator of TGF-β/BMP signaling pathway. Conversely, Dvl activates Smurf2 to allow Smurf2 ubiquitinate the substrates from Wnt/PCP pathway and TGF-β/BMP pathway.^199^ In the nucleus, these signaling pathways interact to regulate a variety of shared target genes synergistically.^197^ Numerous studies demonstrate the interaction between TGF-β/BMP signaling and Wnt signaling in patterning the mesoderm, cell differentiation, and tissue development.^200–202^

#### Wnt signaling and PTH signaling

PTH (parathyroid hormone) is an 84-amino-acid polypeptide hormone and is essential in regulating calcium homeostasis. As a major regulator of bone remodeling, PTH interacts with the Wnt signaling pathway.^203^ Studies reveal that PTH induces osteoblast differentiation by regulating Wnt/β-catenin signaling, while Wnt/β-catenin signaling regulates chondrocyte differentiation via PTH.^204^ PTH shows increased effects on the expression of Wnts and decreased effects on inhibitors of Wnt signaling, such as sclerostin, DKK1, and sFRP1.^205,206^ Evidences demonstrate that PTH inhibits sclerostin expression in osteocytes by promoting nuclear accumulation of histone deacetylases to repress myocyte enhancer factor 2 type C (MEF2C)-dependent *Sost* enhancer.^207–209^ Furthermore, Li et al. showed that LRP6 was required for PTH suppression of *Sost* expression through MEF2C.^210^ Moreover, PTH exhibits a regulatory effect on the expression of Wnt signaling components, such as LRP5, LRP6, FZD-1, β-catenin, and TCF/LEF.^211^ Furthermore, Wnt/β-catenin signaling also exhibits a regulatory effect on PTH signaling. Wnt/β-catenin signaling inhibits parathyroid hormone-related protein (PTHrP) signaling activity.^212^

#### Wnt signaling and estrogen signaling

Estrogens are the main female sex steroids that control many cellular processes, such as cell proliferation and differentiation. Estrogens exert their biological actions by binding to one of two specific estrogen receptors (ERs) ERα and ERβ.^213^ As a key regulator of bone mass, estrogen deficiency is one main cause of osteoporosis. Evidence indicates that estrogen levels are inversely associated with the production of sclerostin, an antagonist of Wnt signaling, suggesting the interaction between Wnt signaling and estrogen signaling.^214^ Kim et al. found that estrogen 17β-Estradiol (E2) suppressed the *SOST* expression induced by BMP2, but increases the expression and activation levels of β-catenin in osteoblasts.^215^ ERα antagonist abolishes the effect of E2 on *SOST* expression, demonstrating that estrogen signaling in osteoblasts negatively regulates *SOST* expression.^215^ In addition, ERα is requisite for the effectiveness of Wnt/β-catenin signaling contributing to bone cell early response to mechanical strain.^216^ ER and Wnt signaling interacts to regulate bone mass adaption in response to mechanical loading.^217,218^ However, Galea et al. reported that the inhibitory effect of estrogen signaling on Sost expression in osteoblasts was mediated by ERβ but not ERα.^219^ This contrary result with Kim’s finding may be due to the different cell type adopted in experiments. Recently, Suthon et al. found that 17β-Estradiol (E2) suppressed WNT5B expression through its receptor ERα binding at the enhancer containing single-nucleotide polymorphism (SNP) rs2887571.^220^ As WNT5B suppresses osteoblast differentiation via ROR1/2, which inhibits β-catenin activity, the above findings demonstrate that estrogen promotes osteoblast differentiation by activating Wnt/β-catenin signaling.^220^

## Wnt signaling in bone formation and homeostasis

Bone is a rigid organ that provides support and physical protection to various organs, and stores minerals for the body. Bone is formed through two major ways, either intramembranous ossification or endochondral ossification.^221^ Intramembranous ossification is responsible for the formation of flat bone and is initiated by the condensation of mesenchymal stem cells (MSCs). The MSCs differentiate into osteoblasts that secrete osteoid matrix and further differentiate into osteocytes.^221^ Endochondral ossification occurs in the formation of long bone and begins with the MSCs condensation. As opposed to intramembranous ossification, endochondral ossification begins with a deposited cartilaginous template that is later replaced by bone formation.^222^ In adult bone, bone homeostasis is maintained by the intricate balancing of bone remodeling, bone formation conducted by osteoblasts, and bone resorption conducted by osteoclasts. Disruption of this balance results in bone diseases, such as osteoporosis and osteopetrosis.

**Fig. 4:**
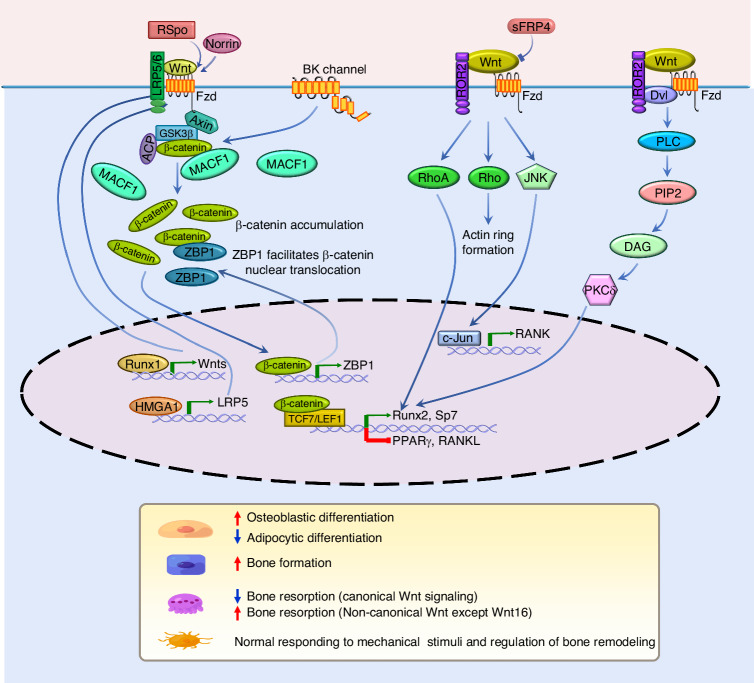
Schematic representation of Wnt signaling modulates bone homeostasis. Wnt signaling regulates bone homeostasis by modulating the biological function of bone cells, including BM-MSCs, osteoblasts, osteoclasts, and osteocytes. The canonical Wnt signaling promotes bone formation, inhibits bone resorption and adipocyte differentiation during maintaining bone homeostasis. When canonical Wnt signaling is activated by Wnts binding to the receptors or by the activators of Wnt signaling (e.g., RSpo, Norrin, and MACF1), β-catenin accumulates in the cytoplasm and translocates into the nucleus to regulate the target gene expression in bone cells to control bone cell capacity. Runx1 activates Wnt signaling by increasing Wnts expression to promote osteoblast differentiation. ZBP1 facilitates β-catenin nuclear translocation to promote Wnt signaling, while β-catenin in turn induces ZBP1 expression. HMGA1 transcriptionally regulates LRP5 expression to activate Wnt signaling. The non-canonical Wnt signaling promotes bone formation and bone resorption and inhibits adipocyte differentiation. Non-canonical Wnt5a signals through ROR2 to activate RhoA that is necessary and sufficient for osteogenic differentiation. Wnts also promote osteoblast differentiation and bone formation via PLC/PKCδ signaling. Wnt5a-ROR2 signals increase the expression of RANK by activating c-Jun to enhance RANKL-induced osteoclastogenesis and also promote actin ring formation via Rho to increase bone resorption. Besides, sFRP4, a Wnt inhibitor, dramatically suppresses the osteoclast differentiation by inhibiting non-canonical Wnt/ROR2/JNK signaling

The first connection between the Wnt signaling pathway and skeletal development was demonstrated in 1994 when Takada et al. found that *Wnt3a*-deficient mouse embryos exhibited axial defects.^223^ Additionally, studies of various mouse models reveal that abnormality of the components within Wnt signaling causes bone defects (reviewed by refs.,^204,224^ Table 2). Wnt signaling regulates bone development and maintains bone homeostasis by regulating the functions of bone cells, mainly including bone marrow mesenchymal stem cells (BM-MSCs), osteoblasts, osteoclasts, and osteocytes (Fig. 4).

**Table 2:** Bone phenotypes in mouse model carrying genetic modifications of Wnt signaling components

Gene	Loss or Gain of Function/Method/Cre line	Phenotype	Key Reference
Functional Group: Wnt ligands			
*Wnt3*	Loss of function/cKO/RARβ-Cre or Msx2-Cre	*Wnt3*^*n/c*^*; RARCre* mutant mice display defects only in the forelimb (RARβ-Cre) with variable severity. *Wnt3*^*n/c*^*; Msx2Cre* mutant mice exhibit defective hindlimbs with variable severity.	^453^
*Wnt3a*	Loss of function/KO/Germline	Homozygotes show absent somites for forelimb at 9.5 dpc and show embryonic lethality between 10.5 and 12.5 dpc (days postcoitum).	^223^
*Wnt4*	Loss of function/KO/Germline	Enhance synovial chondroid metaplasia in some joints with concomitant loss of Wnt9a. Suppress chondrogenic potential.	^454^
*Wnt4*	Gain of function/Transgene/Col2α1-Cre	Dwarfism with decreased bone formation, increased hypertrophic chondrocytes, and normal BMD.	^455^
*Wnt5a*	Loss of function/KO/Germline	Homozygotes show perinatal lethality. Truncation of the proximal skeleton and absence of distal digits. Delayed chondrocyte hypertrophy and skeletal ossification. Delayed osteoblast differentiation.	^456,457^
*Wnt5a*	Gain of function/Transgene/Col2α1 transgenic vector	Severe skeletal defects. Short skeletal elements in the limb and delayed ossification. Thick cartilage and delayed chondrocyte hypertrophy. Delayed chondrocyte differentiation and proliferation.	^457^
*Wnt5b*	Gain of function/Transgene/Col2α1 transgenic vector	Similar phenotype with Wnt5a (Col2α1) transgenic mice. Delayed chondrocyte hypertrophy and reduced bone ossification. Open skull. Delayed chondrocyte differentiation, but increased chondrocyte proliferation.	^457^
*Wnt7a*	Loss of function/KO/Germline	Loss of posterior skeletal elements in mutant limbs.	^458^
*Wnt7b*	Loss of function/cKO/Dermo-Cre	Wnt7b mutant mice were viable. Bone development defects. Diminution in ossification. Less bone in mouse embryos. Delayed maturation of chondrocyte and osteoblast differentiation.	^233^
*Wnt8*	Gain of function/Transgene/human β-actin promoter	Duplicated axes or a severely dorsalised phenotype.	^459^
*Wnt9a/Wnt14*	Loss of function/KO or cKO/Germline or Prx1-Cre	Homozygotes die at birth displaying partial joint fusions of carpal and tarsal elements and chondroid metaplasia in synovial and fibrous joints. Reduction of the length of appendicular long bones and the size of the mineralized regions. Ectopic cartilage nodules present within the midline sutures. Fusions of major joints.	^454^
*Wnt9a/Wnt14*	Gain of function/Transgene/Col2αl promoter/enhancer	Homozygotes die around 16.5 dpc. Short limbs. Reduced cartilage formation and endochondral ossification. Fused joints.	^460^
*Wnt9b*	Loss of function/cKO/CMV-Cre	Homozygotes die within 24 h of birth. No obvious difference in the dimension of the skull. Developmental defects of the upper jaw skeleton.	^461,462^
*Wnt10b*	Loss of function/KO/Germline	Decreased trabecular bone mass and serum osteocalcin. Age-dependent loss of bone mass. Osteopenia and reduction of osteoprogenitors. Reduced bone formation.	^239,241,463^
*Wnt10b*	Gain of function/Transgene/osteocalcin promoter	Increased mandibular bone and impaired eruption of incisors during postnatal development. High bone mass. Increased bone formation caused by increases in osteoblast number per bone surface, rate of mineral apposition, and percent mineralizing surface.	^463^
*Wnt10b*	Gain of function/Transgene/FABP4-promoter	Increased bone mass and strength. Resistance to the loss of bone that occurs with aging or estrogen deficiency.	^239^
*Wnt16*	Loss of function/KO/Germline	Thinner bone cortices, reduced bone strength and increase risk of fracture.	^338^
Functional Group: Wnt receptors			
*Fzd8*	Loss of function/KO/Germline	Osteopenia with normal bone formation and increased osteoclastogenesis. Reduction of the trabecular bone volume.	^321^
*Fzd9*	Loss of function/KO/Germline	Osteopenia caused by decreased bone formation. Low trabecular number. Normal osteoclast activity.	^258^
*Lrp4*	Loss of function/KO/Germline	Penetrant polysyndactyly in fore and hind limbs, and partially penetrant abnormalities of tooth development. Fused digital cartilage. Shortened total femur length, reduced cortical femoral perimeter, and reduced total femur BMC and BMD. Reduced Lumbar spine trabecular BV/TV. Increased serum and urinary bone turnover markers ALP, osteocalcin and desoxypyridinoline.	^264,464^
*Lrp5*	Loss of function/KO/Germline	Low bone mass. Decreased BMD. Both heterozygotes and homozygotes display limb defects. Decreased cancellous and cortical bone mass. Low cancellous bone volume in the distal femur and the lumbar vertebra. Decrease in both osteoblast surface and osteoclast surface. Kato et al. reported that there is no change in the number of osteoclast and chondrogenesis. Kato et al. reported decreased osteoblast proliferation while Yadav et al. reported normal osteoblast proliferation ex vivo.	^261,432,465–467^
*Lrp5*	Loss of function/cKO/CMV-Cre	Low bone mass postnatally.	^468^
*Lrp5*	Loss of function/cKO/Col1α1-Cre or Villin-Cre	Normal bone mass with Col1a1-Cre. Decreased bone mass in Villin-Cre due to a decrease in osteoblast numbers and bone formation. No change in osteoclast number.	^467^
*Lrp5*	Loss of function/cKO/DMP1-Cre or Villin-Cre	Decreased bone mass with Dmp1-Cre. Normal bone mass with Villin-Cre.	^263^
*Lrp5*	Gain of function/G171V, A214V	Increased bone mass, bone strength, and bone formation. Increased mechanical properties of tibiae in Lrp5 A214V mice but not in G171V mice.	^263,469^
*Lrp6*	Loss of function/GT1.8TM or cKO/Germline or CMV-Cre	Heterozygotes display limb defects. Homozygotes die at birth. Truncation of the axial skeleton. Limb defects.	^45,465,468^
*Lrp6*	Loss of function/cKO/Dermo1-Cre	Normal skeleton. Only a slight delay in ossification of the skull at E17.5. Mice die shortly after birth with concomitant loss of Lrp5, exhibiting misshaped skull and limbs, shortening of all skeletal elements, profound defect in the ossification of the craniofacial, the axial and the appendicular skeleton, extra cartilage elements.	^468^
*Lrp6*	Loss of function/Point mutation, R886W	Dysmorphologies of the axial skeleton and digits. Delayed ossification at birth and osteoporosis in adult. Decreased bone density. Reduced bone thickness.	^470^
Functional Group: Wnt antagonist			
*Dkk1*	Loss of function/KO/Germline	Homozygotes die at birth. Absence of skull derivatives anterior of the parietal bone, including nasal, mandibular, and maxillary bones. Duplications and fusions of limb digits. Heterozygotes display an increase in all bone formation parameters, with no change in bone resorption. Significant increase of the number of osteoblasts, mineral apposition, and bone formation rate. High bone mass.	^283,471^
*Dkk1*	Gain of function/Overexpression/Adenoviral vector encoding full-length chick *Dkk1*	Deletion of distal limb tissue. Reduced limb bud. Truncation of limbs and lack of the medial and distal limb elements in both fore- and hindlimbs.	^471^
*Dkk1*	Gain of function/Overexpression/Retroviral expression of Dkk1 in primary calvaria cells in vitro	Complete inhibition of osteoblast differentiation and formation of mineralized nodules and decrease in the ALP expression.	^283^
*Dkk1*	Gain of function/Transgene/3.6 kb Col1A1 promoter, 2.3 kb Col1A1 promoter	Transgenic mice constructed by 3.6 kb Col1A1 promoter show osteopenia with forelimb deformities. Transgenic mice constructed by 2.3 kb Col1A1 promoter show severe osteopenia without limb defects. Decreased bone mass.	^282^
*Dkk1*	Gain of function/Transgene/2.3 kb ColIα1 promoter	Reduced bone mass, bone formation and trabecular bone volume. Reductions in osteoblast surface per bone surface and in the number of osteoblasts per total bone area. Normal osteoclast surface per bone surface and the number of osteoclasts per total bone area.	^472^
*Dkk1*	Gain of function/Transgene/Col2α1 promoter and enhancer, Tie2 promoter and enhancer, Col10α1 promoter and enhancer	Chondrocyte-specific (Col2α1) and hypertrophic chondrocytes-specific (Col10α1) Dkk1 transgenic mice show normal cartilage and bone development. Endothelial cell-specific (Tie2) Dkk1 transgenic mice show defects in endochondral ossification and reduced skeletal length, but no defects in cartilage development. Endothelial cell-specific (Tie2) Dkk1 transgenic mice also show reduced total trabecular area, reduced trabecular thickness, increased trabecular number, and increase in the hypertrophic zone.	^473^
*Dkk2*	Loss of function/KO/Germline	Osteopenia. Defects in mineralization. Increased numbers of osteoids. Increase in the number of osteoclasts.	^285^
*Dkk2*	Gain of function/Transgene/Col2α1 promoter and enhancer, Tie2 promoter and enhancer	Normal cartilage, bone development, bone length and mineralization.	^473^
*Sfrp1*	Loss of function/KO/Germline	Increase trabecular bone mineral density, volume, and mineral apposition rate. Reduced osteoblast and osteocyte apoptosis. No change of bone resorption in vivo.	^278^
*Sfrp2*	Loss of function/KO/Germline	Brachydactyly, mild mesomelic shortening and posterior soft-tissue syndactyly. Decreased chondrocyte proliferation and delayed differentiation in distal limb chondrogenic elements.	^280^
*Sfrp3/Frzb*	Loss of function/cKO/EIIa-Cre	Increased articular cartilage loss during arthritis. Stiff bone due to increased cortical bone thickness and density. Increased periosteal anabolic response to mechanical loading.	^474^
*Sfrp4*	Gain of function/Transgene/2.3 kb Col1α1 promoter	Reduction of trabecular bone mass. Decreases in both osteoblast numbers and bone formation rate.	^281^
*Sfrp4*	Gain of function/Transgene/SAP promoter	No change of BMD at 5 weeks of age. Decreased gain of BMD with advancing age. Low trabecular BV/TV and Tb.Th.	^475^
*Sost*	Loss of function/KO/Germline	High bone mass characterized by marked increases in BMD, bone volume, bone formation, and bone strength. Significantly increased cortical bone. Enhanced trabecular bone architectural properties. Increased parietal thickness. Increased mechanical properties.	^290,469^
*Sost*	Gain of function/Transgene/Osteocalcin promoter	Osteopenia. Low bone mass. Disorganized bone architecture, thin cortices, reduced trabecular bone, and chondrodysplasia. Decreased bone strength. Reduction in osteoblast activity and bone formation. No significant change in bone resorption.	^163^
Functional Group: Effectors in cytoplasm			
*GSK3β*	Loss of function/KO/Germline	Homozygotes die within 24 h after birth. Heterozygotes display increased bone formation. High bone mass.	^476–478^
*GSK3β*	Loss of function/cKO/Sox2-Cre	Homozygotes died 24 h after birth. Complete cleft palate defect.	^479^
*GSK3β*	Loss of function/cKO/Col2α1-Cre	Normal skeletal growth or development.	^480^
*GSK3α* and *GSK3β*	Loss of function/KO/Germline	Dwarfism with shortened long bone and vertebra, and impairment of chondrocyte differentiation.	^481^
*Axin1*	Loss of function/KO/Germline	Homozygotes die at E9.5. Heterozygotes display rib fusion.	^482^
*Axin2*	Loss of function/KO/Germline	Malformations of skull structures (craniosynostosis). Accelerated ossification and increases in mineralization. Increased trabecular bone mass and bone formation rates. Increased osteoblast proliferation and differentiation. Decreased osteoclast formation. Shorter hypertrophic zones in the growth plate. Accelerated chondrocyte maturation.	^483–485^
*Apc*	Loss of function/cKO/Osteocalcin-Cre	APC cKO mice die within 2 weeks. Early onset, severe osteopetrosis. Significant accumulation of bone matrix in the femur. Significantly increased bone deposition associated with disturbances in bone architecture and composition. Rapid bone formation rate. Lack of osteoclasts. Marked abnormalities in vertebrae, long bones, and calvaria.	^486^
*Apc*	Loss of function/cKO/Col2α1-Cre	Homozygous APC cKO mice die perinatally due to severe defects in skeletogenesis. Craniofacial abnormalities, short trunk, an incomplete closure of both thoracic and abdominal cavities. Severe truncation of both upper and lower limbs. No cartilaginous primordia of pelvic bones. Heterozygotes do not show skeletal defect.	^487^
Functional Group: Transcription regulation			
*β-catenin*	Loss of function/cKO/Brn4-Cre (β-catenin exons 3–6)	Severe malformations of the hindlimbs. Truncation of tibia and fibula, and an absence of digits I–IV.	^488^
*β-catenin*	Loss of function/cKO/Prx1-Cre	Mice die at birth. Bone development defect. Shortened. Appendicular bones are shortened, partially fused, and lacked some distal structures. lack of mineralization in distal skeletal elements in the hindlimb. Delayed chondrocyte maturation.	^228^
*β-catenin*	Loss of function/cKO/Dermo1-Cre	Severe defects in skeletogenesis. Shortened limbs and a twisted body axis. Lack of bone but cartilage is present. No ossification. Disrupted osteoblast differentiation. Long bones are shortened, thickened, and bowed. Ectopic cartilage formation.	^227^
*β-catenin*	Loss of function/cKO/Col2α1-Cre	Mice die shortly after birth. Shortened limbs. Joint fusion. Some joints between the future tarsal bones in the ankle region were either missing or incompletely formed. Increased cartilage nodule formation. Craniofacial deformities characterized by a domed skull and a short snout, as well as short limbs.	^460,489^
*β-catenin*	Gain of function/Transgene/ Col2α1 promoter/enhancer (N-terminally truncated form of β-catenin)	Perinatal lethal. Dome-shaped heads and shorter limbs. Reduced cartilage formation and endochondral ossification. Joint fusion. Loss of cartilage tissue.	^460^
*β-catenin*	Gain of function/cKO/Prx1-Cre (β-catenin exon 3)	Mice die at birth. Limbs contain only tiny remnants of skeletal elements. Loss of skull bones.	^228^
*β-catenin*	Gain of function/cKO/Col2α1-Cre (β-catenin exon3)	Heterozygotes die around E18-E18.5 characterized by a very severe and generalized chondrodysplasia. Extremely small ribs, limbs, and vertebrae. Defective cartilage formation.	^489^
*β-catenin*	Gain of function/cKO/Brn4-Cre (Exon 3)	Enlarged limb size.	^488^
*β-catenin*	Loss of function/cKO/Col1α1-Cre	Low bone mass. Increased osteoclast activity. No change in osteoblasts.	^272^
*β-catenin*	Loss of function/cKO/Osteocalcin-Cre	β-catenin cKO mice die within 5 weeks. early onset, severe osteoporosis and is associated with defective osteoblast differentiation in vitro. Reductions in both the trabecular and cortical bone compartments. Dramatic reduction in mineralized cortical and trabecular bone. Marked abnormalities in vertebrae, long bones, and calvaria. Increased osteoclast number.	^486^
*β-catenin*	Loss of function/cKO/Osterix1-GFP::Cre (Tet-off)	Lack the membranous bone of cranial ossification center and complete loss of bone deposition. Failure of osteoblast progression to terminal Osteocalcin^+^ osteoblasts instead convert to a chondrocyte fate.	^269^
*β-catenin*	Loss of function/cKO/Osterix -Cre	Increased bone marrow adiposity and decrease in trabecular bone. Increased osteoclast-mediated bone resorption and decreased osteoblast-mediated bone formation. Cell fate shift of preosteoblasts to adipocytes.	^270^
*β-catenin*	Loss of function/cKO/LysM -Cre	Osteopenia. Reduction of the trabecular bone volume. Normal bone formation rate, osteoblast number and surface. Increased osteoclastogenesis.	^321^
*β-catenin*	Gain of function/cKO/Osterix1-GFP::Cre (Tet-off) (β-catenin exon3)	Heterozygotes die at birth. Shortened limbs. Intense and broader ossification center in the long bones. Delayed ossification in the skull bones. Abnormal wedge-shaped growth plate with very few identifiable hypertrophic chondrocytes. Lack of osteoclast.	^269^
*β-catenin*	Gain of function/cKO/Col2α1-Cre-ER^T2^ (β-catenin exon3)	Tamoxifen is administered to induce overexpression of β-catenin. Reduced articular cartilage. Complete loss of articular cartilage layers and formation of new woven bone in the subchondral bone area. Premature chondrocyte differentiation and OA-like phenotype. Lengthening of the hypertrophic region of cartilage. Advanced chondrocyte maturation and primary ossification center development.	^361,490^
*β-catenin*	Loss of function/cKO/Col2α1-Cre-ER^T2^	Delayed onset of chondrocyte hypertrophy and stunted progression to mature chondrocyte. Small hypertrophic zone, disorganized pre-hypertrophic cells, and no primary ossification center.	^490^
*β-catenin*	Loss of function/cKO/Osterix-Cre-ER^T2^	Tamoxifen is administered to induce conditional knockout of β-catenin. Severe osteopenia. Impaired osteoblast activity and increased osteoblast turnover. Increase in osteoclast number and activity. Marked increase in bone marrow adiposity.	^491^
*β-catenin*	Gain of function/cKO/Axin2-rtTA (Wnt responsive cells) + TRE-Cre (functions as a Doxycycline inducible Axin2-Cre) (β-catenin exon3)	Increases in expansion of skeletogenic precursors and the enhancement of bone ossification. Inhibition of osteoblast maturation into terminally differentiated osteoblasts.	^492^
*β-catenin*	Gain of function/cKO/PPARγ-tTA (Osteoclast progenitors) + TRE-Cre (functions as a Doxicycline inhibitable PPARγ-Cre) (β-catenin exon3)	Severe osteopetrosis. Increased trabecular BV/TV ratio, greater bone surface, Tb.N., and Tb.Th., accompanied by a smaller BS/BV ratio and Tb.Sp. Normal osteoclast proliferation but decreased osteoclast differentiation. Decreased osteoclast surface and numbers. Normal bone formation rate and mineral apposition rate.	^493^
*β-catenin*	Loss of function/cKO/PPARγ-tTA (Osteoclast progenitors) + TRE-Cre (functions as a Doxicycline inhibitable PPARγ-Cre)	Heterozygotes show osteoporosis. Reduced trabecular bone with a smaller BV/TV ratio, less bone surface, Tb.N, and Tb.Th, and a greater BS/BV ratio and Tb.Sp. Increased bone resorption and osteoclast surface/numbers. No change in bone formation, osteoblast surface/numbers, and bone formation/mineral apposition rates. Homozygotes display osteopetrosis, similar to the β-catenin gain-of-function mice. Decreased osteoclast precursor proliferation.	^493^
*β-catenin*	Loss of function/cKO/Dmp1-Cre	Homozygotes display low bone mass. Impaired bone mass accrual due to early-onset, progressive bone loss in the appendicular and axial skeleton with mild growth retardation and premature lethality. Growth retardation. Absence of Cancellous bone mass. Reduced cortical bone thickness. Increased osteoclast number and activity. Normal osteoblast function and osteocyte density.	^494^
*β-catenin*	Gain of function/cKO/Col1α1-Cre (β-catenin exon3)	Mice die a few days after weaning. Osteopetrosis. High bone mass. Defect in osteoclast differentiation. Normal osteoblast number.	^272^
*Tcf1*	Loss of function/KO/Germline	No overt phenotype. Low bone mass. Increased bone resorption. No change in bone formation parameters.	^272^
*Tcf1* Dominant negative *(Col2*α*1)*	Gain of function/Transgene (dominant-negative)/Col2α1 promoter	Dwarfism. Retarded mineralization in limbs, ribs, and vertebrae. Retarded endochondral ossification due to decelerated chondrocyte maturation. Reduced chondrocyte proliferation.	^495^
*Tcf4/Tcf7l2*	Loss of function/KO/Germline	Homozygotes *Tcf4*^*−/−*^ die shortly after birth. Mice carrying compound null mutations in Tcf4 and Lef1 show disrupted midfacial development and malformed teeth. Severe disruption of the morphology of facial skeletal elements but unimpeded chondrogenesis and osteogenesis.	^496,497^
*Lef1*	Loss of function/KO/Germline	Homozygotes show postnatal lethality. Lack of teeth. *Lef1*^*+/-*^ female mice show reduced trabecular bone mass, decreased osteoblast activity and bone formation. There is an age- and gender-dependent role for Lef1 in regulating bone formation and bone mass. Mice carrying compound null mutations in Lef1 and Tcf1 display defects in the development of limb buds.	^498–500^
*Lef1ΔN (a short isoform of Lef1*	Gain of function/Transgene/2.3 kb Col1α1 promoter	High bone mass. Increased trabecular bone volume and trabecular thickness. Increased bone formation and mineral apposition rates. Normal osteoblast surface area, osteoid surface area, and osteoid thickness. Normal osteoclast surface and activity.	^501^

*ALP* alkaline phosphatase, *APC* adenomatous polyposis coli, *BMC* bone mineral content, *BMD* bone mineral density, *BS/BV* bone surface/bone volume, *BV/TV* bone volume per total volume, *cKO* conditional knockout, *Col1A1/ColIα1* collagen type I alpha 1, *Col2α1* collagen type II alpha 1, *Col10α1* collagen type X alpha 1, *Dkk* Dickkopf, *DMP1* dentin matrix protein 1, *E17.5* embryonic day 17.5, *Fzd* Frizzled, *GSK3α* glycogen synthase kinase 3α, *GSK3β* glycogen synthase kinase 3β, *KO* knockout, *LEF* lymphoid enhancer-binding factor, *Lrp* low-density lipoprotein receptor-related protein, *OA* osteoarthritis, *SAP* serum amyloid P, *Sfrp* secreted Frizzled related protein, *Tb.N.* trabecular number, *Tb.Th* trabecular thickness, *Tb.Sp.* trabecular separation, *TCF* T-cell factor

### Wnt signaling in bone marrow mesenchymal stem cells (BM-MSCs)

BM-MSCs are MSCs residing in the bone marrow. As the common origins of osteoblasts, adipocytes, and chondrocytes, the tightly controlled lineage commitment of BM-MSCs is crucial in the maintenance of bone formation and homeostasis. The alteration of the commitment of BM-MSCs to osteoblasts and adipocytes occurs in bone pathological conditions, such as osteoporosis.^225,226^

Among the numerous signaling pathways involved in regulating the lineage commitment of BM-MSCs (BMP, Hedgehog, Notch, Wnt), Wnt signaling inhibits BM-MSC’s commitment to the adipogenic and chondrogenic lineages while promoting their differentiation into the osteoblasts.^227–229^ Wnt3a-induced canonical Wnt signaling stimulates osteogenic differentiation of MSCs by activating transcriptional co-activator with PDZ-binding motif (TAZ), which is a key transcriptional modulator of MSC differentiation.^230,231^ Furthermore, Wnt3a also enhances osteoblast differentiation and suppresses adipocyte differentiation of human BM-MSCs via non-canonical JNK signaling.^232^ Wnt3a and Wnt7b also promotes osteogenic differentiation and bone formation via PKCδ-mediated non-canonical Wnt signaling.^233^ The non-canonical Wnt5a suppresses PPARγ activation to suppress adipogenic differentiation and promote the osteogenic differentiation of MSCs^234^ and also induces osteoblast differentiation of BMSCs under mechanical stimulation.^235^ Wnt5a also shows promotion effect on chondrogenesis of MSCs by activating non-canonical Wnt signaling, such as Wnt/Ca^2+^ signaling pathway.^236,237^ Recently, Wnt7a shows the role to promote osteogenic differentiation of human MSCs by increasing Runx2 expression, which is mechanistically conducted by Wnt7a to promote the binding of TCF1 to the Runx2 promoter.^238^ Wnt10b enhances osteoblastogenesis and suppresses adipogenesis of mesenchymal progenitors, thus increasing bone formation and bone mass.^239^ The further study demonstrates that Wnt10b shifts mesenchymal cells toward osteoblasts, rather than adipocytes, by increasing the expression of osteogenic transcription factors (runt-related transcription factor 2 (Runx2), Dlx5, and Osterix) and suppressing the expression of adipogenic transcription factors (C/EBPα and PPARγ).^239,240^ Conversely, Wnt10b deficiency decreases mesenchymal progenitor activity and number, resulting in bone loss.^241^ In addition, Wnt6 and Wnt10a also facilitate osteogenic differentiation and suppress adipogenic differentiation of MSC via β-catenin.^242^ Besides, Dvl shows a role in regulating osteogenic differentiation of BM-MSCs.^243^ Following the osteogenic differentiation of BM-MSCs, the methylation level of Dvl decreases, which results in the elevated expression of Dvl,^243^ demonstrating Dvl as a promoter for osteogenic differentiation of BM-MSCs. Moreover, β-catenin is required for promoting osteoblast differentiation and inhibiting chondrocyte differentiation of mesenchymal progenitor cells, and the inactivation of β-catenin results in defective skeletal development.^227,228^ More recently, Matsushita et al. demonstrated that Wnt-mediated transformation of the bone marrow stromal cell (BMSC) identity orchestrates skeletal regeneration.^244^ They found that quiescent Cxcl12-creER^+^ perisinusoidal BMSCs differentiate into cortical bone osteoblasts solely during regeneration and quiescent Cxcl12-creER^+^ BMSCs transform into osteoblast precursor cells in a manner mediated by canonical Wnt signaling.^244^

Wnt signaling also shows key roles in mediating the function of numerous molecules in BM-MSCs to regulate cell differentiation capacity. Core-binding factor subunit β (Cbfβ), a non-DNA-binding partner of Runt-related transcription factors (Runx1, Runx2, and Runx3), plays a key role in governing osteoblast−adipocyte lineage commitment by enhancing β-catenin signaling.^229^ Z-DNA binding protein 1 (ZBP1), a member of the Zα family, increases osteogenic differentiation while suppressing adipogenic differentiation of mouse BM-MSCs.^245^ It was demonstrated that ZBP1 is required for β-catenin translocation into nuclei and is a novel regulator of bone and fat trans-differentiation via Wnt/β-catenin signaling.^245^ Serpin Family B Member 2 (SerpinB2) is a member of the clade B subgroup of serine protease inhibitors (serpins). Exogenous SerpinB2 protein inhibits osteoblast differentiation while the silencing of SerpinB2 promotes osteoblast differentiation of human BM-MSCs via the Wnt/β-catenin signaling pathway.^246^ Our previous work demonstrated that conditional knockout (cKO) of MACF1 in mesenchymal stem cells inhibited osteogenic differentiation of BM-MSCs and elevated bone surface adipocyte number, which results in decreased bone formation.^247^ Wu et al. identified a novel nuclear factor I/X (NFIX) - high-mobility group AT-Hook 1 (HMGA1) - Wnt/β-catenin regulatory axis that governs the cell fate of mouse BM-MSCs, favoring osteoblast differentiation and blocking adipocyte formation.^248^ HMGA1, as a downstream target of NFIX, functions by transcriptionally regulating LRP5 expression and thereafter activating canonical Wnt signaling.^248^ Moreover, Circ-FBLN1 (a circular RNA acting as a sponge for let-7i-5p) promotes cell proliferation and osteogenic differentiation of human BM-MSCs by regulating the let-7i-5p/FZD4 axis and repressing Wnt/β-catenin pathway.^249^ More recently, non-canonical Wnt signaling is shown to mediate the regulation of phosphate on human MSCs’ osteogenic differentiation.^250^ Phosphate treatment upregulated the expression of Wnt5b, Wnt11, and phosphorylated-c-Jun to promote osteogenic differentiation of human MSCs.^250^ Overall, by interacting with and regulating the activity of the aforementioned proteins and biomolecules, Wnt signaling plays a critical role in regulating BM-MSCs differentiation capacity.

### Wnt signaling in osteoblasts

Osteoblasts originate from BM-MSCs and are responsible for bone formation. Wnt signaling is critical for osteoblast function and most of its signaling components play important roles in regulating bone development and maintenance.^224,251^

#### The components of Wnt signaling in osteoblasts

Multiple Wnts including Wnt1, Wnt3a, Wnt5b, Wnt7b, Wnt10b, Wnt11, and Wnt16 regulate osteoblast differentiation and bone formation.^220,252–256^ Canonical Wnt signaling promotes osteoblast differentiation by directly activating the expression of the key bone-related transcription factor Runx2 via β-catenin/TCF1 signaling.^252^ Wnt16 promotes osteoblast differentiation and bone formation through canonical Wnt signaling.^256^ Besides, Tu et al. reported that non-canonical Wnt signaling promotes osteoblast differentiation and bone formation via activating G-protein-linked PKCδ.^253^ Recently, Lawson et al. demonstrated that inhibition of osteoblast-specific Wnt secretion alters skeletal homeostasis by suppressing bone formation and increasing bone resorption, reducing the anabolic response to mechanical loading, and demonstrating that Wnt ligand secretion is required for adult bone formation and homeostasis. Additionally, this indicates that osteoblast-derived Wnts are important in mediating the bone anabolic response to mechanical loading.^257^ Besides, Wnt primary receptors Fzds play an important role in osteoblast and bone formation. Albers et al. demonstrated that Fzd9 is required for osteoblast mineralization and bone formation.^258^ They developed an *Fzd9*^*−/−*^ mouse line and found that *Fzd9*^*−/−*^ mice displayed lower bone mass caused by decreased bone formation, with *Fzd9*^*−/−*^ primary osteoblasts showing defective matrix mineralization.^258^ Two co-receptors of Wnt signaling, LRP5/6, are required for optimal Wnt signaling in osteoblasts, and each plays a key distinct role in bone formation. Lrp6, rather than Lrp5, is crucial for mediating Wnt3a signaling in osteoblasts and shows different effects on osteoblastic gene expression.^259^ Lrp5 is required for the late stages of differentiation while Lrp6 is required for the early stages of osteoblast differentiation.^260^ Mice with a targeted disruption of *Lrp5* develop a low bone mass phenotype, which becomes evident postnatally and is secondary to decreased osteoblast proliferation.^261^ A LRP5 gain of function mutation in osteoblasts causes increased bone mass/bone mineral density (BMD) in human and transgenic mice and an increase in the number of active osteoblasts, further confirming the important role of LRP5 in bone formation and homeostasis.^262,263^ LRP6 knockout mice were perinatal fatal, with truncations of the axial skeleton and limb defects.^45,47^ Moreover, Lrp4 and Lrp8 were demonstrated to play important roles in bone remodeling by modulating Wnt signaling.^264,265^ Lrp4 binds to sclerostin to facilitate the inhibitory effect of sclerostin on Wnt/β-catenin signaling, thus inhibiting bone formation.^166^ The osteoblast/osteocyte-specific *Lrp4* knockout induces elevated serum sclerostin, promotes osteoblast function, and results in an increase in bone mass.^266^ Lrp8 was demonstrated to play a role in Wnt3a-induced osteoblast differentiation.^267^ Furthermore, Dvl is involved in osteoblast differentiation. Zhou et al. found that ubiquitin-specific peptidase 4 (USP4) inhibited Wnt/β-catenin signaling by removing Lysine-63 linked poly-ubiquitin chain from Dvl and promoting β-catenin polyubiquitination, which leads to decreased cytosolic β-catenin and downstream signaling.^268^ USP4 inhibits osteoblast differentiation while USP4 depletion promotes osteoblast differentiation.^268^ These findings demonstrate Dvl as a target of USP4 in regulating osteoblast differentiation. In addition, several studies reveal the necessity of β-catenin in the osteoblast lineage.^227,269,270^ Deletion of β-catenin in a different stage of osteoblastic differentiation in mice causes low bone mass phenotype due to both defective osteoblast differentiation along with increased osteoclastic bone resorption that is caused by decreased OPG (osteoprotegerin)/RANKL (receptor activator of NF-κB ligand) ratio.^271,272^ All these findings together reveal the importance of Wnt signaling components in controlling osteoblast function.

#### The modulators of Wnt signaling in osteoblasts

The modulators of Wnt signaling also play important roles in osteoblast. R-spondins, activators of Wnt signaling, are highly expressed in skeletal tissues and promote osteoblast differentiation.^273,274^ MACF1, an activator of Wnt/β-catenin, shows promotion effects on osteoblast proliferation, differentiation, and bone formation.^125,126,275–277^ sFRPs, the largest family of Wnt inhibitors, are demonstrated as important regulators of osteoblast function and bone formation. Deletion of *sFRP1* activates Wnt canonical signaling, which increases the expression of Runx2 and osteocalcin, thus enhancing osteoblast differentiation and bone formation.^252^ Besides, *sFRP1* deficiency inhibits osteoblast lineage apoptosis and enhances osteoblast proliferation.^278^ *sFRP1*^*−/−*^ mice exhibit increased trabecular bone mineral density while sFRP1 transgenic mice display decreased bone formation and trabecular bone mass.^278,279^ Moreover, sFRP2 and sFRP4 are critical for proper distal limb formation and bone formation.^280,281^ Dkk1, a member of the Dkk family (Wnt inhibitor), is a key negative regulator of osteoblasts. Endogenous Dkk1 is primarily expressed in osteoblasts and osteocytes.^282^ Dkk1 suppresses osteoblast differentiation and bone formation by binding to LRP5/6 to inhibit Wnt signaling.^282^ Osteoblast overexpression of Dkk1 in transgenic mice induces diminished osteoblastic bone formation and severe osteopenia,^282^ while heterozygous *Dkk1*-deficient (*Dkk1*^*+/-*^) mice display increased osteoblast number, mineral apposition, bone formation, and bone mass.^283^ Dkk1 overexpression in primary calvaria cells completely inhibits osteoblast differentiation and mineralized nodules in vitro.^283^ Dkk4 also functions as an inhibitor of osteoblast differentiation by suppressing Wnt/β-catenin signaling.^284^ Unlike Dkk1 and Dkk4, Dkk2 is required for terminal osteoblast differentiation and mineralized matrix formation, with *Dkk2*^*−/−*^ mice showing decreased bone formation.^285^ Krm1 and Krm2, co-receptors of Dkk1, interact with Dkk1 to attenuate Wnt/β-catenin signaling during limb development, as shown by *Krm1*^*−/−*^*Krm2*^*−/−*^ mice presenting with increased bone formation.^146^ Overexpression of Krm2 in osteoblasts in transgenic mice leads to severe osteoporosis.^286^ In addition, sclerostin, another inhibitor of Wnt signaling, is crucial for osteoblast function and bone formation.^287^ Sclerostin suppresses proliferation/differentiation and promotes apoptosis of osteoblasts and overexpression of sclerostin suppressing bone formation.^133,163,288,289^ *SOST*^−/−^ mice exhibit increased high bone mass due to increased bone formation, while *SOST* transgenic mice exhibit low bone mass due to decreased bone formation^290,291^ (for review, see Sebastian et al.^292^).

#### Other molecules modulate osteoblast function via Wnt signaling

Wnt signaling is also involved in mediating the function of numerous biomolecules in osteoblasts. Runx1, a highly expressed protein in osteoblast, maintains osteoblast differentiation by up-regulating the Wnt/β-catenin signaling pathway.^293^ Chemerin, a novel adipocyte-derived signaling molecule, shows an inhibitory effect on osteoblast differentiation and proliferation through the inhibition of Wnt/β-catenin signaling.^294^ Large conductance calcium-activated potassium (BK) channels encoded by the Kchma1 gene are among the K^+^ channels that have unusually large single-channel conductance.^295^ Jiang et al. uncovered that the BK channel is essential for osteoblast proliferation, differentiation, and bone formation via the Wnt/β-catenin pathway.^296^ Conditional knockout of Kcnma1, which encodes the pore-forming α-subunits of BK, results in a decrease in β-catenin in the Wnt/β-catenin signaling pathway, which inhibits Runx2 expression and leads to bone loss.^296^ More recently, miR-12200-5p was demonstrated to significantly inhibit osteoblast differentiation and bone formation by simultaneously targeting multiple members of the Wnt signaling, including APC, TCF4, TCF7, Wnt3a, Wnt5a, and LRP6.^297^

Recently, Wnt signaling was demonstrated to be crucial for modulating cellular metabolism in osteoblasts.^298^ Wnt signaling stimulates aerobic glycolysis, glutamine catabolism, and fatty acid oxidation in osteoblast-lineage cells.^298^

#### Wnt signaling in osteocytes

Osteocytes are the most abundant cells in bone. They are terminally differentiated osteoblasts embedded within the mineralized matrix.^299^ Osteocytes help orchestrate the signaling that regulates osteoblasts and osteoclasts during bone remodeling.^299^ Moreover, they are believed as mechanosensory cells.^299,300^ During the terminal mineralization process, the Wnt/β-catenin pathway is downregulated. The activation of Wnt/β-catenin signaling in osteocytes suppresses dendrite development, inhibits dentin matrix protein 1 (DMP1) expression, and alters normal mineral crystallinity.^301^ Moreover, Wnt signaling is involved in osteocytes sensing mechanical stimuli and regulating bone remodeling to coordinate normal bone homeostasis.^291,302,303^

#### The components of Wnt signaling in osteocytes

Joeng et al. reported that osteocyte-specific Wnt1 loss- or gain-of-function mice presents low bone mass or high bone mass, respectively.^304^ Besides, Wnt receptor LRP5 shows a key role in osteocytes. LRP5-mediated Wnt signaling in osteocytes contributes to the maintenance of mechanical properties and bone mass.^305^ Mice with an osteocyte-specific deletion of *Lrp5* exhibit reduced bone mass, lower Young’s modulus of bone, and significantly diminish load-driven bone formation.^305^ In addition, β-catenin, a key mediator of Wnt/β-catenin signaling, is necessary for maintaining osteocyte viability and for the ability of osteocytes to respond to mechanical stimuli. Osteocyte-specific β-catenin deficient mice exhibit low bone mass phenotype in association with increased osteoclast number and bone resorption^303^ and do not respond to mechanical loading.^306^ Tu et al. also found that activation of osteocytic β-catenin signaling increases both osteoclasts and osteoblasts, resulting in bone gain,^307^ identifying osteocytes as central target cells of the anabolic actions of Wnt/β-catenin signaling in bone. Therefore, the components of Wnt signaling are critical in osteocytes by mediating osteocyte mechanotransduction and the regulatory role of osteocytes in osteoblasts and osteoclasts.

#### The modulators of Wnt signaling in osteocytes

Osteocytes express several inhibitors of the Wnt/β-catenin pathway, including sclerostin, Dkk1, and sFRP1, all of which regulate bone mass. Sclerostin is well known for its specific expression in osteocytes,^163,308^ with osteocytes secreting sclerostin via their dendritic attachments.^309^ The secreted sclerostin functions on osteoblasts to suppress the Wnt/β-catenin pathway, thus inhibiting osteoblast differentiation and bone formation^133^ (see Wnt signaling in osteoblasts for detail). Moreover, in line with the mechanical response of osteocytes, sclerostin expressed by osteocytes is demonstrated as a mechanosensitive protein, and its expression is regulated by mechanical stimuli. Mechanical loading reduces the sclerostin expression and promotes bone formation, while mechanical unloading increases sclerostin expression and inhibits bone formation, both processes involving Wnt signaling.^291,310^ Interestingly, both *SOST*^−/−^ mice and DMP1-*SOST* transgenic mice exhibit reduced sensitivity to mechanical stimulation.^291,311^ *SOST*^−/−^ mice are resistant to mechanical unloading-induced bone formation reduction in association with unaltered Wnt/β-catenin signaling.^291^ While DMP1-*SOST* transgenic mice exhibit reduced load-induced bone formation and unaltered Wnt signaling.^311^ Therefore, sclerostin is critical for mechano-transduction and mechanical stimuli regulating bone formation via Wnt signaling. Besides sclerostin, osteocytes express sFRP1 and Dkk1 to inhibit osteoblast differentiation and bone formation (see Wnt signaling in osteoblasts for detail). The expression of Dkk1 is also regulated by mechanical stimuli.^310^

### Wnt signaling in osteoclasts

Osteoclasts are the bone-resorbing cells that originated from hematopoietic monocyte/macrophage lineage cells and are involved in the bone remodeling process.^312^ Recent studies demonstrate that Wnt signaling plays a direct role in regulating osteoclast function. Targeted deletion of β-catenin in osteoclast precursors inhibits the precursor proliferation and accelerates osteoclast differentiation, while deletion of β-catenin in more committed stages of osteoclast differentiation enhances the rate of cell specialization.^313–315^ Additionally, Ruiz et al. reported that conditional deletion of β-catenin in Cathepsin K-expressing cells increases osteoclast activity.^315^ These findings demonstrate that Wnt signaling promotes osteoclast progenitor proliferation and suppresses osteoclast commitment and differentiation. In addition, Dvl, a key component of Wnt/β-catenin signaling, plays a role in regulating osteoclastogenesis by interacting with PTH1R (type 1 parathyroid hormone receptor).^316^ Mutation of Dvl results in inhibition of β-catenin activation and blocks osteoclastogenesis under PTH induction.^316^ More recently, Weivoda et al. found that Wnt signaling suppresses osteoclast differentiation by activating canonical and non-canonical cyclic adenosine monophosphate (cAMP)/protein kinase A (PKA) pathways.^317^ Besides, sFRP4, a Wnt inhibitor, dramatically suppresses the osteoclast differentiation by suppressing non-canonical Wnt/ROR2/JNK signaling.^318^

Wnt signaling in osteoblasts and osteocytes also indirectly regulates osteoclast differentiation. Wnt signaling in osteoblasts inhibits osteoclast differentiation by suppressing the expression of RANKL and increasing the expression and secretion of the RANKL decoy receptor, OPG.^271,272,319,320^ In addition, osteocyte Wnt signaling also represses osteoclast differentiation by increasing the expression of OPG, thus decreasing the RANKL/OPG ratio.^303^ However, Albers et al. reported that there is increased osteoclastogenesis in *Fzd8*-deficient mice, which was independent of OPG, suggesting a direct negative influence of canonical Wnt signaling on osteoclastogenesis.^321^ Moreover, non-canonical Wnt5a secreted from osteoblast lineage cells promotes osteoclastogenesis and bone resorbing ability by increasing RANK (receptor activation of nuclear factor-κB) in bone marrow macrophages via the ROR2/JNK non-canonical signaling.^322,323^ Wnt5a-ROR2 noncanonical signaling is also required for the formation of actin ring and the bone-resorbing activity of osteoclasts.^324,325^ In contrast, Wnt16 suppresses osteoclast differentiation by activating non-canonical Wnt signaling and suppressing RANKL-induced activation of NF-κB and expression of NFATC1.^326^ Although most components of the Wnt signaling pathway (e.g., Wnts, Fzds, and LRPs) are expressed by osteoclasts,^327^ the role of Wnt signaling in osteoclasts still needs further investigation.

## Wnt signaling in bone disease

**Fig. 5:**
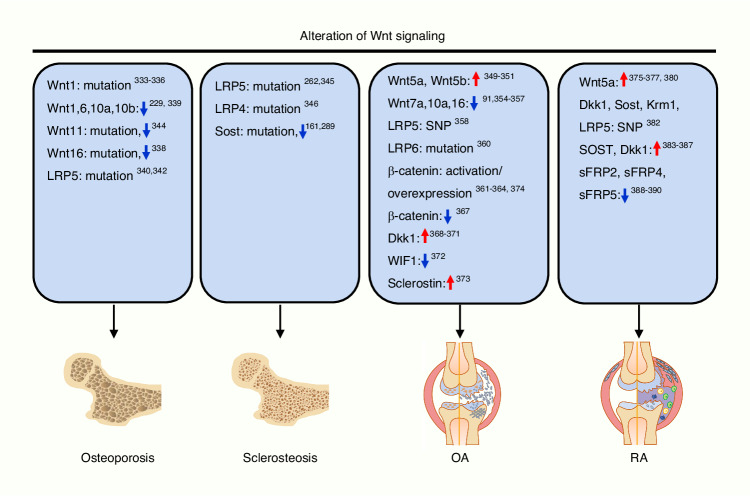
Wnt signaling involved in bone disease. The Wnt signaling is involved in bone disease. including osteoporosis, sclerosteosis, osteoarthritis (OA), and rheumatoid arthritis (RA), as shown in Table 3 for detail

The necessity of Wnt signaling (including Wnt ligands, receptors, intracellular components, transcription factors, and antagonists) for bone development, formation, and homeostasis has been broadly studied in mouse models (Table 2). Given the necessity of Wnt signaling for bone, it is not surprising that aberrant Wnt signaling results in various bone diseases, such as osteoporosis, sclerosteosis, osteoarthritis (OA), and rheumatoid arthritis (RA) (Table 3, Fig. 5).^328–332^

**Table 3:** Wnt signaling in bone diseases

Molecule	Nature of miscues	Diseases	Symptoms	Key reference
Wnt1	Mutation	Osteoporosis, osteogenesis imperfecta	Low BMD and bone strength, low-impact vertebral and peripheral fractures	^333–336^
Wnt3	Homozygous nonsense mutation	Tetra-amelia	Complete absence of all four limbs and other anomalies	^337^
Wnt10b	Decreased expression	Osteoporosis	Severe osteoporosis with substantial accumulation of marrow adipocytes	^229^
Wnt11	Loss-of-function mutation	Early onset osteoporosis	Low bone mineral density that results in increased risk of fracture in children and young adults	^344^
Wnt16	Missense mutation	Osteoporotic fractures	Low cortical bone thickness, BMD, and bone strength, and increase of risk of fracture	^338^
LRP5	Homozygous mutation	Autosomal recessive disorder OPPG	Severe, early-onset osteoporosis and abnormal eye vasculature	^340,342^
SOST	Loss-of-function mutation	Sclerosteosis, van Buchem disease	High bone mass, progressive bone overgrowth due to increased bone formation	^161,289^
LRP5	Point mutation	HBM trait	Dense bones	^262,345^
LRP4	Mutation	Sclerosteosis	Bilateral syndactyly of the third and fourth finger, severe sclerosis of the calvarium, femur, radius, and ulna.	^346^
Wnt5a	Increased expression	OA	Inflammation, ECM destruction, cartilage damage	^349,350^
Wnt5b	Increased expression	OA	Inflammation, cartilage damage	^349,351^
Wnt7a	Decreased expression	OA	Inflammation, cartilage damage	^354^
Wnt10a	Decreased expression	OA	Accumulation of senescent cells, inflammation, cartilage damage	^91^
Wnt16	Decreased expression	OA	Inflammation, deteriorated articular cartilage integrity, chondrocyte apoptosis	^355–357^
LRP5	Haplotype (C-G-C-C-A)	OA	Bone spur (osteophyte), joint space narrowing and pathological hardening of subchondral bone (sclerosis)	^358^
LRP6	Heterozygous loss-of-function mutation	OA	Cartilage degradation, bone spur (osteophyte) formation, joint space narrowing and pathological hardening of subchondral bone (sclerosis)	^360^
β-catenin	Activation or Overexpression	OA	Cartilage degradation, inflammation (in knee joint, hip joint, temporomandibular joint, and facet joint)	^361–364,374^
β-catenin	β-catenin-knockout specific in SFZ	OA	Cartilage degradation, inflammation	^367^
Dkk1	Increased expression	OA	Cartilage deterioration, inflammation, chondrocyte apoptosis	^368–371^
WIF-1	Low expression	OA	Cartilage degradation, bone spur (osteophyte) formation, joint space narrowing and pathological hardening of subchondral bone (sclerosis)	^372^
Sclerostin	Increased expression	OA	Joint degeneration, inflammation	^373^
Wnt5a	Increased expression	RA	Inflammation, joint destruction, FLS migration and invasion	^375–377,380^
Dkk1, Sost, Krm1, LRP5	SNP	RA	Inflammation, joint destruction	^382^
Dkk1 and SOST	Increased expression	RA	Inflammation, joint destruction	^383–387^
sFRP2	Decreased expression	RA	Inflammation, joint destruction, FLS activation	^388^
sFRP4	Decreased expression	RA	Inflammation, joint destruction, FLS activation	^389^
sFRP5	Decreased expression	RA	Inflammation, joint destruction, FLS activation	^390^

*BMD* bone mineral density, *Dkk* Dickkopf, *FLS* fibroblast-like synoviocytes, *GSK3β* glycogen synthase kinase 3β, *HBM* high bone mass, *LRP* low-density lipoprotein receptor-related protein, *OA* osteoarthritis, *OPPG* osteoporosis-pseudoglioma syndrome, *RA* rheumatoid arthritis, *sFRP* secreted Frizzled related protein, *SFZ* superficial zone, *SNPs* single nucleotide polymorphisms, *WIF* Wnt inhibitory factor

### Wnt signaling and osteoporosis

Osteoporosis is a degenerative disease characterized by low bone mass and deteriorative microarchitecture of bone. The alteration of Wnts causes human skeletal diseases, with mutations in Wnts such as Wnt1 causing osteoporosis and osteogenesis imperfecta.^333–336^ Studies demonstrate that Wnt1 mutation results in decreased β-catenin and thus the decreased Wnt/β-catenin signaling, which causes osteoporosis.^333,335^ Wnt3 expression is essential at the early stages of human limb formation. The homozygous nonsense mutation in the *Wnt3* gene, which truncates Wnt3 at its amino terminus, results in tetra-amelia, a rare human genetic disorder characterized by the complete absence of all four limbs and other anomalies.^337^ Moreover, missense mutations of Wnt16 are associated with osteoporotic fractures.^338^ Jing et al. found that Wnt signaling is inhibited persistently in BM-MSCs during osteoporosis and histone acetylation levels on Wnt genes (Wnt1, Wnt6, Wnt10a, and Wnt10b) are decreased in BM-MSCs from ovariectomized (OVX) mice.^339^ Besides, the homozygous mutation in *LRP5*, a co-receptor for Wnts, results in the autosomal recessive disorder osteoporosis-pseudoglioma syndrome (OPPG),^340^ a syndrome exhibiting severe, early-onset osteoporosis and abnormal eye vasculature.^341^ More recently, Astiazaran et al. identified a novel homozygous LRP5 mutation in Mexican patients with OPPG.^342^ In addition, Wnt10b expression is regulated by Cbfβ/Runx2 and the Cbfβ deficient mice in osteoblast lineage exhibit severe osteoporosis.^229^ Besides, ROR1/2, the coreceptor for activating non-canonical Wnt signaling, plays important roles in development, regeneration, and diseases of the bone.^343^ More recently, Wnt11 is identified as a new gene associated with early onset osteoporosis with loss-of-function inhibiting bone formation through both canonical and non-canonical pathways.^344^

### Wnt signaling and sclerosteosis

Sclerosteosis is a rare bone disease characterized by increased bone density in association with bone overgrowth. The discovery of sclerosteosis and van Buchem disease, which are rare high bone mass genetic disorders caused by *SOST* loss-of-function mutations, exemplifies the critical role of sclerostin in bone health.^161,289^ Humans lacking sclerostin display progressive bone overgrowth due to increased bone formation. Moreover, point mutation of *LRP5* (G171V) causes high bone mass trait.^262,345^ Mechanistically, point mutation of *LRP5* (G171V) impairs the inhibition effect of Dkk1 on Wnt signaling and thus results in increased Wnt signaling activity, which leads to high bone density.^345^ Fijalkowski et al. detected a novel LRP4 mutation in a patient with sclerosteosis.^346^ They found the replacement of the arginine residue on position 1 170 of LRP4 by glutamine, which impaired the binding between LRP4 and sclerostin, resulting in decreased inhibition of sclerostin on Wnt/β-catenin signaling.^346^ This study indicates that LRP4 is an anchor for sclerostin and responsible for sequestering the sclerostin.

### Wnt signaling and osteoarthritis (OA)

OA is the most common age-related degenerative joint disease, which is characterized by cartilage damage, synovial inflammation, osteophyte formation, and subchondral bone sclerosis.^347^ Wnts also play a key role in OA. Wnt5a is upregulated in OA-like chondrocytes and is involved in Col2A1 degradation.^348^ Furthermore, the elevated Wnt5a is detected in OA patients.^349,350^ Wnt5a promotes chondrocyte catabolic activity presented as reducing the expression of ACAN and Col2A1 but increasing the expression and secretion of matrix metalloproteinases (MMP1, 3, and 13) via non-canonical Wnt signaling including CaMKII and JNK,^349^ while Wnt5a induces the abnormal differentiation in osteoblast via both the non-canonical Wnt/PCP signaling and Wnt/Ca^2+^ signaling in OA osteoblasts.^350^ Similarly, upregulation of Wnt5b is observed in OA,^351,352^ suggesting the promotion effect of Wnt5b on OA. Wnt5b inhibits chondrogenic differentiation, promotes fibrosis by increasing collagen type I expression, and increases MMP13 expression in SMSCs (synovial resident mesenchymal stem cells), leading to joint degeneration.^353^ All these findings demonstrate the promotion effect of Wnt5a and Wnt5b on OA development. However, Wnt7a and Wnt10a show a protective effect on OA. There is a negative correlation between Wnt7a expression and the expression of matrix metalloproteinase (MMP) and IL-1β in human OA cartilage specimens.^354^ Wnt7a suppresses IL-1β-induced MMP and iNOS gene expression in primary human articular chondrocytes and attenuates articular cartilage damage in OA mice.^354^ Wnt10a specifically cleans up the senescent OA SMSCs (synovial resident mesenchymal stem cells) by inducing cell apoptosis.^91^ Mechanistically, Wnt10a activates non-canonical Wnt/Ca^2+^ signaling.^91^ Wnt16 also shows protective effect on OA.^355^ This is indicated by the findings that global knockout of Wnt16 (*Wnt16*^*−/−*^) or chondrocyte-specific knockout of Wnt16 in mice promote OA development with decreased expression of lubricin, increased chondrocyte apoptosis, upregulated MMP13 and Col10a1 (Collagen type X alpha 1) expression, and deteriorated articular cartilage integrity.^356,357^ Mechanistically, Wnt16 functions through both canonical and non-canonical Wnt signaling. Nalesso et al. demonstrated that Wnt16 antagonizes excessive Wnt/β-catenin activation by reducing the capacity of Wnt3a to activate the signaling, thus protecting cartilage in OA.^356^ Tong et al. found that Wnt16 activates PCP/JNK and crosstalks with the mTORC1-PTHrP pathway to inhibit chondrocyte hypertrophy during OA pathogenesis.^357^ Besides, LRP5 shows a key role in the pathogenesis of OA at a genetic level.^358,359^ Heterozygous loss-of-function mutation in LRP6 also leads to suppression of Wnt/β-catenin signaling and deterioration of degenerative OA after ligament and meniscus injury.^360^ Evidences demonstrate the important role of β-catenin in OA. Activation or overexpression of β-catenin leads to OA development in knee joint,^361^ hip joint,^362^ temporomandibular joint^363^ and facet joint.^364^ Therefore, inhibition of β-catenin signaling shows therapeutic effect on OA. Zhu et al. found that AMPK (adenosine 5’-monophosphate-activated protein kinase) activator metformin blocked β-catenin nucleus translocation by inhibiting β-catenin^S552^ phosphorylation and showed chondro-protective effect in OA progression,^365,366^ suggesting that AMPK activation may inhibit OA development partially through inhibition of β-catenin signaling. However, Xuan et al. found that superficial zone (SFZ)-specific β-catenin-knockout accelerates OA development while SFZ-specific β-catenin activation suppresses cartilage degeneration.^367^ They found that β-catenin deficiency decreases the expression of *Prg4*, the encoding gene for lubricin, while β-catenin activation increases Prg4 expression in SFZ cells.^367^ Moreover, Wnt inhibitors, such as Dkk1, WIF-1, and sclerostin, also play a role in OA. Dkk1 is indicated to be positively correlated with OA. Weng et al. found the increase of Dkk1 in the cartilages of OA patients in association with increased inflammatory cytokines.^368^ They demonstrated that Dkk1 mediates chondrocyte apoptosis by suppressing nuclear β-catenin accumulation and Akt activation and contributes to cartilage deterioration and OA.^368^ They further found that Dkk1 antisense oligonucleotide (Dkk1-AS) treatment decreased the OA-associated increase of Dkk1 and abrogated chondrocyte apoptosis in OA in rats.^369^ Dkk1 is also upregulated in OA cartilage^370^ and synovial fluid, but there is no significant difference in the serum Dkk1 concentration between the OA patients and healthy controls.^371^ However, a significant lower expression of WIF-1 is found in OA chondrocytes than in normal chondrocytes.^372^ Overexpression of WIF-1 increases cell proliferation and suppresses apoptosis of OA chondrocytes by eliminating high reactive oxygen species (ROS) and reducing the secretion of MMPs.^372^ Most studies show that sclerostin is increased in chondrocytes as a protective mechanism in OA to prevent further degeneration of joint.^373^ Besides the Wnt signaling-related components, Runx1, one key transcription regulator for cartilage formation, inhibits OA development by reducing the level of active β-catenin, thus inhibiting Wnt/β-catenin signaling.^374^

### Wnt signaling and rheumatoid arthritis (RA)

RA is an autoimmune disease characterized by damage of cartilage and bone due to inflammation. Wnt signaling shows critical roles in RA. Wnt5a is highly expressed in synovial fibroblasts in RA patients and promotes the expression of inflammatory cytokines in synovial fibroblasts.^375–377^ Transfection of normal fibroblasts with a Wnt5a expression vector induces the expression of inflammatory cytokines.^378^ Meanwhile, inflammatory cytokine enhances Wnt5a expression in RA synoviocytes.^379^ Recently, Rodriguez-Trillo et al. found that Wnt5a specifically promoted migration and invasion of RA FLS (fibroblast-like synoviocytes) and induced the expression of inflammatory cytokines through non-canonical Wnt/Ca^2+^ and ROCK pathways.^380^ All these findings demonstrate the promotion effect of Wnt5a on RA (for review, see Huang et al.^381^). LRP5 and Krm1 are associated with joint destruction in RA patients.^382^ By studying 1 418 patients with RA in four cohorts, de Rooy et al. found that in the Leiden early arthritis clinic cohort, six *Dkk1*, three *Sost*, one *Krm1*, and 10 *LRP5* SNPs are significantly associated with radiological progression of joint destruction.^382^ Studies further indicate the important involvement of sclerostin and Dkk1 in RA development.^383^ The serum sclerostin/SOST and Dkk1 are significantly higher in RA patients than in controls and correlate with bone erosion and inflammation.^383,384^ Sclerostin is upregulated in FLS of RA patients but inhibition of sclerostin accelerates TNFα-dependent inflammatory joint destruction in RA mice, demonstrating a protective role of sclerostin in TNF-mediated inflammation.^385^ Since Dkk1 is upregulated by TNFα, some studies indicate that treatment with TNFα inhibitors, such as Certolizumab pegol, decreases the serum concentration of Dkk1 in RA patients.^386,387^ In addition, RA-associated osteoporosis might be the result of both increased bone resorption and decreased bone formation, due to increased TNFα-driven osteoclast activity and overexpression of Dkk1.^387^ Besides, *Dkk1* and *Sost* SNPs and the interactions between SNPs on *Dkk1* and *Sost* are associated with RA.^382^ sFRP2, sFRP4, and sFRP5, antagonists of Wnt signaling, show a suppressive effect on RA.^388–390^ sFRP2 inhibits the proliferation of RA fibroblast-like synovial cells and the expression of IL-6 and IL-8 and inhibits RA pathogenesis through suppressing Wnt/β-catenin signaling.^388^ Mechanistically, DNA methylation plays a key role in regulating the expression of sFRP2 and sFRP4 and the activation of Wnt/β-catenin signaling in RA.^388,389^ sFRP5 shows an anti-inflammatory role in FLS in RA patients by downregulating c-Jun N-terminal kinase.^390^ Furthermore, other molecules are also involved in RA by regulating Wnt signaling. Acid-sensing ion channel 1a (ASIC1a), phospholipase D1 (PLD1), Aquaporin 1, and neuron navigator 2 (NAV2) all show a promotive effect on RA by activating Wnt/β-catenin signaling.^391–394^ ASIC1a, PLD1, Aquaporin 1, and NAV2 promote cell proliferation, migration, invasion, and inflammation of RA FLS through activating the Wnt/β-catenin pathway.^391–394^ Moreover, noncoding RNAs are involved in RA by regulating Wnt signaling. Sun et al. found that long noncoding RNA (lncRNA) OIP5-AS1 promotes the occurrence and development of RA by downregulating the expression of miR-410-3p, which increases Wnt7b expression and activates the Wnt/β-catenin pathway.^395^ Wang et al. reported that LINC00152 increased the proliferation of RA FLS by promoting the Wnt/β-catenin pathway.^396^ Mir-125a-3p suppresses cell proliferation and inflammation of RA fibroblast-like synovial cells by inactivating the Wnt/β-catenin pathway.^397^

## Targeting wnt signaling in bone disease treatment

The important involvement of Wnt signaling in bone formation, homeostasis and diseases drives extensive research efforts to target Wnt signaling for treating bone diseases. Studies show the therapeutic effects on bone diseases in both animal models and clinical trials by targeting either the extracellular molecules, cytosol components, or nuclear components of Wnt signaling (Table 4).

**Table 4:** Modifiers for Wnt signaling to treat bone diseases

Modifier	Molecular Target	Function	Effect on Wnt Signaling Pathway	Diseases/Therapeutic effect	Key Reference
Overexpression	Wnt4	Overexpresses Wnt4	Activates non-canonical Wnt signaling	Osteoporosis/Prevention	^398^
Genetic deletion	Wnt5a	Inhibits Wnt5a expression	Inhibition	RA/Alleviation	^406,407^
Ginkgolide B	Wnt5a	Inhibits Wnt5a expression	Inhibition	RA/Alleviation	^409^
Resveratrol	Wnt5a	Inhibits Wnt5a expression	Inhibition	RA/Alleviation	^410^
miR-21	Wnt	Inhibits Wnt expression	Inhibition	RA/Alleviation	^411^
Adenovirus-Wnt16	Wnt16	Increase Wnt16 expression	Activates PCP/JNK	OA/Alleviation	^357^
Wnt mimetics	Wnt	Binds to Fzd and LRPs	Activation	Osteoporosis, aging and long bone fracture/Induce rapid and robust bone building effects, correct bone mass deficiency and bone defects, improve the therapeutic effects of antiresorptive bisphosphonates and anti-sclerostin antibody	^412^
Wnt-induced osteogenic tissue model	Wnt	Maintain Wnt.	Activation	Bone defects/Maintain the osteogenesis of human skeletal stem cells and repair bone defects	^413^
SM04690 (Lorecivint, LOR)	Wnt pathway	Inhibits Wnt signaling pathway	Inhibition	OA/Improve the pain and cartilage degradation	^402–405^
Dkk1-AS	Dkk1	Inhibits Dkk1 expression	Activation	Estrogen deficiency induction of bone loss and glucocorticoid-induced bone loss/Alleviation	^414,415^
Exosomal miR-196a from BM-MSCs	Dkk1	Inhibits Dkk1 expression	Activation	Osteoporosis/In vitro study shows the promotion effect on osteogenic differentiation	^416^
MiR-483-3p	Dkk2	Inhibits Dkk2 expression	Activation	Osteoporosis/In vitro study shows the promotion effect on bone formation process by increasing osteoblast proliferation, preosteoblast differentiation into mature osteoblasts, and new bone matrix formation	^417^
AdDkk1	Dkk1	Upregulates Dkk1 expression	Inhibition	OA/Inhibits OA cartilage destruction	^370^
Dkk1-AS	Dkk1	Inhibits Dkk1 expression	Activation	OA/Reduce the OA-associated increase of Dkk1 and abrogate chondrocyte apoptosis	^369^
Romosozumab (a humanized monoclonal anti-sclerostin antibody)	Sclerostin	Inhibit the function of sclerostin by binding to sclerostin	Activation	Osteoporosis/Increase BMD and reduces fragility fractures in both male and female osteoporotic patients	^420–426^
Sclerositn small-molecule inhibitors	Sclerostin	Inhibit the function of sclerostin	Activation	Bone defects/Promote osteogenesis	^427^
Bispecific antibody	Sclerostin and Dkk1	Inhibit the function of sclerostin and Dkk1	Activation	Bone fracture/Superior bone repair activity	^428^
Sclerostin antibody/Dkk1 antibody combination	Sclerostin and Dkk1	Inhibits the function of sclerostin and Dkk1	Activation	Osteoporosis/Increase more cancellous bone mass	^429^
WIF-1 cDNA plasmid transfection	WIF-1	Overexpression of WIF-1	Inhibition	OA/Promote proliferation and suppress apoptosis of OA chondrocytes	^372^
Lithium/Lithium chloride	GSK3β	Inhibition of GSK3β from phosphorylating β-catenin	Activation	Osteoporosis/Promotes osteogenic differentiation and increases bone mass	^431,432^
Transgene method	MACF1	Overexpression of MACF1	Activation	Osteoporosis/Prevent aging induced osteoporosis	^433^
Daphnetin	Not clear	Increase the nucleus level of β-catenin and the p-GSK3β expression	Activation	Osteoporosis/Increase the DEX-induced reduction in BMC and microstructure parameters, and restore the levels of bone turnover markers in glucocorticoid-induced osteoporosis	^434^
Gentiopicroside	β-catenin	Increase β-catenin level	Activation	Osteoporosis/Promote BM-MSCs osteogenic differentiation, promote bone formation in OVX mice	^435^
Ahs	GSK3β	Bind to and inhibit GSK3β	Activation	Osteoporosis/Enhance osteoblast differentiation and bone formation, ameliorate prednisolone-induced osteoporosis	^436^
Troxerutin	Not clear	Increase the expression of β-catenin and downstream target genes of Wnt signaling	Activation	Bone fracture/Promote osteogenic differentiation of human BM-MSCs, stimulate new bone formation and accelerate the fracture healing in femur fracture rats	^437^
Small molecule inhibitors	Dvl-CXXC5 interaction	Inhibits Dvl-CXXC5 interaction	Activation	Osteoporosis/Enhance osteoblast differentiation, and rescue bone loss	^438^
Apigenin	Not clear	Increase the expression of β-catenin and downstream target genes of Wnt signaling	Activation	Bone fracture/Promote osteogenesis and facilitate the fracture healing	^439^
Glycyrrhizic acid (GA)	Not clear	Increase both active β-catenin and total β-catenin protein	Activation	Bone fracture/Promote osteogenic differentiation of human BM-MSCs and promote bone fracture healing	^440^
BM-MSCs-derived exosomal miR-335	VapB	Inhibit VapB expression	Activation	Bone fracture/Promote osteoblast differentiation and bone fracture recovery	^442^
BM-MSC-derived exosomes carrying miR-136-5p	LRP4	Inhibit LRP4 expression	Activation	Bone fracture/promote osteoblast proliferation and differentiation and fracture healing	^443^
Exosomes derived from platelet-rich plasma	Not clear	Reduce the protein levels of β-catenin, Runx2, and Wnt5a	Activation	OA/Promote proliferation and migration, inhibit apoptosis of OA chondrocyte and prevent OA progression	^444^
BM-MSC-derived exosomal miR-127-3p	CDH11	Inhibit CDH11	Inhibition	OA/Promote cell viability, suppresses apoptosis of OA chondrocyte and alleviates OA	^445^
PiR-63049	Wnt2b	Inhibit Wnt2b expression	Inhibition	Osteoporosis/PiR-63049-antagonist attenuates bone loss in OVX rats by promoting bone formation	^446^
miR-129-5p	TCF4	Inhibit TCF4 expression	Inhibition	Osteoporosis/Inhibition of miR-129-5p rescues osteoporosis	^448^
miR-320-3p	β-catenin	Inhibit the relative transcriptional activity of the β-catenin/TCF complex	Inhibition	OA/Injection of mmu-miR-320-3p attenuates OA progression in the OA mouse model	^449^

*AdDkk1* Dickkopf 1-expressing adenovirus, *BMC* bone mineral content, *BM-MSCs* bone marrow mesenchymal stem cells, *DEX* dexamethasone, *Dkk* Dickkopf, *Dkk1-AS* Dickkopf 1 antisense, *Fzd* frizzled, *GA* glycyrrhizic acid, *GSK3β* glycogen synthase kinase 3β, *hASCs* human adipose-derived stem cells, *LEF* lymphoid enhancer-binding factor, *LRP* low-density lipoprotein receptor-related protein, *MSCs* mesenchymal stem cells, *OA* osteoarthritis, *OCN* osteocalcin, *OPN* osteopontin, *OVX* ovariectomized, *PTH* parathyroid hormone, *RUNX2* runt-related transcription factor 2, *TCF* T-cell factor, *VapB* vesicle-associated membrane protein B, *WIF* Wnt inhibitory factor

### Targeting extracellular molecules of Wnt signaling

Targeting extracellular molecules taking part in Wnt signaling is of prime importance for treating bone diseases. To date, various biomolecules have been studied to target the extracellular molecules of the Wnt signaling pathway to treat bone disease.

Wnt ligands are attractive targets for treating bone diseases. Due to the promotion effect of Wnt signaling on bone mass, the addition of Wnts can improve osteoporosis by increasing bone mass. Yu et al. reported that Wnt4 prevents bone loss in osteoporosis by inhibiting NF-κB via non-canonical Wnt signaling.^398^ Jiang et al. suggested that stimulation using a pulsed electromagnetic field can activate the Wnt10b/LRP5/β⁃catenin pathway, which results in upregulation of Wnt10b, LRP5, β-catenin, OPG, and Runx2 and downregulation of Axin2, PPAR-γ, and Dkk-1 to prevent bone loss and improve lipid metabolism disorders in glucocorticoid-induced osteoporosis rats.^399^ In line with the findings of Jiang et al., Fan et al. also found that the application of electroacupuncture stimulation leads to increased expression of Wnt3α, β⁃catenin, and Runx2, which affects bone formation and promotes bone metabolism in rats with postmenopausal osteoporosis.^400^ More recently, Diegel et al. showed the significance of inhibiting Wnt secretion in alleviating high bone mass in three mouse models due to Sost loss-of-function and Lrp5 gain-of-function mutations.^401^ For OA treatment, Wnt pathway is also an attractive target. SM04690, a Wnt pathway inhibitor, appeared safe and well tolerated and showed disease-modifying OA drug properties for OA treatment in a phase 1 clinical trial (NCT02095548).^402^ Further phase 2 clinical trial of SM04690 (Lorecivint, LOR) for intra-articular therapy of moderate to severe knee OA showed that SM04690 improved the pain and cartilage degradation (NCT03122860).^403–405^ The involvement of Wnt5a produced by synoviocytes in RA suggests that suppression of Wnt5a is a potential treatment of RA.^406,407^ Wnt5a knockout mice were resistant to RA development, presenting as reduced inflammation parameters and less cartilage destruction.^406^ As Wnt5a promotes RA via ROCK signaling, ROCK inhibitor Y-27632 inhibits Wnt5a-induced RA FLS migration and reduced inflammatory cytokines IL-1β, IL-6, MMP3, MMP9 and MMP13 levels.^408^ Moreover, traditional Chinese medicine, such as Ginkgolide B and Resveratrol, show anti-RA effect for reducing articular cartilage and bone destruction and decreasing inflammatory cytokine levels through suppressing Wnt5a level.^409,410^ All these results suggest Wnt5a a critical drug target for treating RA (for review, see Huang et al.^381^). Liu et al. found that miR-21 overexpression inhibits the expression of IL-6 and IL-8 and relieves RA by suppressing Wnt expression.^411^ In addition, upregulation of Wnt16 through intra-articular injection of adenovirus-Wnt16 into mouse knee joint dramatically attenuated all the OA parameters.^357^ Fowler et al. designed an antibody-based platform to generate potent and selective Wnt mimetics and engineer bi-specific Wnt mimetics that target Fzd and LRPs.^412^ They found that the synthetic Wnt mimetics induce rapid and robust bone-building effects and that the Wnt mimetics correct bone mass deficiency and bone defects in various disease models, including osteoporosis, and long bone fracture.^412^ Additionally, these Wnt mimetics show improvement in the therapeutic effects of antiresorptive bisphosphonates and anti-sclerostin antibody.^412^ All these findings demonstrate Wnt mimetics as promising agents for treating bone disease. One recent study reveals a promising effect of a Wnt-induced osteogenic tissue model on maintaining the osteogenesis of human skeletal stem cells and repairing bone defects, demonstrating manipulation of Wnt signaling as promising strategy in treating bone disease.^413^

Secreted Wnt inhibitors/antagonists, such as Dkk1, sclerostin, WIF-1, and RSpo2, become attractive targets for the treatment of skeletal diseases. Exogenous end-capped phosphorothioate Dkk1-AS treatment significantly alleviates both estrogen depletion-induced bone loss in OVX rats and glucocorticoid-induced bone loss.^414,415^ Moreover, exosomal miR-196a from BM-MSCs significantly promotes osteoblast differentiation by targeting Dkk1 to activate the Wnt/β-catenin pathway, providing a novel therapeutic strategy for bone diseases such as osteoporosis.^416^ In human osteoblasts, miR-483-3p directly binds to and negatively regulates DKK2, an antagonist of Wnt signaling, thus increasing the expression of Wnt1, β-catenin, and cyclin D1. This increase in expression promotes the bone formation process by increasing osteoblast proliferation, pre-osteoblast differentiation into mature osteoblasts, and new bone matrix formation.^417^ Oh et al. found the upregulation of Dkk1 in both human and mouse experimental OA cartilage and showed that overexpression of Dkk1 by intraarticular injection of AdDkk1 significantly inhibits OA in mice, suggesting Dkk1 as a therapeutic target for OA treatment.^370^ However, Weng et al. found that Dkk1-AS treatment decreases the OA-associated increase of Dkk1 and abrogates chondrocyte apoptosis in OA in rats.^369^ These contrary findings may be due to different stages of OA progression and further clinical experiments are necessary.

The genetic linkage of sclerosteosis and van Buchem disease (two high bone mass diseases) to the *SOST* gene and the specificity of sclerostin in osteocytes strongly demonstrate that sclerostin is a target for osteoporosis therapy. Gao et al. demonstrated sclerostin as a target for enhancing the osteogenesis of BM-MSCs in the treatment of osteoporosis.^418^ They found that *SOST* overexpression significantly inhibited BM-MSCs proliferation and osteogenic differentiation, while Icariin promoted osteogenesis of BM-MSCs by regulating sclerostin, which activated Wnt/β-catenin signaling.^418^ Besides, a sclerostin antibody has been developed to improve bone mineral density. Anti-sclerostin antibody treatment significantly improves the bone quantity and quality of a Wnt1-related osteogenesis imperfecta mouse model.^304^ Presently, various studies that adopted animal models of human low bone mass diseases show the effectiveness and safety by targeting sclerostin for the treatment of osteoporosis, osteogenesis imperfecta, and osteoporosis pseudoglioma.^419^ Currently, romosozumab, a fully humanized monoclonal anti-sclerostin antibody, has been approved for the clinical application of treating osteoporosis in humans and shows efficacy in increasing BMD and reducing fragility fractures in both male and female osteoporotic patients.^420,421^ Phase 2 and phase 3 clinical trials (NCT00896532, NCT01575834, NCT01796301) show that romosozumab increases bone mineral density and bone formation, decreases bone resorption and reduces fracture risk in both postmenopausal women with osteoporosis and men with osteoporosis^422–426^ (for review, see Sølling et al.^421^ and Kerschan-Schindl^420^). More recently, sclerostin small-molecule inhibitors induced de novo bone to promote bone fusion, showing the potential to be used in novel, cost-effective bone graft substitutes for bone fusion and fracture defects healing.^427^ In addition, a bispecific antibody against both sclerostin and Dkk1 shows superior bone repair activity compared with monotherapies.^428^ Further, Choi et al. found that a sclerostin antibody/Dkk1 antibody combination approach was highly efficacious in the cancellous bone mass, suggesting that the osteoanabolic effects of Wnt pathway targeting can be made more efficient if multiple antagonists are simultaneously targeted.^429^

Zhu et al. showed that overexpression of WIF-1 promotes proliferation and suppresses apoptosis of OA chondrocytes by eliminating ROS and reducing the secretion of MMPs via blocking the Wnt/β-catenin signaling pathway, providing a new therapeutic theory for OA treatment.^372^ Melnik et al. demonstrated that miR-181a targets RSpo2, which is the activator of Wnt signaling and repressor of BMP signaling, to promote chondrogenesis of MSC.^430^ Moreover, they observed the disruption of a tight correlation between miR-181a and miR-218 expression levels in OA cartilage, highlighting the importance of the Wnt-BMP signaling crosstalk for preventing OA.^430^

The combination of these findings suggests that targeting either components or regulators of the Wnt signaling pathway could provide anabolic treatments for bone diseases.

### Targeting cytosol components of Wnt signaling

Cytosolic components of Wnt signaling like GSK3β, Dvl, and APC, and its modulators, such as MACF1, also play significant roles in targeting agents involved in treating bone disorders.

GSK3β, a cytosolic component of Wnt signaling, shows promise as a potential treatment target for treating various bone disorders. The inhibition of GSK3β from phosphorylating β-catenin through using lithium, which results in osteogenic differentiation and increasing bone mass in mouse models.^431,432^ Moreover, we demonstrated MACF1, the activator of Wnt/β-catenin signaling, as a promotor for osteoblast differentiation and bone formation^125,126,276^ and a novel potential therapeutic target for treating osteoporosis.^433^ Overexpression of MACF1 specifically in mesenchymal stem cells prevented aging-induced osteoporosis in 18- and 21-month-old mice.^433^

Small molecules cause great attention for their convenient application in the treatment of disease. Some small molecules show therapeutic effects on bone disease by targeting Wnt/β-catenin signaling. Wang et al. discovered that Daphnetin, a major active component of *daphne odora var. marginatai*, increases the dexamethasone (DEX)-induced reduction in bone mineral content (BMC) and microstructure parameters, and restores the levels of bone turnover markers in glucocorticoid-induced osteoporosis in vivo.^434^ Additionally, they found that Daphnetin promotes proliferation, differentiation, and mineralization in DEX-treated pre-osteoblasts in vitro and showed that Daphnetin activates Wnt/GSK3β/β-catenin signaling.^434^ Their findings demonstrate the potential therapeutic effect of Daphnetin on osteoporosis by targeting Wnt/GSK3β/β-catenin signaling.^434^ Gentiopicroside, a class of natural compounds, promotes BM-MSC osteogenesis by regulating the β-catenin-BMP signaling pathway both in vitro and in vivo.^435^ As silencing of β-catenin blocks the osteogenic differentiation induced by Gentiopicroside in BM-MSCs, β-catenin was revealed as the target for Gentiopicroside.^435^ The recent increase in understanding of Gentiopicroside may provide a novel strategy for the treatment of osteoporosis.^435^ Anthocyanin-enriched polyphenols from the petal of *H. syriacus L*. (Ahs) enhance osteoblast differentiation and bone formation both in vitro and in vivo while ameliorating prednisolone-induced osteoporosis.^436^ Ahs is able to bind to GSK3β and exerts the promotional effect on osteogenic activities by inhibiting GSK3β and subsequently activating β-catenin, leading to anti-osteoporosis.^436^ Troxerutin, a semi-synthetic derivative of the natural bioflavonoid rutin, enhances osteogenic differentiation of human BM-MSCs by stimulating the expression of the critical transcription factor β-catenin and several downstream target genes of Wnt signaling, such as Cmyc, CD44, and Survivin, thus activating Wnt/β-catenin signaling.^437^ Besides, Dvl-CXXC5 interaction is targeted for treating osteoporosis. CXXC5 is a negative feedback regulator of Wnt/β-catenin signaling through interacting with Dvl. By targeting Dvl-CXXC5 interaction, small molecules activate Wnt/β-catenin signaling, enhance osteoblast differentiation, and rescue bone loss in OVX mouse by inhibiting Dvl-CXXC5 interaction,^438^ demonstrating that targeting Dvl-CXXC5 interaction is a new strategy for treating osteoporosis. Furthermore, troxerutin stimulates new bone formation and accelerates the healing of femur fractures in rats.^437^ Apigenin, a natural plant flavone, promotes osteogenesis in vitro and facilitates the healing of fractures in vivo by enhancing β-catenin expression and activating Wnt/β-catenin signaling, indicating that Apigenin is a promising therapeutic candidate for bone fracture repair.^439^ Bai et al. found that glycyrrhizic acid (GA), a major triterpene glycoside isolated from licorice root, promotes osteogenic differentiation of human BM-MSCs by increasing both active β-catenin and total β-catenin protein, with GA-GelMA hydrogels promoting bone fracture healing, demonstrating GA as a potential and cost-effective treatment of bone defects.^440^

Recent findings demonstrate exosomes and noncoding RNAs as novel strategies for bone disease treatment. BM-MSCs-derived exosomes overexpressing miR-424-5p suppress osteogenesis by regulating the WIF-1/Wnt/β-catenin signaling pathway, demonstrating miR-424-5p as a new biomarker for the treatment of osteoporosis.^441^ BM-MSCs-derived exosomal miR-335 promotes osteoblast differentiation and bone fracture recovery via activating the Wnt/β-catenin pathway by targeting VapB (vesicle-associated membrane protein B), which is a regulator of vesicle trafficking. This finding provides a novel insight into therapeutic approaches in bone fracture treatment.^442^ BM-MSC-derived exosomes carrying miR-136-5p target and inhibit LRP4 expression to activate the Wnt/β-catenin pathway, thus promoting osteoblast proliferation and differentiation and initiating the healing of fractures.^443^ Exosomes derived from platelet-rich plasma (PRP-Exos) promote proliferation and migration, inhibit apoptosis of OA chondrocytes and prevent OA progression by activating the Wnt/β-catenin signaling pathway.^444^ Exosomal miR-127-3p derived from BM-MSCs promotes cell viability, suppresses apoptosis of OA chondrocyte, and alleviates OA by inhibiting CDH11, thereby blocking the Wnt/β-catenin pathway activation.^445^ Chen et al. found that piRNA-63049 is significantly increased in both bone tissues and plasma of osteoporotic rats and postmenopausal osteoporotic patients.^446^ Overexpression of piR-63049 inhibits osteoblastogenesis of BM-MSCs while knockdown of piR-63049 promotes the osteoblastogenesis of BM-MSCs through the upregulation of the Wnt2b/β-catenin signaling pathway.^446^ PiR-63049-antagonist can attenuate bone loss in OVX rats by promoting bone formation, suggesting piR-63049 as a possible novel target for treating osteoporosis.^446^

### Targeting the nuclear components of Wnt signaling

Like extracellular molecules and cytosolic components, the nuclear components of Wnt signaling can also be targeted for treating bone diseases. As a key mediator of Wnt/β-catenin signaling, β-catenin translocates into the nucleus to bind to TCF/LEF transcription factors that regulate the transcription of the downstream target genes. Therefore, targeting β-catenin, TCF/LEF, or the interaction of β-catenin and TCF/LEF in the nucleus is an opportunity for disease therapy.

MicroRNAs show possible therapeutic potential by targeting the nuclear components of Wnt signaling. Let-7i-3p negatively regulates the Wnt/β-catenin signaling pathway by targeting LEF1 and inhibiting osteogenic differentiation of human adipose-derived stem cells (hASCs) under cyclic strain in vitro.^447^ Therefore, either inhibition of Let-7i-3p or overexpression of LEF1 promotes osteogenic differentiation of hASCs,^447^ demonstrating the targeting of LEF1 as a therapeutic strategy for treating disease. Moreover, miR-129-5p targets TCF4 to inhibit Wnt/β-catenin signaling, thus inhibiting osteoblast differentiation and bone formation.^448^ Thus, inhibition of miR-129-5p enhances osteoblast differentiation and bone formation, showing a rescue effect on osteoporosis.^448^ Recently, Hu et al. demonstrated that miR-320c can inhibit chondrogenic degeneration during OA by downregulating the β-catenin protein level in the nucleus and decreasing the relative transcriptional activity of the β-catenin/TCF complex.^449^ Intra-articular injection of mmu-miR-320-3p attenuates OA progression in the OA mouse model, demonstrating miR-320-3p as a novel therapeutic agent for OA treatment.^449^

Taken together, from the extracellular components, cytosol components to the nuclear components of the Wnt signaling pathway, multiple components have been studied as drug targets to modulate Wnt signaling, making Wnt signaling a perfect target for treating bone diseases.

## Conclusions and perspectives

Wnt signaling is a universal signaling pathway involved in development, physiology, and pathology. Here we highlight data exploring the role of both canonical (Wnt/β-catenin) and noncanonical Wnt signaling pathways in bone physiology and pathology by discussing Wnt proteins, receptors, activators, and inhibitors, and their interactions. Moreover, the efforts targeting Wnt signaling for treating bone disease are summarized. Human bone diseases and skeletal abnormalities resulting from aberrant Wnt signaling mimicked in mutant mice reveal the importance of Wnt signaling in bone development. Different animal models and human diseases studies establish a complex Wnt signaling pathway network with multiple players. Defects in Wnt ligands and agonists may lead to bone development disorders, joint formation abnormality, or osteoporosis.^450^ Mutation in LRP5/6, the Wnts receptors, leads to various bone diseases. Sclerostin is an extracellular antagonist that is involved in several bone diseases, including van Buchem disease and sclerosteosis. The mutation of other inhibitors such as WIF and Dkk causes altered bone density. In addition, the Wnt signaling pathway overlaps with other pathways related to bone development such as the PTH pathway, the Ihh pathway, and the TGF-β/BMP pathway. Through this interconnected network of signaling pathways, Wnt signaling regulates both bone remodeling and the determination of mesenchymal stem cell fate.

The Wnt signaling pathway has been studied for decades, however, many important questions regarding Wnt signaling remain unanswered. What are the molecular structures of Wnt pathway components? What is their mechanism of interaction, the complicated network between the canonical Wnt pathway, noncanonical Wnt pathway, and other pathways? Where does Wnt signaling take place in cell organelles? What is the insight into the mechanisms of action of these Wnt receptors? Can we identify a truly potent Wnt inhibitor or agonist? Whether further complexity of Wnt-regulated gene expression will be uncovered through comparative analyses of Wnt-responsive transcription programs that depend on TCF/LEF versus others?

As Wnt signaling plays an important role in bone formation, homeostasis and diseases, it become the topic of drug development for bone diseases. The approach to the Wnt pathway focuses on extracellular mediators such as Sclerostin, which is selectively and highly expressed in bone. Agents that specifically target Sclerostin show great promise for simultaneously treating osteoporosis and repairing bone fractures. The FDA approved the first-in-class anti-sclerostin antibody for osteoporosis in 2019. While current osteoporosis drugs decrease bone breakdown and bone formation, the anti-sclerostin antibody simultaneously increases bone formation and decreases bone breakdown.^451^ The Wnt signaling pathway consists of numerous antagonists, ligands, and intracellular proteins that alter the development of bone as well as the pathogenesis of bone diseases. Thus, it would be useful to investigate more upstream targets in the Wnt canonical pathway for further drug discovery, which could yield more promise than targeting β-catenin and downstream events.

New biological analytical technologies, including single-cell RNA-seq analysis and spatial transcriptomics should enable us to dissect where, when, and how Wnt signaling occurs inside the cells. New imaging technologies, such as spatially resolved, highly multiplexed RNA profiling in single cells^452^ will facilitate the visualization of the dynamic Wnt signaling events in vivo. Novel regulators will likely continue to be identified using classical genetic, molecular, modern genomic, proteomic approaches, bioinformatics, and protein structure biology using cryo-electron microscopy and Deep learning system, AlphaFold, an artificial intelligence (AI) program, which performs predictions of protein structure. As there are multiple components in Wnt signaling, identification of the key regulator or the key interaction between these components involved in specific tissue physiology or disease will be helpful for both understanding the underlying mechanism and providing a target for specific disease therapy. Besides, it will be helpful to identify specific activators and inhibitors of the specific components of Wnt signaling based on their structure, both for studying their physio-pathological role and for investigating therapeutic methods. Answering these questions and identifying these issues will provide a deeper understanding of the physiological and pathological role of Wnt signaling and will make Wnt signaling a more suitable target for bone disease therapy.

## References

[1] Steinhart Z, Angers S (2018). Wnt signaling in development and tissue homeostasis. Development.

[2] Nusse R, Clevers H (2017). Wnt/β-catenin signaling, disease, and emerging therapeutic modalities. Cell.

[3] Nusse R (1991). A new nomenclature for int-1 and related genes: the Wnt gene family. Cell.

[4] Nusse R, Varmus HE (1982). Many tumors induced by the mouse mammary tumor virus contain a provirus integrated in the same region of the host genome. Cell.

[5] Nusslein-Volhard C, Wieschaus E (1980). Mutations affecting segment number and polarity in Drosophila. Nature.

[6] Rijsewijk F (1987). The Drosophila homolog of the mouse mammary oncogene int-1 is identical to the segment polarity gene wingless. Cell.

[7] Siegfried E, Chou TB, Perrimon N (1992). wingless signaling acts through zeste-white 3, the Drosophila homolog of glycogen synthase kinase-3, to regulate engrailed and establish cell fate. Cell.

[8] Noordermeer J, Klingensmith J, Perrimon N, Nusse R (1994). dishevelled and armadillo act in the wingless signalling pathway in Drosophila. Nature.

[9] Peifer M, Sweeton D, Casey M, Wieschaus E (1994). Wingless signal and Zeste-white 3 kinase trigger opposing changes in the intracellular distribution of Armadillo. Development.

[10] McMahon AP, Moon RT (1989). Ectopic expression of the proto-oncogene int-1 in Xenopus embryos leads to duplication of the embryonic axis. Cell.

[11] Munoz-Descalzo S, Hadjantonakis AK, Arias AM (2015). Wnt/ß-catenin signalling and the dynamics of fate decisions in early mouse embryos and embryonic stem (ES) cells. Semin. Cell Dev. Biol..

[12] Rudnicki MA, Williams BO (2015). Wnt signaling in bone and muscle. Bone.

[13] Lojk, J. & Marc, J. Roles of non-canonical wnt signalling pathways in bone biology. *Int. J. Mol. Sci.* **22** (2021). 10.3390/ijms221910840 PMC8509327 34639180

[14] Kinzler KW (1991). Identification of FAP locus genes from chromosome 5q21. Science.

[15] Nishisho I (1991). Mutations of chromosome 5q21 genes in FAP and colorectal cancer patients. Science.

[16] Rubinfeld B (1993). Association of the APC gene product with beta-catenin. Science.

[17] Su LK, Vogelstein B, Kinzler KW (1993). Association of the APC tumor suppressor protein with catenins. Science.

[18] Korinek V (1997). Constitutive transcriptional activation by a beta-catenin-Tcf complex in *APC*^*−/−*^ colon carcinoma. Science.

[19] Clevers H, Nusse R (2012). Wnt/β-catenin signaling and disease. Cell.

[20] Ackers I, Malgor R (2018). Interrelationship of canonical and non-canonical Wnt signalling pathways in chronic metabolic diseases. Diabetes Vasc. Dis. Res..

[21] Krishnamurthy N, Kurzrock R (2018). Targeting the Wnt/beta-catenin pathway in cancer: Update on effectors and inhibitors. Cancer Treat. Rev..

[22] Miller JR (2002). The Wnts. Genome Biol..

[23] Willert K (2003). Wnt proteins are lipid-modified and can act as stem cell growth factors. Nature.

[24] Takada R (2006). Monounsaturated fatty acid modification of Wnt protein: its role in Wnt secretion. Dev. Cell.

[25] Kurayoshi M, Yamamoto H, Izumi S, Kikuchi A (2007). Post-translational palmitoylation and glycosylation of Wnt-5a are necessary for its signalling. Biochem. J..

[26] Du SJ (1995). Identification of distinct classes and functional domains of Wnts through expression of wild-type and chimeric proteins in Xenopus embryos. Mol. Cell Biol..

[27] Tao Q (2005). Maternal wnt11 activates the canonical wnt signaling pathway required for axis formation in Xenopus embryos. Cell.

[28] Toyama T (2010). Noncanonical Wnt11 inhibits hepatocellular carcinoma cell proliferation and migration. Mol. Cancer Res..

[29] Tian S (2018). Secreted AGR2 promotes invasion of colorectal cancer cells via Wnt11-mediated non-canonical Wnt signaling. Exp. Cell Res..

[30] Mikels AJ, Nusse R (2006). Wnts as ligands: processing, secretion and reception. Oncogene.

[31] Hsieh JC, Rattner A, Smallwood PM, Nathans J (1999). Biochemical characterization of Wnt-frizzled interactions using a soluble, biologically active vertebrate Wnt protein. Proc. Natl. Acad. Sci. USA.

[32] Tamai K (2000). LDL-receptor-related proteins in Wnt signal transduction. Nature.

[33] Grumolato L (2010). Canonical and noncanonical Wnts use a common mechanism to activate completely unrelated coreceptors. Genes Dev..

[34] Bonkowsky JL (1999). Axon routing across the midline controlled by the Drosophila Derailed receptor. Nature.

[35] Lu W, Yamamoto V, Ortega B, Baltimore D (2004). Mammalian Ryk is a Wnt coreceptor required for stimulation of neurite outgrowth. Cell.

[36] Peradziryi H, Tolwinski NS, Borchers A (2012). The many roles of PTK7: a versatile regulator of cell-cell communication. Arch. Biochem. Biophys..

[37] Masiakowski P, Yancopoulos GD (1998). The Wnt receptor CRD domain is also found in MuSK and related orphan receptor tyrosine kinases. Curr. Biol..

[38] Bhanot P (1996). A new member of the frizzled family from Drosophila functions as a Wingless receptor. Nature.

[39] MacDonald, B. T. & He, X. Frizzled and LRP5/6 receptors for Wnt/beta-catenin signaling. *Cold Spring Harb. Perspect. Biol.* **4** a007880 (2012). 10.1101/cshperspect.a007880 PMC3504444 23209147

[40] Dann CE (2001). Insights into Wnt binding and signalling from the structures of two Frizzled cysteine-rich domains. Nature.

[41] Janda CY (2012). Structural basis of Wnt recognition by Frizzled. Science.

[42] Agostino M, Pohl, S O, Dharmarajan A (2017). Structure-based prediction of Wnt binding affinities for Frizzled-type cysteine-rich domains. J. Biol. Chem..

[43] Nishita M (2010). Ror2/frizzled complex mediates Wnt5a-induced AP-1 activation by regulating Dishevelled polymerization. Mol. Cell Biol..

[44] Wehrli M (2000). arrow encodes an LDL-receptor-related protein essential for Wingless signalling. Nature.

[45] Pinson KI (2000). An LDL-receptor-related protein mediates Wnt signalling in mice. Nature.

[46] Houston DW, Wylie C (2002). Cloning and expression of Xenopus Lrp5 and Lrp6 genes. Mech. Dev..

[47] Kelly OG, Pinson KI, Skarnes WC (2004). The Wnt co-receptors Lrp5 and Lrp6 are essential for gastrulation in mice. Development.

[48] Mao J (2001). Low-density lipoprotein receptor-related protein-5 binds to Axin and regulates the canonical Wnt signaling pathway. Mol. Cell.

[49] Bourhis E (2010). Reconstitution of a frizzled8.Wnt3a.LRP6 signaling complex reveals multiple Wnt and Dkk1 binding sites on LRP6. J. Biol. Chem..

[50] Minami Y, Oishi I, Endo M, Nishita M (2010). Ror-family receptor tyrosine kinases in noncanonical Wnt signaling: their implications in developmental morphogenesis and human diseases. Dev. Dyn..

[51] Fukuda T (2008). Antisera induced by infusions of autologous Ad-CD154-leukemia B cells identify ROR1 as an oncofetal antigen and receptor for Wnt5a. Proc. Natl. Acad. Sci. USA.

[52] Oishi I (2003). The receptor tyrosine kinase Ror2 is involved in non-canonical Wnt5a/JNK signalling pathway. Genes Cells.

[53] Hikasa H, Shibata M, Hiratani I, Taira M (2002). The Xenopus receptor tyrosine kinase Xror2 modulates morphogenetic movements of the axial mesoderm and neuroectoderm via Wnt signaling. Development.

[54] Yu J (2016). Wnt5a induces ROR1/ROR2 heterooligomerization to enhance leukemia chemotaxis and proliferation. J. Clin. Investig..

[55] Ho HY (2012). Wnt5a-Ror-Dishevelled signaling constitutes a core developmental pathway that controls tissue morphogenesis. Proc. Natl. Acad. Sci. USA.

[56] Qi X, Okinaka Y, Nishita M, Minami Y (2016). Essential role of Wnt5a-Ror1/Ror2 signaling in metanephric mesenchyme and ureteric bud formation. Genes Cells.

[57] Wu X, Yan T, Hao L, Zhu Y (2019). Wnt5a induces ROR1 and ROR2 to activate RhoA in esophageal squamous cell carcinoma cells. Cancer Manag Res..

[58] Mikels AJ, Nusse R (2006). Purified Wnt5a protein activates or inhibits beta-catenin-TCF signaling depending on receptor context. PLoS Biol..

[59] Hovens CM (1992). RYK, a receptor tyrosine kinase-related molecule with unusual kinase domain motifs. Proc. Natl. Acad. Sci. USA.

[60] Halford MM, Stacker SA (2001). Revelations of the RYK receptor. Bioessays.

[61] Mossie K (1995). Colon carcinoma kinase-4 defines a new subclass of the receptor tyrosine kinase family. Oncogene.

[62] Shnitsar I, Borchers A (2008). PTK7 recruits dsh to regulate neural crest migration. Development.

[63] Puppo F (2011). Protein tyrosine kinase 7 has a conserved role in Wnt/beta-catenin canonical signalling. EMBO Rep..

[64] Peradziryi H (2011). PTK7/Otk interacts with Wnts and inhibits canonical Wnt signalling. EMBO J..

[65] Bin-Nun N (2014). PTK7 modulates Wnt signaling activity via LRP6. Development.

[66] Martinez S (2015). The PTK7 and ROR2 protein receptors interact in the vertebrate WNT/Planar Cell Polarity (PCP) pathway. J. Biol. Chem..

[67] Berger H, Wodarz A, Borchers A (2017). PTK7 faces the Wnt in development and disease. Front Cell Dev. Biol..

[68] Hayes M (2013). Ptk7 promotes non-canonical Wnt/PCP-mediated morphogenesis and inhibits Wnt/beta-catenin-dependent cell fate decisions during vertebrate development. Development.

[69] DeChiara TM (1996). The receptor tyrosine kinase MuSK is required for neuromuscular junction formation in vivo. Cell.

[70] Glass DJ (1996). Agrin acts via a MuSK receptor complex. Cell.

[71] Gordon LR, Gribble KD, Syrett CM, Granato M (2012). Initiation of synapse formation by Wnt-induced MuSK endocytosis. Development.

[72] Strochlic L (2012). Wnt4 participates in the formation of vertebrate neuromuscular junction. PLoS One.

[73] Zhang B (2012). Wnt proteins regulate acetylcholine receptor clustering in muscle cells. Mol. Brain.

[74] Angers S, Moon RT (2009). Proximal events in Wnt signal transduction. Nat. Rev. Mol. Cell Biol..

[75] Bilic J (2007). Wnt induces LRP6 signalosomes and promotes dishevelled-dependent LRP6 phosphorylation. Science.

[76] Niehrs C, Shen J (2010). Regulation of Lrp6 phosphorylation. Cell Mol. Life Sci..

[77] Devenport D, Fuchs E (2008). Planar polarization in embryonic epidermis orchestrates global asymmetric morphogenesis of hair follicles. Nat. Cell Biol..

[78] Yuan K (2015). Activation of the Wnt/planar cell polarity pathway is required for pericyte recruitment during pulmonary angiogenesis. Am. J. Pathol..

[79] Lopez-Escobar, B. et al. The non-canonical Wnt-PCP pathway shapes the mouse caudal neural plate. *Development* **145**, dev157487 (2018). 10.1242/dev.157487 PMC5992595 29636380

[80] Huang YL, Niehrs C (2014). Polarized Wnt signaling regulates ectodermal cell fate in Xenopus. Dev. Cell.

[81] Andre P (2012). The Wnt coreceptor Ryk regulates Wnt/planar cell polarity by modulating the degradation of the core planar cell polarity component Vangl2. J. Biol. Chem..

[82] Sakane H (2012). Localization of glypican-4 in different membrane microdomains is involved in the regulation of Wnt signaling. J. Cell Sci..

[83] Carvallo L (2010). Non-canonical Wnt signaling induces ubiquitination and degradation of Syndecan4. J. Biol. Chem..

[84] Sebbagh M, Borg JP (2014). Insight into planar cell polarity. Exp. Cell Res..

[85] Kuhl M (2000). The Wnt/Ca^2+^ pathway: a new vertebrate Wnt signaling pathway takes shape. Trends Genet..

[86] Anakwe K (2003). Wnt signalling regulates myogenic differentiation in the developing avian wing. Development.

[87] Saneyoshi T, Kume S, Amasaki Y, Mikoshiba K (2002). The Wnt/calcium pathway activates NF-AT and promotes ventral cell fate in Xenopus embryos. Nature.

[88] Undi RB (2016). Wnt signaling: role in regulation of haematopoiesis. Indian J. Hematol. Blood Transfus..

[89] McQuate A, Latorre-Esteves E, Barria A (2017). A Wnt/calcium signaling cascade regulates neuronal excitability and trafficking of NMDARs. Cell Rep..

[90] De A (2011). Wnt/Ca^2+^ signaling pathway: a brief overview. Acta Biochim. Biophys. Sin..

[91] Cao X (2021). WNT10A induces apoptosis of senescent synovial resident stem cells through Wnt/calcium pathway-mediated HDAC5 phosphorylation in OA joints. Bone.

[92] Zhen H (2020). The Wnt/Ca^2+^ signaling pathway is essential for the regeneration of GABAergic neurons in planarian Dugesia japonica. Faseb J..

[93] Semenov MV, Habas R, Macdonald BT, He X (2007). SnapShot: noncanonical Wnt signaling pathways. Cell.

[94] Thrasivoulou C, Millar M, Ahmed A (2013). Activation of intracellular calcium by multiple Wnt ligands and translocation of beta-catenin into the nucleus: a convergent model of Wnt/Ca^2+^ and Wnt/beta-catenin pathways. J. Biol. Chem..

[95] Wu S, Yu Q, Lai A, Tian J (2018). Pulsed electromagnetic field induces Ca^2+^-dependent osteoblastogenesis in C3H10T1/2 mesenchymal cells through the Wnt-Ca^2+^/Wnt-beta-catenin signaling pathway. Biochem. Biophys. Res. Commun..

[96] Gao C, Chen YG (2010). Dishevelled: the hub of Wnt signaling. Cell Signal.

[97] Jin YR, Yoon JK (2012). The R-spondin family of proteins: emerging regulators of WNT signaling. Int. J. Biochem. Cell Biol..

[98] Xu Q (2004). Vascular development in the retina and inner ear: control by Norrin and Frizzled-4, a high-affinity ligand-receptor pair. Cell.

[99] Braunger BM (2013). Constitutive overexpression of Norrin activates Wnt/beta-catenin and endothelin-2 signaling to protect photoreceptors from light damage. Neurobiol. Dis..

[100] Ke J (2013). Structure and function of Norrin in assembly and activation of a Frizzled 4-Lrp5/6 complex. Genes Dev..

[101] Hu L (2017). MACF1, versatility in tissue-specific function and in human disease. Semin. Cell Dev. Biol..

[102] Chen HJ (2006). The role of microtubule actin cross-linking factor 1 (MACF1) in the Wnt signaling pathway. Genes Dev..

[103] Moparthi L, Pizzolato G, Koch S (2019). Wnt activator FOXB2 drives the neuroendocrine differentiation of prostate cancer. Proc. Natl. Acad. Sci. USA.

[104] He Z (2023). R-spondin family biology and emerging linkages to cancer. Ann. Med..

[105] Binnerts ME (2007). R-Spondin1 regulates Wnt signaling by inhibiting internalization of LRP6. Proc. Natl. Acad. Sci. USA.

[106] Kim KA (2008). R-Spondin family members regulate the Wnt pathway by a common mechanism. Mol. Biol. Cell.

[107] Nam JS (2006). Mouse cristin/R-spondin family proteins are novel ligands for the Frizzled 8 and LRP6 receptors and activate beta-catenin-dependent gene expression. J. Biol. Chem..

[108] Wei Q (2007). R-spondin1 is a high-affinity ligand for LRP6 and induces LRP6 phosphorylation and beta-catenin signaling. J. Biol. Chem..

[109] Carmon KS (2011). R-spondins function as ligands of the orphan receptors LGR4 and LGR5 to regulate Wnt/beta-catenin signaling. Proc. Natl. Acad. Sci. USA.

[110] de Lau W (2011). Lgr5 homologues associate with Wnt receptors and mediate R-spondin signalling. Nature.

[111] de Lau W, Peng WC, Gros P, Clevers H (2014). The R-spondin/Lgr5/Rnf43 module: regulator of Wnt signal strength. Genes Dev..

[112] Wang D (2013). Structural basis for R-spondin recognition by LGR4/5/6 receptors. Genes Dev..

[113] Ohkawara B, Glinka A, Niehrs C (2011). Rspo3 binds syndecan 4 and induces Wnt/PCP signaling via clathrin-mediated endocytosis to promote morphogenesis. Dev. Cell.

[114] Lacour F (2017). R-spondin1 controls muscle cell fusion through dual regulation of antagonistic Wnt signaling pathways. Cell Rep..

[115] Dong X (2017). RSPO2 suppresses colorectal cancer metastasis by counteracting the Wnt5a/Fzd7-driven noncanonical Wnt pathway. Cancer Lett..

[116] Scholz B (2016). Endothelial RSPO3 controls vascular stability and pruning through non-canonical WNT/Ca^2+^/NFAT signaling. Dev. Cell.

[117] Ye X (2009). Norrin, frizzled-4, and Lrp5 signaling in endothelial cells controls a genetic program for retinal vascularization. Cell.

[118] Smallwood PM (2007). Mutational analysis of Norrin-Frizzled4 recognition. J. Biol. Chem..

[119] Chang, T. H. et al. Structure and functional properties of Norrin mimic Wnt for signalling with Frizzled4, Lrp5/6, and proteoglycan. *Elife* **4**, e06554 (2015). 10.7554/eLife.06554 PMC4497409 26158506

[120] Bang I (2018). Biophysical and functional characterization of Norrin signaling through Frizzled4. Proc. Natl. Acad. Sci. USA.

[121] Byers TJ, Beggs AH, McNally EM, Kunkel LM (1995). Novel actin crosslinker superfamily member identified by a two step degenerate PCR procedure. FEBS Lett..

[122] Leung CL (1999). Microtubule actin cross-linking factor (MACF): a hybrid of dystonin and dystrophin that can interact with the actin and microtubule cytoskeletons. J. Cell Biol..

[123] Hu L (2016). Isoforms, structures, and functions of versatile spectraplakin MACF1. BMB Rep..

[124] Liu P (1999). Requirement for Wnt3 in vertebrate axis formation. Nat. Genet..

[125] Hu L (2018). Microtubule actin crosslinking factor 1 promotes osteoblast differentiation by promoting β-catenin/TCF1/Runx2 signaling axis. J. Cell Physiol..

[126] Zhang Y (2018). MACF1 overexpression by transfecting the 21 kbp large plasmid PEGFP-C1A-ACF7 promotes osteoblast differentiation and bone formation. Hum. Gene Ther..

[127] Cruciat CM, Niehrs C (2013). Secreted and transmembrane wnt inhibitors and activators. Cold Spring Harb. Perspect. Biol..

[128] Fedi P (1999). Isolation and biochemical characterization of the human Dkk-1 homologue, a novel inhibitor of mammalian Wnt signaling. J. Biol. Chem..

[129] Mao B (2001). LDL-receptor-related protein 6 is a receptor for Dickkopf proteins. Nature.

[130] Galli LM (2006). Differential inhibition of Wnt-3a by Sfrp-1, Sfrp-2, and Sfrp-3. Dev. Dyn..

[131] Malinauskas T (2011). Modular mechanism of Wnt signaling inhibition by Wnt inhibitory factor 1. Nat. Struct. Mol. Biol..

[132] Itasaki N (2003). Wise, a context-dependent activator and inhibitor of Wnt signalling. Development.

[133] Li X (2005). Sclerostin binds to LRP5/6 and antagonizes canonical Wnt signaling. J. Biol. Chem..

[134] Zhu W (2008). IGFBP-4 is an inhibitor of canonical Wnt signalling required for cardiogenesis. Nature.

[135] Piccolo S (1999). The head inducer Cerberus is a multifunctional antagonist of Nodal, BMP and Wnt signals. Nature.

[136] Ding Y (2018). Bighead is a Wnt antagonist secreted by the Xenopus Spemann organizer that promotes Lrp6 endocytosis. Proc. Natl. Acad. Sci. USA.

[137] Yamamoto A (2005). Shisa promotes head formation through the inhibition of receptor protein maturation for the caudalizing factors, Wnt and FGF. Cell.

[138] Zhang X (2012). Tiki1 is required for head formation via Wnt cleavage-oxidation and inactivation. Cell.

[139] Kagermeier-Schenk B (2011). Waif1/5T4 inhibits Wnt/beta-catenin signaling and activates noncanonical Wnt pathways by modifying LRP6 subcellular localization. Dev. Cell.

[140] Shimomura Y (2010). APCDD1 is a novel Wnt inhibitor mutated in hereditary hypotrichosis simplex. Nature.

[141] Glinka A (1998). Dickkopf-1 is a member of a new family of secreted proteins and functions in head induction. Nature.

[142] Niehrs C (2006). Function and biological roles of the Dickkopf family of Wnt modulators. Oncogene.

[143] Bafico A (2001). Novel mechanism of Wnt signalling inhibition mediated by Dickkopf-1 interaction with LRP6/Arrow. Nat. Cell Biol..

[144] Patel S (2018). Structural and functional analysis of Dickkopf 4 (Dkk4): new insights into Dkk evolution and regulation of Wnt signaling by Dkk and Kremen proteins. J. Biol. Chem..

[145] Mao B (2002). Kremen proteins are Dickkopf receptors that regulate Wnt/beta-catenin signalling. Nature.

[146] Ellwanger K (2008). Targeted disruption of the Wnt regulator Kremen induces limb defects and high bone density. Mol. Cell Biol..

[147] Wu W, Glinka A, Delius H, Niehrs C (2000). Mutual antagonism between dickkopf1 and dickkopf2 regulates Wnt/beta-catenin signalling. Curr. Biol..

[148] Brott BK, Sokol SY (2002). Regulation of Wnt/LRP signaling by distinct domains of Dickkopf proteins. Mol. Cell Biol..

[149] Mao B, Niehrs C (2003). Kremen2 modulates Dickkopf2 activity during Wnt/LRP6 signaling. Gene.

[150] Zhang W (2023). Secreted frizzled-related proteins: a promising therapeutic target for cancer therapy through Wnt signaling inhibition. Biomed. Pharmacother..

[151] Hoang B, Moos M, Vukicevic S, Luyten FP (1996). Primary structure and tissue distribution of FRZB, a novel protein related to Drosophila frizzled, suggest a role in skeletal morphogenesis. J. Biol. Chem..

[152] Leyns L (1997). Frzb-1 is a secreted antagonist of Wnt signaling expressed in the Spemann organizer. Cell.

[153] Rattner A (1997). A family of secreted proteins contains homology to the cysteine-rich ligand-binding domain of frizzled receptors. Proc. Natl. Acad. Sci. USA.

[154] Li Y (2008). Sfrp5 coordinates foregut specification and morphogenesis by antagonizing both canonical and noncanonical Wnt11 signaling. Genes Dev..

[155] Satoh W (2008). Sfrp1, Sfrp2, and Sfrp5 regulate the Wnt/beta-catenin and the planar cell polarity pathways during early trunk formation in mouse. Genesis.

[156] Holly VL, Widen SA, Famulski JK, Waskiewicz AJ (2014). Sfrp1a and Sfrp5 function as positive regulators of Wnt and BMP signaling during early retinal development. Dev. Biol..

[157] Xavier CP (2014). Secreted Frizzled-related protein potentiation versus inhibition of Wnt3a/beta-catenin signaling. Cell Signal..

[158] Zhang J (2014). Wnt inhibitory factor-1 functions as a tumor suppressor through modulating Wnt/beta-catenin signaling in neuroblastoma. Cancer Lett..

[159] Vassallo I (2016). WIF1 re-expression in glioblastoma inhibits migration through attenuation of non-canonical WNT signaling by downregulating the lncRNA MALAT1. Oncogene.

[160] Surmann-Schmitt C (2009). Wif-1 is expressed at cartilage-mesenchyme interfaces and impedes Wnt3a-mediated inhibition of chondrogenesis. J. Cell Sci..

[161] Balemans W (2001). Increased bone density in sclerosteosis is due to the deficiency of a novel secreted protein (SOST). Hum. Mol. Genet..

[162] van Bezooijen RL (2004). Sclerostin is an osteocyte-expressed negative regulator of bone formation, but not a classical BMP antagonist. J. Exp. Med..

[163] Winkler DG (2003). Osteocyte control of bone formation via sclerostin, a novel BMP antagonist. EMBO J..

[164] Holdsworth G (2012). Characterization of the interaction of sclerostin with the low density lipoprotein receptor-related protein (LRP) family of Wnt co-receptors. J. Biol. Chem..

[165] Kim J (2020). Sclerostin inhibits Wnt signaling through tandem interaction with two LRP6 ectodomains. Nat. Commun..

[166] Leupin O (2011). Bone overgrowth-associated mutations in the LRP4 gene impair sclerostin facilitator function. J. Biol. Chem..

[167] Krause C (2010). Distinct modes of inhibition by sclerostin on bone morphogenetic protein and Wnt signaling pathways. J. Biol. Chem..

[168] Ahn Y, Sanderson BW, Klein OD, Krumlauf R (2010). Inhibition of Wnt signaling by Wise (Sostdc1) and negative feedback from Shh controls tooth number and patterning. Development.

[169] Ahn Y (2013). Lrp4 and Wise interplay controls the formation and patterning of mammary and other skin appendage placodes by modulating Wnt signaling. Development.

[170] Firth SM, Baxter RC (2002). Cellular actions of the insulin-like growth factor binding proteins. Endocr. Rev..

[171] Wo D (2016). Opposing roles of Wnt inhibitors IGFBP-4 and Dkk1 in cardiac ischemia by differential targeting of LRP5/6 and beta-catenin. Circulation.

[172] Ueno K (2011). IGFBP-4 activates the Wnt/beta-catenin signaling pathway and induces M-CAM expression in human renal cell carcinoma. Int. J. Cancer.

[173] Bouwmeester T (1996). Cerberus is a head-inducing secreted factor expressed in the anterior endoderm of Spemann’s organizer. Nature.

[174] Zhang Y-J, Shi D-L (2020). Diversification of amphioxus and vertebrate Cerberus protein function in modulating Nodal, BMP and Wnt signals. Mar. Life Sci. Technol..

[175] Belo JA (2009). Generating asymmetries in the early vertebrate embryo: the role of the Cerberus-like family. Int. J. Dev. Biol..

[176] Colozza, G. Purified Bighead protein efficiently promotes head development in the South African clawed frog, Xenopus laevis. *microPubl. Biol.* **2021**, 10.17912/micropub.biology.000347 (2021). 10.17912/micropub.biology.000347 PMC7786221 33426508

[177] Pei J, Grishin NV (2012). Unexpected diversity in Shisa-like proteins suggests the importance of their roles as transmembrane adaptors. Cell Signal.

[178] Nagano T (2006). Shisa2 promotes the maturation of somitic precursors and transition to the segmental fate in Xenopus embryos. Development.

[179] Furushima K (2007). Mouse homologues of Shisa antagonistic to Wnt and Fgf signalings. Dev. Biol..

[180] Zhao Y, Malinauskas T, Harlos K, Jones EY (2014). Structural insights into the inhibition of Wnt signaling by cancer antigen 5T4/Wnt-activated inhibitory factor 1. Structure.

[181] Mazzoni J (2017). The Wnt inhibitor Apcdd1 coordinates vascular remodeling and barrier maturation of retinal blood vessels. Neuron.

[182] Yiew NKH (2017). A novel role for the Wnt inhibitor APCDD1 in adipocyte differentiation: Implications for diet-induced obesity. J. Biol. Chem..

[183] Viale-Bouroncle S, Klingelhoffer C, Ettl T, Morsczeck C (2015). The WNT inhibitor APCDD1 sustains the expression of beta-catenin during the osteogenic differentiation of human dental follicle cells. Biochem. Biophys. Res. Commun..

[184] Hayward P, Kalmar T, Arias AM (2008). Wnt/Notch signalling and information processing during development. Development.

[185] Collu GM, Hidalgo-Sastre A, Brennan K (2014). Wnt-Notch signalling crosstalk in development and disease. Cell Mol. Life Sci..

[186] Gao J, Fan L, Zhao L, Su Y (2021). The interaction of Notch and Wnt signaling pathways in vertebrate regeneration. Cell Regen..

[187] Estrach S (2006). Jagged 1 is a beta-catenin target gene required for ectopic hair follicle formation in adult epidermis. Development.

[188] Pannequin J (2009). The wnt target jagged-1 mediates the activation of notch signaling by progastrin in human colorectal cancer cells. Cancer Res..

[189] Kwon C (2011). Notch post-translationally regulates β-catenin protein in stem and progenitor cells. Nat. Cell Biol..

[190] Bale AE (2002). Hedgehog signaling and human disease. Annu. Rev. Genom. Hum. Genet..

[191] Martí E, Bovolenta P (2002). Sonic hedgehog in CNS development: one signal, multiple outputs. Trends Neurosci..

[192] O’Hara WA (2011). Desert hedgehog is a mammal-specific gene expressed during testicular and ovarian development in a marsupial. BMC Dev. Biol..

[193] Long F (2004). Ihh signaling is directly required for the osteoblast lineage in the endochondral skeleton. Development.

[194] Ding M, Wang X (2017). Antagonism between Hedgehog and Wnt signaling pathways regulates tumorigenicity. Oncol. Lett..

[195] Gozal E, Jagadapillai R, Cai J, Barnes GN (2021). Potential crosstalk between sonic hedgehog-WNT signaling and neurovascular molecules: implications for blood-brain barrier integrity in autism spectrum disorder. J. Neurochem..

[196] Feng XH, Derynck R (2005). Specificity and versatility in tgf-beta signaling through Smads. Annu. Rev. Cell Dev. Biol..

[197] Guo X, Wang X-F (2009). Signaling cross-talk between TGF-β/BMP and other pathways. Cell Res..

[198] Liu Z (2006). A dishevelled-1/Smad1 interaction couples WNT and bone morphogenetic protein signaling pathways in uncommitted bone marrow stromal cells. J. Biol. Chem..

[199] Bernatik, O., Paclikova, P., Sri Ganji, R. & Bryja, V. Activity of Smurf2 ubiquitin ligase is regulated by the Wnt pathway protein dishevelled. *Cells* **9**, 1147 (2020). 10.3390/cells9051147 PMC7290506 32392721

[200] Hoppler S, Moon RT (1998). BMP-2/-4 and Wnt-8 cooperatively pattern the Xenopus mesoderm. Mech. Dev..

[201] Nishita M (2000). Interaction between Wnt and TGF-beta signalling pathways during formation of Spemann’s organizer. Nature.

[202] Song D (2021). Functional interaction between Wnt and Bmp signaling in periosteal bone growth. Sci. Rep..

[203] Bergenstock MK, Partridge NC (2007). Parathyroid hormone stimulation of noncanonical Wnt signaling in bone. Ann. N.Y. Acad. Sci..

[204] Wang Y (2014). Wnt and the Wnt signaling pathway in bone development and disease. Front. Biosci. (Landmark Ed).

[205] Lee M, Partridge NC (2009). Parathyroid hormone signaling in bone and kidney. Curr. Opin. Nephrol. Hypertens..

[206] Tamura Y, Kaji H (2013). Parathyroid hormone and Wnt signaling. Clin. Calcium.

[207] Leupin O (2007). Control of the SOST bone enhancer by PTH using MEF2 transcription factors. J. Bone Min. Res..

[208] Wein MN (2016). SIKs control osteocyte responses to parathyroid hormone. Nat. Commun..

[209] Sun N (2019). Effects of histone deacetylase inhibitor Scriptaid and parathyroid hormone on osteocyte functions and metabolism. J. Biol. Chem..

[210] Li C (2016). Lipoprotein receptor-related protein 6 is required for parathyroid hormone-induced Sost suppression. Ann. N.Y. Acad. Sci..

[211] Kulkarni N (2005). Effects of parathyroid hormone on Wnt signaling pathway in bone. J. Cell. Biochem..

[212] Guo X, Mak KK, Taketo MM, Yang Y (2009). The Wnt/beta-catenin pathway interacts differentially with PTHrP signaling to control chondrocyte hypertrophy and final maturation. PLoS One.

[213] Matthews J, Gustafsson JA (2003). Estrogen signaling: a subtle balance between ER alpha and ER beta. Mol. Int..

[214] Mirza FS, Padhi ID, Raisz LG, Lorenzo JA (2010). Serum sclerostin levels negatively correlate with parathyroid hormone levels and free estrogen index in postmenopausal women. J. Clin. Endocrinol. Metab..

[215] Kim RY (2015). Estrogen modulates bone morphogenetic protein-induced sclerostin expression through the Wnt signaling pathway. Tissue Eng. Part A.

[216] Armstrong VJ (2007). Wnt/beta-catenin signaling is a component of osteoblastic bone cell early responses to load-bearing and requires estrogen receptor alpha. J. Biol. Chem..

[217] Liedert A (2010). Estrogen receptor and Wnt signaling interact to regulate early gene expression in response to mechanical strain in osteoblastic cells. Biochem. Biophys. Res. Commun..

[218] Liedert, A. et al. Effects of estrogen receptor and wnt signaling activation on mechanically induced bone formation in a mouse model of postmenopausal bone loss. *Int. J. Mol. Sci.* **21**, 8301 (2020). 10.3390/ijms21218301 PMC7663944 33167497

[219] Galea GL (2013). Estrogen receptor α mediates proliferation of osteoblastic cells stimulated by estrogen and mechanical strain, but their acute down-regulation of the Wnt antagonist Sost is mediated by estrogen receptor β. J. Biol. Chem..

[220] Suthon S (2022). Estrogen receptor alpha and NFATc1 bind to a bone mineral density-associated SNP to repress WNT5B in osteoblasts. Am. J. Hum. Genet..

[221] Franz-Odendaal TA (2011). Induction and patterning of intramembranous bone. Front. Biosci. (Landmark Ed).

[222] Karaplis, A. Embryonic development of bone and the molecular regulation of intramembranous and endochondral bone formation. *Princ. Bone Biol.* 33–58 (2002).

[223] Takada S (1994). Wnt-3a regulates somite and tailbud formation in the mouse embryo. Genes Dev..

[224] Vlashi R, Zhang X, Wu M, Chen G (2023). Wnt signaling: essential roles in osteoblast differentiation, bone metabolism and therapeutic implications for bone and skeletal disorders. Genes Dis..

[225] Zhou S (2008). Age-related intrinsic changes in human bone-marrow-derived mesenchymal stem cells and their differentiation to osteoblasts. Aging Cell.

[226] Kawai M, de Paula FJ, Rosen CJ (2012). New insights into osteoporosis: the bone-fat connection. J. Intern. Med..

[227] Day TF, Guo X, Garrettbeal L, Yang Y (2005). Wnt/β-catenin signaling in mesenchymal progenitors controls osteoblast and chondrocyte differentiation during vertebrate skeletogenesis. Dev. Cell.

[228] Hill TP (2005). Canonical Wnt/β-catenin signaling prevents osteoblasts from differentiating into chondrocytes. Dev. Cell.

[229] Wu M (2017). Cbfβ governs osteoblast-adipocyte lineage commitment through enhancing β-catenin signaling and suppressing adipogenesis gene expression. Proc. Natl. Acad. Sci. USA.

[230] Byun MR (2014). Canonical Wnt signalling activates TAZ through PP1A during osteogenic differentiation. Cell Death Differ..

[231] Hong JH (2005). TAZ, a transcriptional modulator of mesenchymal stem cell differentiation. Science.

[232] Qiu W, Chen L, Kassem M (2011). Activation of non-canonical Wnt/JNK pathway by Wnt3a is associated with differentiation fate determination of human bone marrow stromal (mesenchymal) stem cells. Biochem. Biophys. Res. Commun..

[233] Tu X (2007). Noncanonical Wnt signaling through G protein-linked PKCδ activation promotes bone formation. Dev. Cell.

[234] Okamoto M (2014). Noncanonical Wnt5a enhances Wnt/beta-catenin signaling during osteoblastogenesis. Sci. Rep..

[235] Gu Q (2018). Wnt5a/FZD4 mediates the mechanical stretch-induced osteogenic differentiation of bone mesenchymal stem cells. Cell Physiol. Biochem..

[236] Qi Y (2020). An oriented-collagen scaffold including Wnt5a promotes osteochondral regeneration and cartilage interface integration in a rabbit model. FASEB J..

[237] Deng Y (2022). Biomaterial-mediated presentation of wnt5a mimetic ligands enhances chondrogenesis and metabolism of stem cells by activating non-canonical Wnt signaling. Biomaterials.

[238] Yang, L. et al. Wnt7a promotes the osteogenic differentiation of human mesenchymal stem cells. *Int. J. Mol. Med.* **47**, 94 (2021). 10.3892/ijmm.2021.4927 PMC8041482 33846764

[239] Bennett CN (2005). Regulation of osteoblastogenesis and bone mass by Wnt10b. Proc. Natl. Acad. Sci. USA.

[240] Kang S (2007). Wnt signaling stimulates osteoblastogenesis of mesenchymal precursors by suppressing CCAAT/enhancer-binding protein alpha and peroxisome proliferator-activated receptor gamma. J. Biol. Chem..

[241] Stevens JR (2010). Wnt10b deficiency results in age-dependent loss of bone mass and progressive reduction of mesenchymal progenitor cells. J. Bone Min. Res..

[242] Cawthorn WP (2012). Wnt6, Wnt10a and Wnt10b inhibit adipogenesis and stimulate osteoblastogenesis through a beta-catenin-dependent mechanism. Bone.

[243] Han X, Li X, Zhong G, Liu Z (2017). Regulation of osteogenic differentiation by DNA methylation of the dishevelled gene in bone marrow mesenchymal stem cells. Am. J. Transl. Res..

[244] Matsushita Y (2020). A Wnt-mediated transformation of the bone marrow stromal cell identity orchestrates skeletal regeneration. Nat. Commun..

[245] Zhao X (2020). ZBP1 (DAI/DLM-1) promotes osteogenic differentiation while inhibiting adipogenic differentiation in mesenchymal stem cells through a positive feedback loop of Wnt/beta-catenin signaling. Bone Res..

[246] Hang K (2021). Knockdown of SERPINB2 enhances the osteogenic differentiation of human bone marrow mesenchymal stem cells via activation of the Wnt/beta-catenin signalling pathway. Stem Cell Res. Ther..

[247] Zhao, F. et al. Mesenchymal MACF1 facilitates SMAD7 nuclear translocation to drive bone formation. *Cells* **9**, 616 (2020). 10.3390/cells9030616 PMC7140458 32143362

[248] Wu X (2021). High-mobility group AT-Hook 1 mediates the role of nuclear factor I/X in osteogenic differentiation through activating canonical Wnt signaling. Stem Cells.

[249] Zhang Z (2021). Circ_FBLN1 promotes the proliferation and osteogenic differentiation of human bone marrow-derived mesenchymal stem cells by regulating let-7i-5p/FZD4 axis and Wnt/β-catenin pathway. J. Bioenerg. Biomembr..

[250] Rui S (2022). Phosphate promotes osteogenic differentiation through non-canonical Wnt signaling pathway in human mesenchymal stem cells. Bone.

[251] Haffner-Luntzer, M. et al. Wnt1 boosts fracture healing by enhancing bone formation in the fracture callus. *J. Bone Miner. Res.* **38**, 749–762 (2023). 10.1002/jbmr.4797 36891752

[252] Gaur T (2005). Canonical WNT signaling promotes osteogenesis by directly stimulating Runx2 gene expression. J. Biol. Chem..

[253] Tu X (2007). Noncanonical Wnt signaling through G protein-linked PKCdelta activation promotes bone formation. Dev. Cell.

[254] Friedman MS, Oyserman SM, Hankenson KD (2009). Wnt11 promotes osteoblast maturation and mineralization through R-spondin 2. J. Biol. Chem..

[255] Shin HR (2016). Pin1-mediated modification prolongs the nuclear retention of beta-catenin in Wnt3a-induced osteoblast differentiation. J. Biol. Chem..

[256] Alam I (2016). Osteoblast-specific overexpression of human WNT16 increases both cortical and trabecular bone mass and structure in mice. Endocrinology.

[257] Lawson LY (2022). Osteoblast-specific Wnt secretion Is required for skeletal homeostasis and loading-induced bone formation in adult mice. J. Bone Min. Res..

[258] Albers J (2011). Control of bone formation by the serpentine receptor Frizzled-9. J. Cell Biol..

[259] Sebastian A (2017). Wnt co-receptors Lrp5 and Lrp6 differentially mediate Wnt3a signaling in osteoblasts. PLoS One.

[260] Riddle RC (2013). Lrp5 and Lrp6 exert overlapping functions in osteoblasts during postnatal bone acquisition. PLoS One.

[261] Kato M (2002). Cbfa1-independent decrease in osteoblast proliferation, osteopenia, and persistent embryonic eye vascularization in mice deficient in Lrp5, a Wnt coreceptor. J. Cell Biol..

[262] Little RD (2002). A mutation in the LDL receptor-related protein 5 gene results in the autosomal dominant high-bone-mass trait. Am. J. Hum. Genet..

[263] Cui Y (2011). Lrp5 functions in bone to regulate bone mass. Nat. Med..

[264] Choi, H. Y., Dieckmann, M., Herz, J. & Niemeier, A. Lrp4, a novel receptor for dickkopf 1 and sclerostin, is expressed by osteoblasts and regulates bone growth and turnover in vivo. *PLoS One* **4**, e7930 (2009). 10.1371/journal.pone.0007930 PMC2775917 19936252

[265] Simon-Chazottes D (2006). Mutations in the gene encoding the low-density lipoprotein receptor LRP4 cause abnormal limb development in the mouse. Genomics.

[266] Chang MK (2014). Disruption of Lrp4 function by genetic deletion or pharmacological blockade increases bone mass and serum sclerostin levels. Proc. Natl. Acad. Sci. USA.

[267] Zhang J (2012). LRP8 mediates Wnt/beta-catenin signaling and controls osteoblast differentiation. J. Bone Min. Res..

[268] Zhou F (2016). Ubiquitin-specific protease 4 antagonizes osteoblast differentiation through dishevelled. J. Bone Min. Res..

[269] Rodda SJ, McMahon AP (2006). Distinct roles for Hedgehog and canonical Wnt signaling in specification, differentiation and maintenance of osteoblast progenitors. Development.

[270] Song L (2012). Loss of Wnt/β-catenin signaling causes cell fate shift of preosteoblasts from osteoblasts to adipocytes. J. Bone Min. Res..

[271] Holmen SL (2005). Essential role of beta-catenin in postnatal bone acquisition. J. Biol. Chem..

[272] Glass DA (2005). Canonical Wnt signaling in differentiated osteoblasts controls osteoclast differentiation. Dev. Cell.

[273] Sharma AR (2013). Rspo 1 promotes osteoblast differentiation via Wnt signaling pathway. Indian J. Biochem. Biophys..

[274] Knight MN (2018). R-spondin-2 is a Wnt agonist that regulates osteoblast activity and bone mass. Bone Res..

[275] Hu L (2015). Knockdown of microtubule actin crosslinking factor 1 inhibits cell proliferation in MC3T3-E1 osteoblastic cells. BMB Rep..

[276] Hu L (2021). MACF1 promotes osteoblast differentiation by sequestering repressors in cytoplasm. Cell Death Differ..

[277] Yin C (2021). MACF1 alleviates aging-related osteoporosis via HES1. J. Cell Mol. Med..

[278] Bodine PV (2004). The Wnt antagonist secreted frizzled-related protein-1 is a negative regulator of trabecular bone formation in adult mice. Mol. Endocrinol..

[279] Yao W (2010). Overexpression of secreted frizzled-related protein 1 inhibits bone formation and attenuates parathyroid hormone bone anabolic effects. J. Bone Min. Res..

[280] Morello R (2008). Brachy-syndactyly caused by loss of Sfrp2 function. J. Cell Physiol..

[281] Nakanishi R (2008). Osteoblast-targeted expression of Sfrp4 in mice results in low bone mass. J. Bone Min. Res..

[282] Li J (2006). Dkk1-mediated inhibition of Wnt signaling in bone results in osteopenia. Bone.

[283] Morvan F (2006). Deletion of a single allele of the Dkk1 gene leads to an increase in bone formation and bone mass. J. Bone Min. Res..

[284] Hiramitsu S, Terauchi M, Kubota T (2013). The effects of Dickkopf-4 on the proliferation, differentiation, and apoptosis of osteoblasts. Endocrinology.

[285] Li X (2005). Dkk2 has a role in terminal osteoblast differentiation and mineralized matrix formation. Nat. Genet..

[286] Schulze J (2010). Negative regulation of bone formation by the transmembrane Wnt antagonist Kremen-2. PLoS One.

[287] ten Dijke P (2008). Osteocyte-derived sclerostin inhibits bone formation: its role in bone morphogenetic protein and Wnt signaling. J. Bone Jt. Surg. Am..

[288] Sutherland MK (2004). Sclerostin promotes the apoptosis of human osteoblastic cells: a novel regulation of bone formation. Bone.

[289] Loots GG (2005). Genomic deletion of a long-range bone enhancer misregulates sclerostin in Van Buchem disease. Genome Res..

[290] Li X (2008). Targeted deletion of the sclerostin gene in mice results in increased bone formation and bone strength. J. Bone Min. Res..

[291] Lin C (2009). Sclerostin mediates bone response to mechanical unloading through antagonizing Wnt/beta-catenin signaling. J. Bone Min. Res..

[292] Sebastian A, Loots GG (2018). Genetics of Sost/SOST in sclerosteosis and van Buchem disease animal models. Metabolism.

[293] Tang CY (2021). Runx1 is a central regulator of osteogenesis for bone homeostasis by orchestrating BMP and WNT signaling pathways. PLoS Genet..

[294] Han L (2021). Loss of chemerin triggers bone remodeling in vivo and in vitro. Mol. Metab..

[295] Gonzalez-Perez V, Lingle CJ (2019). Regulation of BK channels by beta and gamma subunits. Annu. Rev. Physiol..

[296] Jiang L (2021). BK channel deficiency in osteoblasts reduces bone formation via the Wnt/beta-catenin pathway. Mol. Cells.

[297] Li, H. et al. MiR-12200-5p Targets multiple members of Wnt signaling pathway to inhibit osteoblast differentiation and bone formation. *Endocr. Metab. Immune Disord. Drug Targets* **23**,1254–1264 (2023). 10.2174/1871530323666230301150350 36856174

[298] Karner CM, Long F (2017). Wnt signaling and cellular metabolism in osteoblasts. Cell Mol. Life Sci..

[299] Delgado-Calle J, Bellido T (2022). The osteocyte as a signaling cell. Physiol. Rev..

[300] Klein-Nulend J (2013). Mechanosensation and transduction in osteocytes. Bone.

[301] Zhou Y (2019). Aberrant activation of Wnt signaling pathway altered osteocyte mineralization. Bone.

[302] Osorio J (2015). Osteocyte-specific activation of the canonical Wnt-beta catenin pathway stimulates bone formation. Nat. Rev. Endocrinol..

[303] Kramer I (2010). Osteocyte Wnt/beta-catenin signaling is required for normal bone homeostasis. Mol. Cell Biol..

[304] Joeng KS (2017). Osteocyte-specific WNT1 regulates osteoblast function during bone homeostasis. J. Clin. Investig..

[305] Zhao L (2013). Inactivation of Lrp5 in osteocytes reduces young’s modulus and responsiveness to the mechanical loading. Bone.

[306] Javaheri B (2014). Deletion of a single beta-catenin allele in osteocytes abolishes the bone anabolic response to loading. J. Bone Min. Res..

[307] Tu X (2015). Osteocytes mediate the anabolic actions of canonical Wnt/beta-catenin signaling in bone. Proc. Natl. Acad. Sci. USA.

[308] Weidauer SE (2009). NMR structure of the Wnt modulator protein Sclerostin. Biochem. Biophys. Res. Commun..

[309] Poole KE (2005). Sclerostin is a delayed secreted product of osteocytes that inhibits bone formation. FASEB J..

[310] Robling AG (2008). Mechanical stimulation of bone in vivo reduces osteocyte expression of Sost/sclerostin. J. Biol. Chem..

[311] Tu X (2012). Sost downregulation and local Wnt signaling are required for the osteogenic response to mechanical loading. Bone.

[312] Vaananen HK, Laitalaleinonen T (2008). Osteoclast lineage and function. Arch. Biochem. Biophys..

[313] Otero K (2012). TREM2 and beta-catenin regulate bone homeostasis by controlling the rate of osteoclastogenesis. J. Immunol..

[314] Wei W (2011). Biphasic and dosage-dependent regulation of osteoclastogenesis by beta-catenin. Mol. Cell Biol..

[315] Ruiz P (2016). CathepsinKCre mediated deletion of betacatenin results in dramatic loss of bone mass by targeting both osteoclasts and osteoblastic cells. Sci. Rep..

[316] Romero G (2010). Parathyroid hormone receptor directly interacts with dishevelled to regulate beta-Catenin signaling and osteoclastogenesis. J. Biol. Chem..

[317] Weivoda MM (2016). Wnt signaling inhibits osteoclast differentiation by activating canonical and noncanonical cAMP/PKA pathways. J. Bone Min. Res..

[318] Chen K (2019). Sfrp4 repression of the Ror2/Jnk cascade in osteoclasts protects cortical bone from excessive endosteal resorption. Proc. Natl. Acad. Sci. USA.

[319] Spencer GJ (2006). Wnt signalling in osteoblasts regulates expression of the receptor activator of NFkappaB ligand and inhibits osteoclastogenesis in vitro. J. Cell Sci..

[320] Fujita K, Janz S (2007). Attenuation of WNT signaling by DKK-1 and -2 regulates BMP2-induced osteoblast differentiation and expression of OPG, RANKL and M-CSF. Mol. Cancer.

[321] Albers J (2013). Canonical Wnt signaling inhibits osteoclastogenesis independent of osteoprotegerin. J. Cell Biol..

[322] Maeda K (2012). Wnt5a-Ror2 signaling between osteoblast-lineage cells and osteoclast precursors enhances osteoclastogenesis. Nat. Med..

[323] Kobayashi Y, Uehara S, Koide M, Takahashi N (2015). The regulation of osteoclast differentiation by Wnt signals. Bonekey Rep..

[324] Uehara, S. et al. Protein kinase N3 promotes bone resorption by osteoclasts in response to Wnt5a-Ror2 signaling. *Sci Signal* **10** (2017). 10.1126/scisignal.aan0023 28851822

[325] Uehara S, Udagawa N, Kobayashi Y (2018). Non-canonical Wnt signals regulate cytoskeletal remodeling in osteoclasts. Cell Mol. Life Sci..

[326] Movérare-Skrtic S (2014). Osteoblast-derived WNT16 represses osteoclastogenesis and prevents cortical bone fragility fractures. Nat. Med..

[327] Qiang YW (2010). Characterization of Wnt/beta-catenin signalling in osteoclasts in multiple myeloma. Br. J. Haematol..

[328] Baron R, Kneissel M (2013). WNT signaling in bone homeostasis and disease: from human mutations to treatments. Nat. Med..

[329] Cheng J, Li M, Bai R (2022). The Wnt signaling cascade in the pathogenesis of osteoarthritis and related promising treatment strategies. Front. Physiol..

[330] Cici, D., Corrado, A., Rotondo, C. & Cantatore, F. P. Wnt signaling and biological therapy in rheumatoid arthritis and spondyloarthritis. *Int. J. Mol. Sci.* **20** (2019). 10.3390/ijms20225552 PMC6888549 31703281

[331] van Andel H, Kocemba KA, Spaargaren M, Pals ST (2019). Aberrant Wnt signaling in multiple myeloma: molecular mechanisms and targeting options. Leukemia.

[332] Yuan Y, Guo M, Gu C, Yang Y (2021). The role of Wnt/β-catenin signaling pathway in the pathogenesis and treatment of multiple myeloma (review). Am. J. Transl. Res..

[333] Laine CM (2013). WNT1 mutations in early-onset osteoporosis and osteogenesis imperfecta. N. Engl. J. Med..

[334] Fahiminiya S (2013). Mutations in WNT1 are a cause of osteogenesis imperfecta. J. Med. Genet..

[335] Hu, J. et al. Genotypic and phenotypic spectrum and pathogenesis of WNT1 variants in a large cohort of patients with OI/osteoporosis. *J. Clin. Endocrinol. Metab.* **108**, 1776-1786 (2023). 10.1210/clinem/dgac752 PMC10271228 36595228

[336] Peris P (2023). Osteoporosis related to WNT1 variants: a not infrequent cause of osteoporosis. Osteoporos. Int..

[337] Niemann S (2004). Homozygous WNT3 mutation causes tetra-amelia in a large consanguineous family. Am. J. Hum. Genet..

[338] Zheng HF (2012). WNT16 influences bone mineral density, cortical bone thickness, bone strength, and osteoporotic fracture risk. PLoS Genet..

[339] Jing H (2018). Epigenetic inhibition of Wnt pathway suppresses osteogenic differentiation of BMSCs during osteoporosis. Cell Death Dis..

[340] Gong Y (2001). LDL receptor-related protein 5 (LRP5) affects bone accrual and eye development. Cell.

[341] Beighton P (1986). Osteoporosis-pseudoglioma syndrome. Clin. Genet..

[342] Astiazaran MC (2017). Novel homozygous LRP5 mutations in Mexican patients with osteoporosis-pseudoglioma syndrome. Genet Test. Mol. Biomark..

[343] Kamizaki K, Endo M, Minami Y, Kobayashi Y (2021). Role of noncanonical Wnt ligands and Ror-family receptor tyrosine kinases in the development, regeneration, and diseases of the musculoskeletal system. Dev. Dyn..

[344] Caetano da Silva C (2022). WNT11, a new gene associated with early onset osteoporosis, is required for osteoblastogenesis. Hum. Mol. Genet..

[345] Boyden LM (2002). High bone density due to a mutation in LDL-receptor-related protein 5. N. Engl. J. Med..

[346] Fijalkowski I (2016). A novel domain-specific mutation in a sclerosteosis patient suggests a role of LRP4 as an anchor for sclerostin in human bone. J. Bone Min. Res..

[347] Loeser RF, Goldring SR, Scanzello CR, Goldring MB (2012). Osteoarthritis: a disease of the joint as an organ. Arthritis Rheum..

[348] Shi S (2016). Silencing of Wnt5a prevents interleukin-1β-induced collagen type II degradation in rat chondrocytes. Exp. Ther. Med..

[349] Huang G, Chubinskaya S, Liao W, Loeser RF (2017). Wnt5a induces catabolic signaling and matrix metalloproteinase production in human articular chondrocytes. Osteoarthr. Cartil..

[350] Martineau X (2017). Alteration of Wnt5a expression and of the non-canonical Wnt/PCP and Wnt/PKC-Ca^2+^ pathways in human osteoarthritis osteoblasts. PLoS One.

[351] Hopwood B, Tsykin A, Findlay DM, Fazzalari NL (2007). Microarray gene expression profiling of osteoarthritic bone suggests altered bone remodelling, WNT and transforming growth factor-beta/bone morphogenic protein signalling. Arthritis Res. Ther..

[352] Shi FL, Ren LX (2020). Up-regulated miR-374a-3p relieves lipopolysaccharides induced injury in CHON-001 cells via regulating Wingless-type MMTV integration site family member 5B. Mol. Cell Probes.

[353] Huang J (2020). Dysregulation of the Wnt signaling pathway and synovial stem cell dysfunction in osteoarthritis development. Stem Cells Dev..

[354] Gibson AL (2017). Wnt7a inhibits IL-1β induced catabolic gene expression and prevents articular cartilage damage in experimental osteoarthritis. Sci. Rep..

[355] Ye X, Liu X (2022). Wnt16 signaling in bone homeostasis and osteoarthristis. Front. Endocrinol..

[356] Nalesso G (2017). WNT16 antagonises excessive canonical WNT activation and protects cartilage in osteoarthritis. Ann. Rheum. Dis..

[357] Tong W (2019). Wnt16 attenuates osteoarthritis progression through a PCP/JNK-mTORC1-PTHrP cascade. Ann. Rheum. Dis..

[358] Smith AJ (2005). Haplotypes of the low-density lipoprotein receptor-related protein 5 (LRP5) gene: are they a risk factor in osteoarthritis?. Osteoarthr. Cartil..

[359] Yerges-Armstrong LM (2014). Association analysis of BMD-associated SNPs with knee osteoarthritis. J. Bone Min. Res..

[360] Joiner DM (2013). Heterozygosity for an inactivating mutation in low-density lipoprotein-related receptor 6 (Lrp6) increases osteoarthritis severity in mice after ligament and meniscus injury. Osteoarthr. Cartil..

[361] Zhu M (2009). Activation of β-catenin signaling in articular chondrocytes leads to osteoarthritis-like phenotype in adult β-catenin conditional activation mice. J. Bone Min. Res..

[362] Xia C (2019). Activation of β-catenin in Col2-expressing chondrocytes leads to osteoarthritis-like defects in hip joint. J. Cell Physiol..

[363] Hui T (2018). Activation of β-catenin signaling in aggrecan-expressing cells in temporomandibular joint causes osteoarthritis-like defects. Int. J. Oral. Sci..

[364] Lu K (2023). Upregulation of β-catenin signaling represents a single common pathway leading to the various phenotypes of spinal degeneration and pain. Bone Res..

[365] Zhu Z (2022). AMPK activator decelerates osteoarthritis development by inhibition of β-catenin signaling in chondrocytes. J. Orthop. Transl..

[366] Li J (2020). Metformin limits osteoarthritis development and progression through activation of AMPK signalling. Ann. Rheum. Dis..

[367] Xuan F (2019). Wnt/β-catenin signaling contributes to articular cartilage homeostasis through lubricin induction in the superficial zone. Arthritis Res. Ther..

[368] Weng LH (2009). Inflammation induction of Dickkopf-1 mediates chondrocyte apoptosis in osteoarthritic joint. Osteoarthr. Cartil..

[369] Weng LH (2010). Control of Dkk-1 ameliorates chondrocyte apoptosis, cartilage destruction, and subchondral bone deterioration in osteoarthritic knees. Arthritis Rheum..

[370] Oh H, Chun CH, Chun JS (2012). Dkk-1 expression in chondrocytes inhibits experimental osteoarthritic cartilage destruction in mice. Arthritis Rheum..

[371] Theologis T (2017). Association between serum and synovial fluid Dickkopf-1 levels with radiographic severity in primary knee osteoarthritis patients. Clin. Rheumatol..

[372] Zhu Z (2020). A study on the mechanism of Wnt inhibitory factor 1 in osteoarthritis. Arch. Med. Sci..

[373] Chin KY, Ekeuku SO, Pang KL (2022). Sclerostin in the development of osteoarthritis: a mini review. Malays. J. Pathol..

[374] Zhang Y (2022). Runx1 is a key regulator of articular cartilage homeostasis by orchestrating YAP, TGFβ, and Wnt signaling in articular cartilage formation and osteoarthritis. Bone Res..

[375] Sen M (2001). Blockade of Wnt-5A/frizzled 5 signaling inhibits rheumatoid synoviocyte activation. Arthritis Rheum..

[376] Sen M, Carson DA (2002). Wnt signaling in rheumatoid synoviocyte activation. Mod. Rheumatol..

[377] Lories RJ, Corr M, Lane NE (2013). To Wnt or not to Wnt: the bone and joint health dilemma. Nat. Rev. Rheumatol..

[378] Sen M (2000). Expression and function of wingless and frizzled homologs in rheumatoid arthritis. Proc. Natl. Acad. Sci. USA.

[379] Miao P (2018). Regulatory effect of anti-gp130 functional mAb on IL-6 mediated RANKL and Wnt5a expression through JAK-STAT3 signaling pathway in FLS. Oncotarget.

[380] Rodriguez-Trillo A (2020). Non-canonical WNT5A signaling through RYK contributes to aggressive phenotype of the rheumatoid fibroblast-like synoviocytes. Front. Immunol..

[381] Huang Y (2023). Wnt5a: a promising therapeutic target for inflammation, especially rheumatoid arthritis. Cytokine.

[382] de Rooy DP (2013). Genetic studies on components of the Wnt signalling pathway and the severity of joint destruction in rheumatoid arthritis. Ann. Rheum. Dis..

[383] Singh A, Gupta MK, Mishra SP (2019). Study of correlation of level of expression of Wnt signaling pathway inhibitors sclerostin and dickkopf-1 with disease activity and severity in rheumatoid arthritis patients. Drug Discov. Ther..

[384] Wang SY (2011). Circulating Dickkopf-1 is correlated with bone erosion and inflammation in rheumatoid arthritis. J. Rheumatol..

[385] Wehmeyer C (2016). Sclerostin inhibition promotes TNF-dependent inflammatory joint destruction. Sci. Transl. Med..

[386] Fassio A (2019). Inhibition of tumor necrosis factor-alpha (TNF-alpha) in patients with early rheumatoid arthritis results in acute changes of bone modulators. Int Immunopharmacol..

[387] Adami G (2016). Effects of TNF inhibitors on parathyroid hormone and Wnt signaling antagonists in rheumatoid arthritis. Calcif. Tissue Int..

[388] Miao, C. et al. DNA hypermethylation of SFRP2 influences the pathology of rheumatoid arthritis through the canonical Wnt signaling in model rats. *Autoimmunity* 1–14 (2018). 10.1080/08916934.2018.1516760 10.1080/08916934.2018.1516760 30345838

[389] Miao CG (2013). MeCP2 modulates the canonical Wnt pathway activation by targeting SFRP4 in rheumatoid arthritis fibroblast-like synoviocytes in rats. Cell Signal.

[390] Kwon YJ (2014). Secreted frizzled-related protein 5 suppresses inflammatory response in rheumatoid arthritis fibroblast-like synoviocytes through down-regulation of c-Jun N-terminal kinase. Rheumatology.

[391] Xu Y (2022). Acid sensor ASIC1a induces synovial fibroblast proliferation via Wnt/β-catenin/c-Myc pathway in rheumatoid arthritis. Int. Immunopharmacol..

[392] Zhang Z (2021). PLD1 knockdown reduces metastasis and inflammation of fibroblast-like synoviocytes in rheumatoid arthritis by modulating NF-κB and Wnt/β-catenin pathways. Autoimmunity.

[393] Zhou MY (2021). Lentivirus-mediated overexpression or silencing of aquaporin 1 affects the proliferation, migration and invasion of TNF-α-stimulated rheumatoid arthritis fibroblast-like synoviocytes by Wnt/β-catenin signaling pathway. J. Inflamm. Res..

[394] Wang R (2021). NAV2 positively modulates inflammatory response of fibroblast-like synoviocytes through activating Wnt/β-catenin signaling pathway in rheumatoid arthritis. Clin. Transl. Med..

[395] Sun Y (2023). LncRNA OIP5-AS1/miR-410-3p/Wnt7b axis promotes the proliferation of rheumatoid arthritis fibroblast-like synoviocytes via regulating the Wnt/β-catenin pathway. Autoimmunity.

[396] Wang, W. et al. FOXM1/LINC00152 feedback loop regulates proliferation and apoptosis in rheumatoid arthritis fibroblast-like synoviocytes via Wnt/β-catenin signaling pathway. *Biosci. Rep.* **40**, BSR20191900 (2020). 10.1042/BSR20191900 PMC6974425 31854447

[397] Wang Y (2021). MiR-125a-3p inhibits cell proliferation and inflammation responses in fibroblast-like synovial cells in rheumatoid arthritis by mediating the Wnt/β-catenin and NF-κB pathways via targeting MAST3. J. Musculoskelet. Neuronal Interact..

[398] Yu B (2014). Wnt4 signaling prevents skeletal aging and inflammation by inhibiting nuclear factor-κB. Nat. Med..

[399] Jiang Y (2016). Effect of pulsed electromagnetic field on bone formation and lipid metabolism of glucocorticoid-induced osteoporosis rats through canonical Wnt signaling pathway. Evid. Based Complement. Altern. Med..

[400] Fan H (2016). Electroacupuncture stimulation at CV4 prevents ovariectomy-induced osteoporosis in rats via Wnt-beta-catenin signaling. Mol. Med. Rep..

[401] Diegel CR (2023). Inhibiting WNT secretion reduces high bone mass caused by Sost loss-of-function or gain-of-function mutations in Lrp5. Bone Res..

[402] Yazici Y (2017). A novel Wnt pathway inhibitor, SM04690, for the treatment of moderate to severe osteoarthritis of the knee: results of a 24-week, randomized, controlled, phase 1 study. Osteoarthr. Cartil..

[403] Yazici Y (2021). A phase 2b randomized trial of lorecivivint, a novel intra-articular CLK2/DYRK1A inhibitor and Wnt pathway modulator for knee osteoarthritis. Osteoarthr. Cartil..

[404] Tambiah JRS (2021). Individual participant symptom responses to intra-articular lorecivivint in knee osteoarthritis: post hoc analysis of a phase 2B trial. Rheumatol. Ther..

[405] Tambiah JRS (2022). Comparing patient-reported outcomes from sham and saline-based placebo injections for knee osteoarthritis: data from a randomized clinical trial of lorecivivint. Am. J. Sports Med..

[406] MacLauchlan S (2017). Genetic deficiency of Wnt5a diminishes disease severity in a murine model of rheumatoid arthritis. Arthritis Res. Ther..

[407] Cao W (2018). Depleting the carboxy-terminus of human Wnt5a attenuates collagen-induced arthritis in DBA/1 mice. Biochem. Biophys. Res. Commun..

[408] Rodríguez-Trillo A (2022). ROCK inhibition with Y-27632 reduces joint inflammation and damage in serum-induced arthritis model and decreases in vitro osteoclastogenesis in patients with early arthritis. Front. Immunol..

[409] Xie C (2020). Ginkgolide B attenuates collagen-induced rheumatoid arthritis and regulates fibroblast-like synoviocytes-mediated apoptosis and inflammation. Ann. Transl. Med..

[410] Oz B (2019). Resveratrol inhibits Src tyrosine kinase, STAT3, and Wnt signaling pathway in collagen induced arthritis model. Biofactors.

[411] Liu XG (2019). MiR-21 relieves rheumatoid arthritis in rats via targeting Wnt signaling pathway. Eur. Rev. Med. Pharm. Sci..

[412] Fowler TW (2021). Development of selective bispecific Wnt mimetics for bone loss and repair. Nat. Commun..

[413] Okuchi Y (2021). Wnt-modified materials mediate asymmetric stem cell division to direct human osteogenic tissue formation for bone repair. Nat. Mater..

[414] Wang FS (2007). Knocking down dickkopf-1 alleviates estrogen deficiency induction of bone loss. A histomorphological study in ovariectomized rats. Bone.

[415] Wang FS (2008). Modulation of Dickkopf-1 attenuates glucocorticoid induction of osteoblast apoptosis, adipocytic differentiation, and bone mass loss. Endocrinology.

[416] Peng, Z. et al. Exosomes from bone marrow mesenchymal stem cells promoted osteogenic differentiation by delivering miR-196a that targeted Dickkopf-1 to activate Wnt/β-catenin pathway. *Bioengineered* **14**, 1996015 (2021). 10.1080/21655979.2021.1996015 PMC10501159 34720039

[417] Zhou B (2020). miR‑483‑3p promotes the osteogenesis of human osteoblasts by targeting Dikkopf 2 (DKK2) and the Wnt signaling pathway. Int. J. Mol. Med..

[418] Gao J (2021). Icariin promotes the osteogenesis of bone marrow mesenchymal stem cells through regulating sclerostin and activating the Wnt/β-catenin signaling pathway. Biomed. Res. Int..

[419] Marini, F., Giusti, F., Palmini, G. & Brandi, M. L. Role of Wnt signaling and sclerostin in bone and as therapeutic targets in skeletal disorders. *Osteoporosis Int.* **34**, 213–238 (2022). 10.1007/s00198-022-06523-7 35982318

[420] Kerschan-Schindl K (2020). Romosozumab: a novel bone anabolic treatment option for osteoporosis?. Wien. Med. Wochenschr..

[421] Sølling ASK, Harsløf T, Langdahl B (2018). The clinical potential of romosozumab for the prevention of fractures in postmenopausal women with osteoporosis. Ther. Adv. Musculoskelet. Dis..

[422] McClung MR (2014). Romosozumab in postmenopausal women with low bone mineral density. N. Engl. J. Med..

[423] Cosman F (2016). Romosozumab treatment in postmenopausal women with osteoporosis. N. Engl. J. Med..

[424] Eriksen EF (2022). Modeling-based bone bormation after 2 months of romosozumab treatment: results from the FRAME clinical trial. J. Bone Min. Res..

[425] Langdahl BL (2017). Romosozumab (sclerostin monoclonal antibody) versus teriparatide in postmenopausal women with osteoporosis transitioning from oral bisphosphonate therapy: a randomised, open-label, phase 3 trial. Lancet.

[426] Lewiecki EM (2018). A phase III randomized placebo-controlled trial to evaluate efficacy and safety of romosozumab in men with osteoporosis. J. Clin. Endocrinol. Metab..

[427] Sangadala, S. et al. Sclerostin small-molecule inhibitors promote osteogenesis by activating canonical Wnt and BMP pathways. *Elife* **12,** e63402 (2023). 10.7554/eLife.63402 PMC10431921 37560905

[428] Florio M (2016). A bispecific antibody targeting sclerostin and DKK-1 promotes bone mass accrual and fracture repair. Nat. Commun..

[429] Choi RB (2021). Improving bone health by optimizing the anabolic action of Wnt inhibitor multitargeting. JBMR.

[430] Melnik S (2021). MiR-181a targets RSPO2 and regulates bone morphogenetic protein—WNT signaling crosstalk during chondrogenic differentiation of mesenchymal stromal cells. Front. Cell Dev. Biol..

[431] Chen Y (2007). Beta-catenin signaling plays a disparate role in different phases of fracture repair: implications for therapy to improve bone healing. PLoS Med..

[432] Clément-Lacroix P (2005). Lrp5-independent activation of Wnt signaling by lithium chloride increases bone formation and bone mass in mice. Proc. Natl. Acad. Sci. USA.

[433] Zhang K (2023). MACF1 overexpression in BMSCs alleviates senile osteoporosis in mice through TCF4/miR-335-5p signaling pathway. J. Orthop. Transl..

[434] Wang Y (2020). Daphnetin ameliorates glucocorticoid-induced osteoporosis via activation of Wnt/GSK-3β/β-catenin signaling. Toxicol. Appl. Pharm..

[435] Jiang, H. et al. Gentiopicroside promotes the osteogenesis of bone mesenchymal stem cells by modulation of β-catenin-BMP2 signalling pathway. *J. Cell Mol. Med.* **25**, 10825–10836 (2021). 10.1111/jcmm.16410 PMC8642693 34783166

[436] Karunarathne W (2021). Anthocyanin-enriched polyphenols from Hibiscus syriacus L. (Malvaceae) exert anti-osteoporosis effects by inhibiting GSK-3β and subsequently activating β-catenin. Phytomedicine.

[437] Yang X (2021). Troxerutin stimulates osteoblast differentiation of mesenchymal stem cell and facilitates bone fracture healing. Front. Pharm..

[438] Kim HY (2016). Small molecule inhibitors of the Dishevelled-CXXC5 interaction are new drug candidates for bone anabolic osteoporosis therapy. EMBO Mol. Med..

[439] Pan FF (2021). Apigenin promotes osteogenic differentiation of mesenchymal stem cells and accelerates bone fracture healing via activating Wnt/β-catenin signaling. Am. J. Physiol. Endocrinol. Metab..

[440] Bai J (2021). Glycyrrhizic acid promotes osteogenic differentiation of human bone marrow stromal cells by activating the Wnt/β-catenin signaling pathway. Front. Pharm..

[441] Wei Y (2021). miR-424-5p shuttled by bone marrow stem cells-derived exosomes attenuates osteogenesis via regulating WIF1-mediated Wnt/β-catenin axis. Aging.

[442] Hu H (2021). Role of microRNA-335 carried by bone marrow mesenchymal stem cells-derived extracellular vesicles in bone fracture recovery. Cell Death Dis..

[443] Yu H, Zhang J, Liu X, Li Y (2021). microRNA-136-5p from bone marrow mesenchymal stem cell-derived exosomes facilitates fracture healing by targeting LRP4 to activate the Wnt/β-catenin pathway. Bone Jt. Res..

[444] Liu X (2019). Exosomes derived from platelet-rich plasma present a novel potential in alleviating knee osteoarthritis by promoting proliferation and inhibiting apoptosis of chondrocyte via Wnt/β-catenin signaling pathway. J. Orthop. Surg. Res..

[445] Dong J, Li L, Fang X, Zang M (2021). Exosome-encapsulated microRNA-127-3p released from bone marrow-derived mesenchymal stem cells alleviates osteoarthritis through regulating CDH11-mediated Wnt/β-catenin pathway. J. Pain. Res..

[446] Chen G (2021). PiRNA-63049 inhibits bone formation through Wnt/β-catenin signaling pathway. Int. J. Biol. Sci..

[447] Luo Y (2019). The osteogenic differentiation of human adipose-derived stem cells is regulated through the let-7i-3p/LEF1/β-catenin axis under cyclic strain. Stem Cell Res. Ther..

[448] Yin C (2020). miR-129-5p inhibits bone formation through TCF4. Front. Cell Dev. Biol..

[449] Hu S (2019). MicroRNA-320c inhibits development of osteoarthritis through downregulation of canonical Wnt signaling pathway. Life Sci..

[450] Rawadi G (2008). Wnt signaling and potential applications in bone diseases. Curr. Drug Targets.

[451] Mullard A (2019). FDA approves first-in-class osteoporosis drug. Nat. Rev. Drug Discov..

[452] Chen KH (2015). RNA imaging. Spatially resolved, highly multiplexed RNA profiling in single cells. Science.

[453] Barrow JR (2003). Ectodermal Wnt3/beta-catenin signaling is required for the establishment and maintenance of the apical ectodermal ridge. Genes Dev..

[454] Spater D (2006). Wnt9a signaling is required for joint integrity and regulation of Ihh during chondrogenesis. Development.

[455] Lee, H. H. & Behringer, R. R. Conditional expression of Wnt4 during chondrogenesis leads to dwarfism in mice. *PLoS One* **2,** e450 (2007). 10.1371/journal.pone.0000450 PMC1865390 17505543

[456] Yamaguchi TP, Bradley A, McMahon AP, Jones S (1999). A Wnt5a pathway underlies outgrowth of multiple structures in the vertebrate embryo. Development.

[457] Yang Y, Topol L, Lee H, Wu J (2003). Wnt5a and Wnt5b exhibit distinct activities in coordinating chondrocyte proliferation and differentiation. Development.

[458] Parr BA, Mc Mahon AP (1995). Dorsalizing signal Wnt-7a required for normal polarity of D−V and A−P axes of mouse limb. Nature.

[459] Pöpperl H (1997). Misexpression of Cwnt8C in the mouse induces an ectopic embryonic axis and causes a truncation of the anterior neuroectoderm. Development.

[460] Guo X (2004). Wnt/beta-catenin signaling is sufficient and necessary for synovial joint formation. Genes Dev..

[461] Carroll TJ (2005). Wnt9b plays a central role in the regulation of mesenchymal to epithelial transitions underlying organogenesis of the mammalian urogenital system. Dev. Cell.

[462] Jin YR, Han XH, Taketo MM, Yoon JK (2012). Wnt9b-dependent FGF signaling is crucial for outgrowth of the nasal and maxillary processes during upper jaw and lip development. Development.

[463] Bennett CN (2007). Wnt10b increases postnatal bone formation by enhancing osteoblast differentiation. J. Bone Min. Res..

[464] Johnson EB, Hammer RE, Herz J (2005). Abnormal development of the apical ectodermal ridge and polysyndactyly in Megf7-deficient mice. Hum. Mol. Genet..

[465] Holmen SL (2004). Decreased BMD and limb deformities in mice carrying mutations in both Lrp5 and Lrp6. J. Bone Miner. Res..

[466] Iwaniec UT (2007). PTH stimulates bone formation in mice deficient in Lrp5. J. Bone Miner. Res..

[467] Yadav VK (2008). Lrp5 controls bone formation by inhibiting serotonin synthesis in the duodenum. Cell.

[468] Joeng KS (2011). Lrp5 and Lrp6 redundantly control skeletal development in the mouse embryo. Dev. Biol..

[469] Niziolek PJ (2011). High-bone-mass-producing mutations in the Wnt signaling pathway result in distinct skeletal phenotypes. Bone.

[470] Kokubu C (2004). Skeletal defects in ringelschwanz mutant mice reveal that Lrp6 is required for proper somitogenesis and osteogenesis. Development.

[471] Mukhopadhyay M (2001). Dickkopf1 Is required for embryonic head induction and limb morphogenesis in the mouse. Dev. Cell.

[472] Yao GQ, Wu JJ, Troiano N, Insogna K (2011). Targeted overexpression of Dkk1 in osteoblasts reduces bone mass but does not impair the anabolic response to intermittent PTH treatment in mice. J. Bone Min. Metab..

[473] Oh H (2012). Misexpression of Dickkopf-1 in endothelial cells, but not in chondrocytes or hypertrophic chondrocytes, causes defects in endochondral ossification. J. Bone Miner. Res..

[474] Lories RJU (2007). Articular cartilage and biomechanical properties of the long bones in Frzb-knockout mice. Arthritis Rheumat..

[475] Cho HY (2010). Transgenic mice overexpressing secreted frizzled-related proteins (sFRP)4 under the control of serum amyloid P promoter exhibit low bone mass but did not result in disturbed phosphate homeostasis. Bone.

[476] Hoeflich KP (2000). Requirement for glycogen synthase kinase-3β in cell survival and NF-κB activation. Nature.

[477] Kugimiya, F. et al. GSK-3β controls osteogenesis through regulating Runx2 activity. *PLoS One* **2**, e837 (2007). 10.1371/journal.pone.0000837 PMC1950686 17786208

[478] Nelson, E. R. et al. Role of GSK-3β in the osteogenic differentiation of palatal mesenchyme. *PLoS One* **6**, e25847 (2011). 10.1371/journal.pone.0025847 PMC3194817 22022457

[479] He F (2010). Gsk3β is required in the epithelium for palatal elevation in mice. Dev. Dyn..

[480] Gillespie JR (2011). Deletion of glycogen synthase kinase-3β in cartilage results in up-regulation of glycogen synthase kinase-3α protein expression. Endocrinology.

[481] Itoh S (2012). GSK-3α and GSK-3β proteins are involved in early stages of chondrocyte differentiation with functional redundancy through RelA protein phosphorylation. J. Biol. Chem..

[482] Perry Iii WL (1995). Phenotypic and molecular analysis of a transgenic insertional allele of the mouse fused locus. Genetics.

[483] Yu HMI (2005). The role of Axin2 in calvarial morphogenesis and craniosynostosis. Development.

[484] Yan Y (2009). Axin2 controls bone remodeling through the β-catenin-BMP signaling pathway in adult mice. J. Cell Sci..

[485] Dao DY (2010). Axin2 regulates chondrocyte maturation and axial skeletal development. J. Orthop. Res..

[486] Holmen SL (2005). Essential role of β-catenin in postnatal bone acquisition. J. Biol. Chem..

[487] Miclea, R. L. et al. Adenomatous polyposis coli-mediated control of β-catenin is essential for both chondrogenic and osteogenic differentiation of skeletal precursors. *BMC Dev. Biol.* **9**, 26 (2009). 10.1186/1471-213X-9-26 PMC2678105 19356224

[488] Soshnikova N (2003). Genetic interaction between Wnt/β-catenin and BMP receptor signaling during formation of the AER and the dorsal-ventral axis in the limb. Genes Dev..

[489] Akiyama H (2004). Interactions between Sox9 and β-catenin control chondrocyte differentiation. Genes Dev..

[490] Dao DY (2012). Cartilage-specific β-catenin signaling regulates chondrocyte maturation, generation of ossification centers, and perichondrial bone formation during skeletal development. J. Bone Min. Res..

[491] Chen J, Long F (2012). Beta-catenin promotes bone formation and suppresses bone resorption in postnatal growing mice. J. Bone Min. Res..

[492] Mirando, A. J. et al. Β-catenin/cyclin D1 mediated development of suture mesenchyme in calvarial morphogenesis. *BMC Dev. Biol.* **10**, 116 (2010). 10.1186/1471-213X-10-116 PMC3001432 21108844

[493] Wei W (2011). Biphasic and dosage-dependent regulation of osteoclastogenesis by β-catenin. Mol. Cell. Biol..

[494] Kramer I (2010). Osteocyte Wnt/β-catenin signaling is required for normal bone homeostasis. Mol. Cell. Biol..

[495] Mikasa M (2011). Regulation of Tcf7 by Runx2 in chondrocyte maturation and proliferation. J. Bone Miner. Metab..

[496] Korinek V (1998). Depletion of epithelial stem-cell compartments in the small intestine of mice lacking Tcf-4. Nat. Genet..

[497] Brugmann SA (2007). Wnt signaling mediates regional specification in the vertebrate face. Development.

[498] Van Genderen C (1994). Development of several organs that require inductive epithelial- mesenchymal interactions is impaired in LEF-1-deficient mice. Genes Dev..

[499] Galceran J (1999). Wnt3a-/–like phenotype and limb deficiency in *Lef1*^*−/−*^*Tcf1*^*−/−*^ mice. Genes Dev..

[500] Noh T (2009). Lef1 haploinsufficient mice display a low turnover and low bone mass phenotype in a gender- and age-specific manner. PLoS One.

[501] Hoeppner LH (2011). Lef1ΔN binds β-catenin and increases osteoblast activity and trabecular bone mass. J. Biol. Chem..

